# Synthesis and Evaluation of Aromatic Sulfonamide and Disulfonamide Derivatives as Dengue Virus NS3–NS5 Interaction Inhibitors

**DOI:** 10.1002/cmdc.70337

**Published:** 2026-06-05

**Authors:** Jakub Janáč, Jan Drahorád, Jakub Harvalík, Tereza Horáčková, Manfred Brinker, Arianna Loregian, Andrea Brancale, Beatrice Mercorelli, Petra Cuřínová

**Affiliations:** ^1^ Department of Organic Chemistry University of Chemistry and Technology Prague Prague Czech Republic; ^2^ Department of Molecular Medicine University of Padua Padua Italy; ^3^ Microbiology and Virology Unit Padua University Hospital Padua Italy

**Keywords:** antiviral agents, CADD, DENV, NS5, sulfonamides

## Abstract

Emerging viral threats highlight the urgent need for antivirals targeting viral replication mechanisms. Based on previous research, we expanded a series of asymmetric benzene‐1,4‐disulfonamides. As these proved to be synthetically challenging and generally more toxic than expected, we optimized a hit structure by omitting one sulfonamide group. The resulting compound combines micromolar antiviral efficacy with low toxicity and straightforward synthesis. Molecular docking and enzyme‐linked immunosorbent assay (ELISA) assays confirmed that the compound targets the NS5 RNA polymerase domain of dengue virus‐2, blocking its interaction with NS3 and thereby inhibiting viral replication. A focused series of structural analogs further demonstrated the essential contribution of each individual part of the hit molecule.

## Graphical abstract

A synthetic methodology for the preparation of asymmetric benzene‐1,4‐disulfonamides was developed and applied to generate potential anti‐dengue virus (DENV) compounds. Guided by computer‐assisted structural rationalization, we designed synthetically more accessible analogs with reduced toxicity that interact with the DENV NS5 domain, thereby inhibiting viral replication

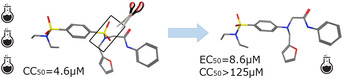

## 1 Introduction

Although several years have passed since the COVID‐19 pandemic, its impact still resonates with humanity. This costly lesson has clearly highlighted gaps in society's preparedness for novel and re‐emerging viruses, even though the factors facilitating further viral outbreaks are evident. Factors such as climate change, global travel, and urbanization create a favorable environment for viruses to spread beyond their original areas of occurrence. Of particular concern are orthoflaviviruses, that is, dengue virus (DENV), Zika virus (ZIKV), West Nile virus (WNV), and yellow fever virus, which are primarily transmitted by mosquitoes. Their vectors, including *Aedes aegypti* and *Aedes albopictus*, have recently expanded their geographical ranges due to global warming and environmental changes, such that their diffusion is no longer limited to tropical and subtropical regions [1, 2]. In this context, the development of antivirals that target steps in the viral life cycle common to several members of the *Flaviviridae* family is not only scientifically rational but also imperative from a public health perspective*.*

Despite the remarkable genetic diversity among orthoflaviviruses, the individual members share many structural elements in proteins that play a critical role in the replication cycle [3, 4, 5]. The conservation of the two essential viral enzymes and their mutual interaction create opportunities to inhibit a wide range of viruses with a single therapeutic agent.

**FIGURE 1:**
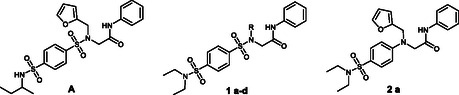
Structures of compound **A** [20], Series **1,** and **2a**.

Using this approach to target orthoflaviviruses, several anti‐flaviviral drugs targeting either the NS5 [6, 7, 8], the NS4B protein [9], the protease/helicase NS2B/NS3 [10, 11, 12], or their mutual interactions [13, 14, 15, 16, 17] have been reported. Within the viral replication machinery, the interaction of NS3 (helicase domain) and NS5 (RNA‐dependent RNA polymerase domain) has attracted considerable attention. Cannalire et al. demonstrated that the interaction of small molecules with NS5 can result in dual inhibition by blocking both NS3–NS5 complex formation and the NS5–RNA interaction [18]. Other dual‐acting inhibitors have also been reported; besides allosteric inhibition of NS5, these compounds inhibit host kinases that play an essential role in viral assembly [19]. Recently, a library of potential broad‐spectrum antivirals was screened in silico [20], with the aim of fitting a conserved binding pocket in the NS5 RNA polymerase domain and thereby blocking the interaction with NS3 helicase domain. Among the identified hits, one of the most promising compounds (Compound **A**, Figure 1) was found to have an effective concentration in the low micromolar range (EC_50_ = 1.67–5.74 μM) in blocking viral replication of the four DENV serotypes, as well as other flaviviruses such as WNV (EC_50_ = 2.10 μM) and ZIKV (EC_50_ = 1.66 μM). Within the concentration range tested, the compound resulted nontoxic in the uninfected cells.

This compound contains two sulfonamide groups in the 1,4‐positions of the aromatic ring, which makes its preparation laborious. The methods published to date are usually based on the sequential addition of amines to benzene‐1,4‐dichlorosulfonates [21]. These starting materials are very expensive as they are obtained either by the nucleophilic substitution of halogenated substrates [22] or through reactions of diazonium salts [23]. As the synthesis of benzene‐1,4‐dichlorosulfonates is itself challenging, the preparation of the final asymmetric benzene‐1,4‐disulfonamides from these materials is not guaranteed. Moreover, the sequential addition of different amines often leads to excessive formation of symmetrically substituted products, and purification of the reaction mixture introduces additional challenges.

Moreover, in structure **A**, the substitution of one sulfonamide is chiral. This feature introduces additional challenges for drug development based on this structure, while the calculated structure of the protein complex does not indicate any direct impact of this particular unit on binding.

In this work, we focused on developing an alternative synthetic route to asymmetric benzene‐1,4‐disulfonamides. Using a previously undescribed approach, we prepared a series of analogs of compound **A** (Series **1**) by omitting the chiral moiety and modifying the attached heterocycles. Furthermore, we proposed a rational design optimization of compound **A**, leading to compound **2a**. Finally, we described the synthesis, and we thoroughly investigated the impact of these new structural modifications on antiviral activity.

## 2 Results and Discussion

### 2.1 Series 1

Compound **A** demonstrated promising antiviral activity [20]. However, it represents a significant synthetic challenge, and the preparation of its structural analogs therefore becomes a very difficult task. For this reason, our main objective was to develop a synthetic strategy that would render asymmetric benzene‐1,4‐disulfonamides synthetically accessible.

**SCHEME 1:**
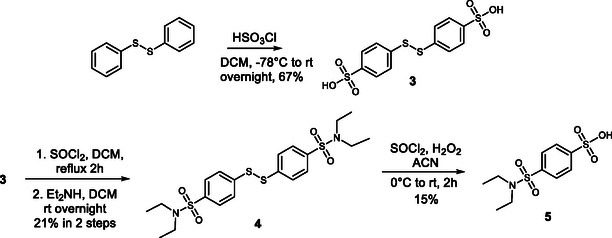
Synthesis of 4‐(*N*, *N*‐diethylsulfamoyl)benzene sulfonic acid **5** as a key intermediate for the synthesis of asymmetric benzene‐1,4‐disulfonamides.

The procedure presented here overcomes some of the drawbacks of previously published methods. As a starting material for chlorosulfonation, we selected diphenyl disulfide, in which the disulfide group acts as an electron‐donating moiety. Moreover, it provides steric hindrance at the ortho position, favoring *p*‐chlorosulfonation with 100% regioselectivity. The chlorosulfonation reaction was carried out in dichloromethane, and chlorosulfonic acid was added dropwise to diphenyl disulfide under cooling with a dry ice/ethanol bath. The solution changed color from clear to yellow. After complete addition, the reaction mixture was allowed to warm to room temperature (RT), resulting in a color change to deep green and precipitation of acid **3**. Acid **3** was isolated only once for characterization and yield determination; however, in the proposed synthetic pathway, its isolation was omitted as unnecessary. In the optimized synthesis, thionyl chloride was added directly to the green dichloromethane chlorosulfonation suspension, transforming **3** into the corresponding sulfonic acid chloride during a 2‐hour reflux. In the subsequent step, diethylamine was added, with the excess amine serving simultaneously as reagent and base, affording the symmetrical sulfonamide **4** in 21% overall yield over two steps. In this sulfonamide, the disulfide bond is oxidized by hydrogen peroxide and thionyl chloride to give 4‐(*N*, *N‐*diethylsulfamoyl)benzenesulfonic acid **5**. The oxidation step proved to be particularly challenging. According to the mechanism of disulfide oxidation under these conditions described by Bahrami [24], the reaction intermediates also contain free thiols. These can react with the resulting sulfonyl chloride, reforming the sulfur–sulfur bond and leading to excessive formation of side products. Product isolation is further complicated, as removal of excess hydrogen peroxide and water without aqueous work‐up is difficult. During aqueous work‐up, the chlorosulfonate partially transforms into the corresponding sulfonic acid, which exhibits problematic solubility in organic solvents. Considering these drawbacks, we performed the work‐up using large amounts of ethyl acetate as the extracting agent, followed by re‐activation of the partially hydrolyzed material through an additional treatment with thionyl chloride. After complete conversion to the sulfonic acid chloride, this compound is ready to react with various amines to afford asymmetric benzene‐1,4‐disulfonamide products (Scheme 1).

**SCHEME 2:**
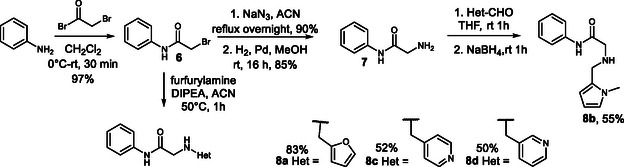
Synthesis of *N‐*phenyl glycinamide derivatives **8a–d**.

To obtain the final compounds, we needed to prepare amines containing different heterocycles bound to an *N‐*phenyl glycinamide moiety. The respective amines **8a–d** were prepared in advance, stored under an argon atmosphere, refrigerated, or preferably used immediately after preparation. Starting from aniline, alkylation with bromoacetyl bromide afforded *N*‐phenyl bromoacetamide **6** in quantitative yield. The reaction was exothermic and violent and therefore had to be performed carefully under cooling. A possible alternative is the use of chloroacetyl chloride as the reagent, which reacts more slowly; however, this method gives only an 88% yield. Nucleophilic substitution of bromine with azide, followed by reduction, afforded *N*‐phenyl glycinamide **7** in almost quantitative yields over both steps. Preparation of the amines was completed by reductive amination of the respective aldehydes with *N‐*phenyl glycinamide **7** (Scheme 2). In the case of **8a**, the product can also be obtained directly by alkylation of furfurylamine, followed by separation of the doubly alkylated side product.

**SCHEME 3:**
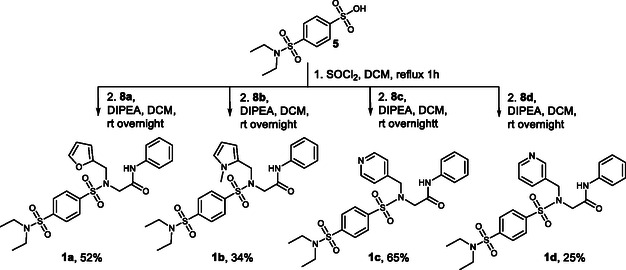
Synthesis of Series **1** of analogs of hit **A**.

In the final step, sulfonic acid **5** was completely converted into the corresponding sulfonyl chloride using thionyl chloride and then reacted with amines **8** dissolved in dichloromethane in the presence of diisopropylethylamine as a base. This method afforded the Series **1** compounds as analogs of compound **A** (Scheme 3).

### 2.2 Series 2

Due to the challenging synthesis of asymmetric benzene‐1,4‐disulfonamides, we attempted to simplify the molecule to improve synthetic accessibility while potentially retaining the antiviral activity of the original structure. The primary target of this simplification was removal of one sulfonamide group and the chiral center. Using molecular docking, we tested whether such modifications would prevent binding to the NS5 domain.

**FIGURE 2:**
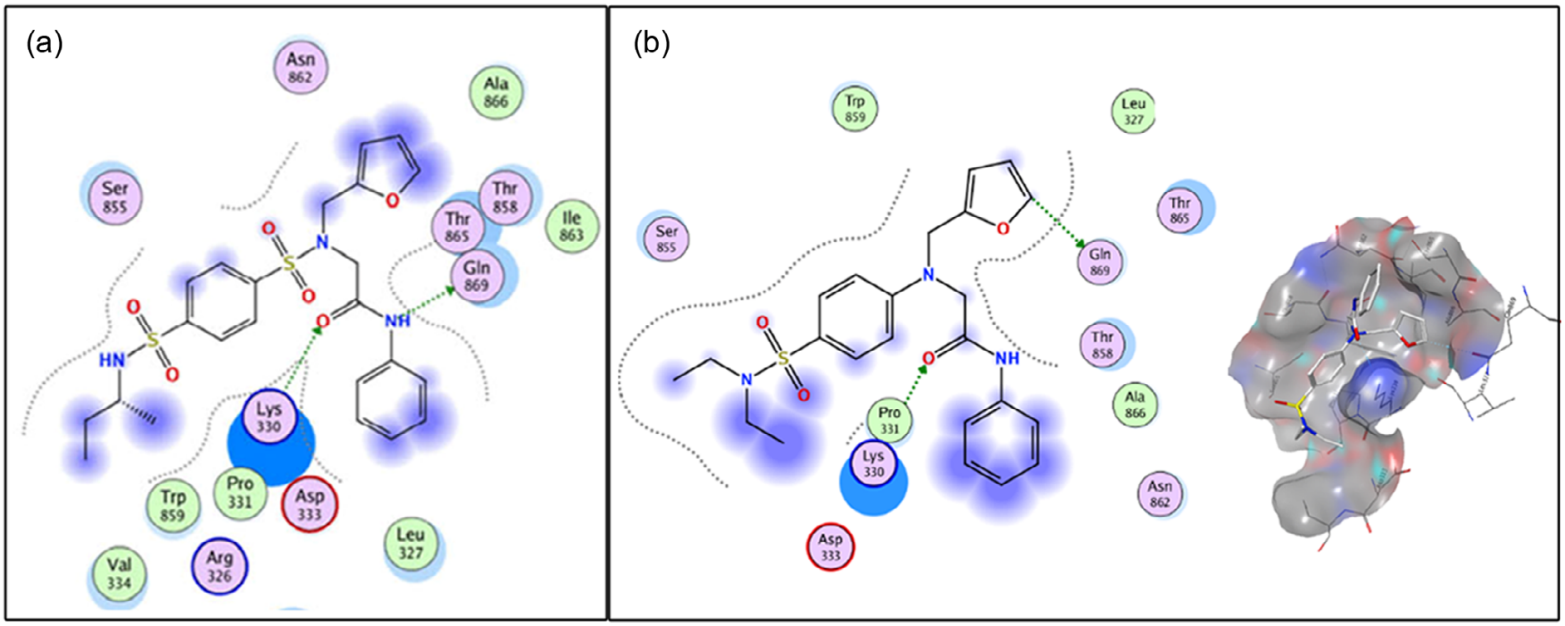
Molecular docking of **2a** into the binding pocket of DENV NS5: binding mode of compound **A** [20] (a) in comparison with **2a** (b).

Based on the reported molecular docking simulations, the strongest interaction between compound **A** and viral NS5 may arise between Lys330 and the amide oxygen of **A** (Figure 2a), with the NH of the same amide group interacting with Gln869 [20]. The bent shape of **A** is highly favorable or possibly necessary. In contrast, sulfonamide groups per se do not exhibit significant interactions with the protein nor does the chiral isobutyl substituent.

Based on this analysis, we optimized the structure of the hit compound by omitting one sulfonamide group, leading to structure **2a**. The bent molecular shape is preserved in compound **2a** through substitution at the aniline nitrogen, where the type of substituents was also retained. In contrast, the isobutyl substituent was replaced with achiral ethyl groups, thereby addressing two issues simultaneously: compound **2a** no longer contains a free sulfonamide proton, avoiding excessive sulfonamide acidity.

Molecular docking of compound **2a** into the NS5 binding cavity was performed (Figure 2b). According to the results, the positions of the substituents at the aniline nitrogen are switched compared to those in **A**. Nevertheless, the key interactions with Lys330 and Gln869 are preserved. In contrast to **A**, the furyl substituent of compound **2a** penetrates much deeper into the binding pocket, establishing a series of hydrophobic interactions with Gln869, Ala866, and Trp859, whereas the phenyl group of the 2‐oxo‐2‐(phenylamino)ethyl moiety is exposed to the environment outside the pocket. Replacement of the isobutyl group with two ethyl groups does not appear to alter significantly the binding pose.

**FIGURE 3:**
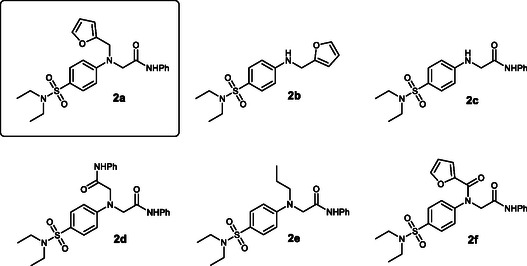
Compound **A** analogs of Series **2**.

To better understand the contribution of individual substituents to binding at the NS5 target, we also designed a number of alternative structures to clearly identify the key components of the hit (Figure 3).

Also, for these compounds, we performed molecular docking at the same binding site in NS5, yielding the following results. Compound **2b**, which lacks an amide function, binds to the NS5 binding site through a completely different mechanism, interacting with Thr865 instead of Gln869 (Figure S1), while preserving its position within the binding pocket. In all compounds containing an amide moiety (**2c**–**f**), the interaction with Lys330 is preserved.

**SCHEME 4:**
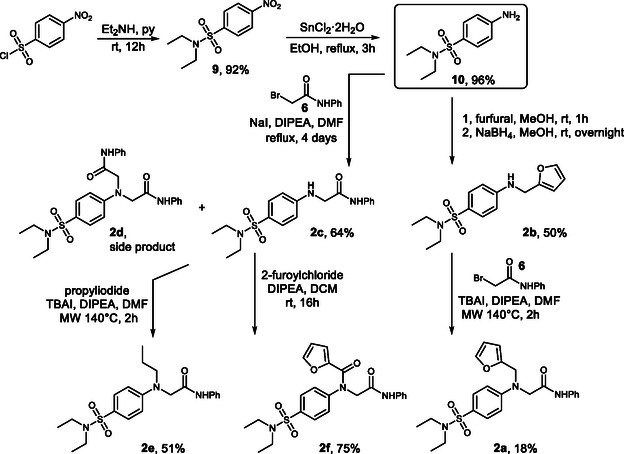
Synthesis of Series **2**.

In the case of **2c**, no additional significant interactions were observed (Figure S2). Compound **2d**, which contains two amide substituents, exhibits a complete change in the position of the sulfonamide moiety, which is displaced from the binding cavity (Figure S3). In compound **2e**, the propyl group does not introduce any new interactions (Figure S4). Introduction of an additional carbonyl group in compound **2f** again causes a change in the position of the sulfonamide within the cavity, as well as loss of interactions of the furan ring inside the pocket (Figure S5). Molecular Mechanics/Generalized Born Surface Area (MMGBSA) calculations performed on the docking results suggest that compound **2a** binds to NS5 with the highest affinity, whereas absence of any of the key molecular moieties leads to reduced binding affinity (Figure S6). To verify these hypotheses, we synthesized compound **2a** together with the entire library of proposed analogs (Scheme 4).

The attempt to obtain compound **2a** and its analogs focused on the precursor *p*‐aminophenyl‐*N*, *N*‐diethylsulfonamide **10**. This compound was readily accessible from commercially available nosyl chloride by reaction with diethylamine in pyridine, followed by SnCl_2_ reduction of the nitro group to the corresponding amine. When stored under an inert atmosphere, *p‐*aminophenyl‐*N*, *N*‐diethylsulfonamide **10** remained stable over long periods and therefore represents a versatile starting point for subsequent *N*’‐alkylation.

To prepare compound **2a**, we explored both possible derivatization routes of *p*‐aminophenyl‐*N*, *N*‐diethylsulfonamide **10**, namely initial introduction of either a furan‐2‐ylmethyl substituent or a 2‐oxo‐2‐(phenylamino)ethyl group. The furan‐2‐ylmethyl substituent was installed by reductive amination of *p*‐aminophenyl‐*N*, *N*‐diethylsulfonamide **10** with furfural, followed by reduction with sodium borohydride. As the Schiff base precipitated from the methanolic reaction mixture, the best results were obtained by isolating the precipitate and performing the reduction as a separate step. This method afforded compound **2b** in 50% yield.

The 2‐oxo‐2‐(phenylamino)ethyl substituent was subsequently introduced into **2b** by reaction with *N‐*phenyl‐2‐bromoacetamide **6**. However, the NH group of compound **2b** exhibits very low nucleophilicity; consequently, the reaction proceeded in very low yield, with prolonged heating leading to decomposition. These drawbacks were partially overcome by performing the reaction under microwave irradiation, affording compound **2a** in 18% yield.

Direct alkylation of *p*‐aminophenyl‐*N*, *N*‐diethylsulfonamide **10** with *N*‐phenyl‐2‐bromoacetamide **6** yielded both compounds **2c** and **2d** in 64% and 20% yield, respectively. The products were separated by flash chromatography. Attempts to introduce a furan‐2‐ylmethyl substituent into compound **2c** were unsuccessful, as the molecule was unreactive toward alkylation and harsher conditions led to decomposition of the furan‐containing starting materials. In contrast, compound **2c** was successfully alkylated with propyl iodide under microwave irradiation, affording compound **2e** in 51% yield. Compound **2c** also reacted readily with 2‐furoyl chloride to give compound **2f** in 75% yield.

**SCHEME 5:**
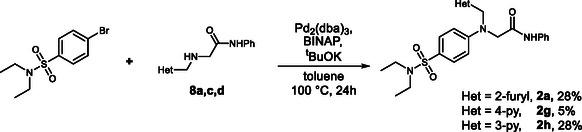
Alternative synthesis of **2a, 2g, 2h**.

To further broaden the scope of compounds prepared in Series **2**, we synthesized analogs of compounds **1c** and **1d**. Although the synthetic pathway described for the preparation of compound **2a** proved to be versatile, being based on inexpensive starting materials and providing several intermediates that could be further utilized for the synthesis of related analogs, we sought a more straightforward method. For this purpose, an alternative synthetic pathway was developed based on the Buchwald–Hartwig reaction (Scheme 5). In this procedure, amine **8** was reacted with 4‐bromo‐*N*, *N*‐diethylbenzene sulfonamide via Pd‐catalyzed coupling. This approach shortened the synthesis by several steps and, although the yields were modest, led directly to the target compounds. However, application of this method to obtain the Series **2** analog of compound **1b**, in which Het denotes an *N*‐methylpyrrole substituent, proved to be unsuccessful.

## 3 Biological Evaluation

To compare the antiviral activity of the prepared analogs with that of the published compound **A**, we first tested their ability to inhibit the replication of DENV‐2 by plaque reduction assays in Vero cells (Figure S7). The well‐established NS5 RdRP inhibitor NITD008 was included as a positive control. In parallel, the cytotoxicity was determined in uninfected Vero cells by MTT assays. As reported in Table 1, compound **1a** revealed a high cytotoxicity and thus was not further investigated. On the other hand, replacement of furan‐2‐yl by *N‐*methylpyrrol‐2‐yl in **1b** led to a less toxic compound, endowed with a selectivity Index (SI) = 4. Pyridine derivatives **1c** and **1d** proved to be toxic and not particularly active.

**TABLE 1:** Results of biological testing of Series **1**.

Compound	**Antiviral activity** **EC**_**50**_ a **(µM) (CI)** b	**Cytotoxicity** **CC**_**50**_ c **(µM)**	**SI** d
**1a**	ND	4.6 ± 1.8	—
**1b**	17.5 (8.8–35.5)	71.8 ± 6.9	4
**1c**	>25	16.6 ± 1.6	<1
**1d**	>25	23.1 ± 11.5	<1
**NITD008**	0.37 (0.02–5.28)	ND	—

a 50% effective concentration at half‐maximal response, i.e., the compound concentration that inhibits 50% of plaque formation, as determined by PRAs against DENV‐2 NGC strain in Vero cells.

b 95% confidence interval, as determined by nonlinear regression analysis with GraphPad Prism 10.0.

c 50% cytotoxic concentration that produces 50% of cytotoxicity, as determined by MTT assays in Vero cells. Reported values represent the mean ± SD of data derived from *n* = 3 independent experiments in duplicate.

d SI, selectivity index (determined as the ratio between CC_50_ and EC_50_).

ND, not determined.

In the case of Series **2**, biological testing clearly revealed that optimization of the hit structure maintained micromolar effective concentrations for compounds **2a** and **2b** (Table 2 and Figure S8). The toxicity of these compounds within the tested concentration range was negligible (Table 2). Removal of the amidic moiety in compound **2b** did not lead to loss of activity, whereas removal or replacement of the furan‐2‐yl moiety in compounds **2c**–**f** resulted in a dramatic loss of activity. Modifications in compounds **2c** and **2g** appear to confer some toxicity. Pyridyl derivatives **2g** and **2h** did not show appreciable activity against DENV, although compound **2h** was able to reduce plaque size (data not shown). This compound also precipitated in the cell culture medium during MTT assays at concentrations above 31.25 µM, which may affect accurate determination of its CC_50_.

**TABLE 2:** Results of biological testing of Series **2**.

Compound	**Antiviral activity** **EC**_**50**_ a **(µM) (CI)** b	**Cytotoxicity** **CC**_**50**_ c **(µM)**	**SI** d
**2a**	8.6 (2.7–23.7)	>125	>15
**2b**	14.1 (7.4–25.9)	>125	>8
**2c**	>25	67.1 ± 12.2	>3
**2d**	>25	>125	>5
**2e**	>25	>125	>5
**2f**	>25	>125	>5
**2g**	>25	56.3 ± 8.8	>2
**2h**	>25	>125	>5
**NITD008**	0.37 (0.02–5.28)	ND	—

a 50% effective concentration at half‐maximal response, i.e., the compound concentration that inhibits 50% of plaque formation, as determined by PRAs against DENV‐2 NGC strain in Vero cells. Reported values represent the mean ± SD of data derived from *n* ≥ 3 independent experiments in duplicate.

b 95% confidence interval, as determined by nonlinear regression analysis with GraphPad software 10.0.

c 50% cytotoxic concentration that produces 50% of cytotoxicity, as determined by MTT assays in Vero cells. Reported values represent the mean ± SD of data derived from *n* = 3 independent experiments in duplicate.

d SI, selectivity index (determined as the ratio between CC_50_ and EC_50_).

ND, not determined.

Finally, we performed NS3–NS5 enzyme‐linked immunosorbent assay (ELISA) interaction assays to further test the ability of the most promising analogs (i.e., those with SI > 5) to block the NS3–NS5 interaction in vitro. As reported in Table 3, compound **2a** was effective in interfering with the NS3–NS5 interaction, with an IC_50_ value approximately two‐fold lower than that of the original compound **A**.^18^ In contrast, for compound **2b**, the results of the ELISA assay suggest that it may act via a different mechanism, as no activity was observed (Table 3).

**TABLE 3:** Results of the NS3–NS5 ELISA interaction assay with **2a** and **2b**.

Compound	**NS3–NS5 ELISA** **IC**_**50**_ a **(µM)**
**2a**	75 ± 13
**2b**	>500

a Inhibitory concentration at half‐maximal response, i.e., the compound concentration that inhibits 50% of NS3–NS5 interaction in vitro, as determined by ELISA. Reported values represent the mean ± SD of data derived from *n* = 3 independent experiments in duplicate.

## 4 Conclusion

Preparation of asymmetrical benzene‐1,4‐disulfonamides, which represents a bottleneck in the synthesis of potentially valuable antiviral compounds, was achieved by developing a synthetic strategy relying on derivatization of diphenyl disulfide and subsequent oxidation of the S—S bond. Using this approach, we expanded the library of asymmetric benzene‐1,4‐disulfonamides, which unfortunately proved to be more toxic than expected. Computer‐assisted structural rationalization involving omission of one sulfonamide group led to the development of an optimized compound, **2a**, which retains micromolar efficacy and low toxicity while remaining synthetically accessible from inexpensive starting materials. Molecular docking studies, supported by ELISA assays, indicated that compound **2a** occupies the binding pocket of the DENV NS5 RdRp domain, thereby preventing its interaction with NS3 and subsequent viral RNA replication. In parallel with the hit compound, a small library of analogs was evaluated, clearly confirming the necessity of both substituents on the aniline moiety: the 2‐oxo‐2‐(phenylamino)ethyl group and the furan‐2‐ylmethyl group. The former is responsible for strong interactions with the protein, while the latter likely ensures correct positioning of the sulfonamide moiety, thereby enabling efficient blockade of the protein–protein interaction interface. As the NS5 structure is highly conserved across the *Flaviviridae* family, this novel compound represents an important step forward in the development of antiviral agents with a pan‐flaviviral spectrum, contributing to the fight against emerging and spreading flaviviruses.

## 5 Experimental Section

### 5.1 General

The chemicals and reagents were purchased from commercial sources and used without further purification. Anhydrous solvents were dried by PureSolv MD solvent purification system (Innovative Technology, Inc, USA). Analytical Thin Layer Chromatography (TLC) was carried out on foil sheets coated with silica gel containing a fluorescent indicator—60 F254 (Merck). The analyte was detected by UV light (wavelength 254 nm). Preparative TLC was carried out on 20 × 20 cm glass plates covered by silica gel 60 PF254 (Merck). The compounds were chromatographed using Pure C‐805 flash chromatography (Buchi Labortechnik, Switzerland). ^1^H nuclear magnetic resonance (NMR) spectra were measured by JEOL 400‐MHz (JEOL), and ^13^C spectra were measured by JEOL 400‐MHz (JEOL). Chemical shifts (δ) are presented in parts per million (ppm) referenced to the line of the solvent (δ/ppm; δ*H*/δC: DMSO‐*d*_6_, 2.50/39.52, CHCl_3_‐*d*, 7.26/77.16). To assign all proton and carbon signals, a combination of 1D and 2D experiments (H, C‐HSQC and H, C HMBC) was used. The high‐resolution mass spectra (HRMS) were measured on a MicrOtof III spectrometer (Bruker) with electrospray (ESI) or atmospheric pressure chemical ionization source (APCI) in positive mode. For calibration of accurate masses, ESI‐APCI Low Concentration Tuning Mix (Agilent) was used. The isotope profiles were calculated using freely available software EnviPat Web2.4 [25]. To perform microwave‐assisted reaction, a Discover 2.0 Microwave Synthesizer with 10 mL reaction vessels was used.

### 5.2 Molecular Modeling

The protein structure was downloaded from protein data bank (PDB: 5K5M) and was preprocessed using the Protein Preparation Workflow in Maestro (Schrödinger Release 2024‐3) [26]. Subsequently, energy minimization was performed using PRIME minimization applying the VSGB solvation model and the OPLS4 force field. The ligands were minimized using molecular operating environment (MOE2024) using the OPLS‐AA force field [27]. The ligprep function in Maestro was then used to generate the possible compound ionization states at pH 7±2 to secure proper geometry and parameters. The docking simulations were conducted in glide standard precision (SP) mode, extra precision (XP) mode, and Autodock Vina using the ligands prepared in ligprep. An enclosed cubic docking box with an edge length of 20 Å was centered on the binding site, which included amino acid residues 327, 330, 855, 859, 865, 866, and 869. A maximum of 10 poses per ligand were generated. After visual inspection, two representative poses for each ligand from each docking (SP, XP, and Autodock Vina) were selected, and MMGBSA calculations were performed using Prime, applying the VSGB solvation model and OPLS4 as a force field. MOE was used to visualize the results and generate the images.

### 5.3 Biological Evaluation

#### 5.3.1 Cells and Viruses

African Green Monkey Vero cells were purchased from American Type Culture Collection (VR‐CCL‐81) and were cultured in Dulbecco's Modified Eagle Medium (DMEM) supplemented with 10% fetal bovine serum (FBS, Life Technologies) in the presence of 100 U/mL penicillin and 100 μg/mL streptomycin (Life Technologies) and maintained at 37°C in a humidified atmosphere supplemented with 5% CO_2_. DENV‐2 (NGC strain) was purchased from NCPV (Public Health England, UK). All work with infectious DENV was performed in a biosafety level 3 laboratory according to the safety practices as approved by the Department of Molecular Medicine (University of Padua, Italy) Committee on Microbiological Safety.

#### 5.3.2 Plaque Reduction Assays

Plaque reduction assays were performed as previously described [20]. Briefly, Vero cells were seeded at 3 × 10^5^ cells/well in 12‐well plates and incubated for 24 h at 37°C. The next day, cells were washed and then infected with DENV‐2 NGC strain. Following 1 h of incubation at 37°C, cells were washed and then incubated with DMEM containing 2% FBS, 1.2% Avicel microcrystalline cellulose (FMC BioPolymer Philadelphia, PA, USA), and different concentrations of test compounds or 0.1% DMSO as a control. The NS5 inhibitor NITD008 (Merck) was added as a positive control. After 7 days of infection, cells were fixed, stained, and plaques were counted.

#### 5.3.3 Cytotoxicity Assays

The cytotoxicity of the test compounds was assessed in Vero cells by the 3‐(4,5‐dimethylthiazol‐2‐yl)‐2,5‐diphenyl tetrazolium bromide (MTT, Merck Life Science) method. Vero cells were cultured in 96‐well plates at a density of 1 × 10^4^ cells per well and incubated at 37°C overnight. The following day, cells were incubated with different concentrations of test compounds or DMSO for 72 h at 37°C. All the compound concentrations were tested at least in duplicate. Cell viability was then determined as previously reported [28].

#### 5.3.4 NS3–NS5 ELISA‐Based Assays

NS3–NS5 ELISA‐based assay to test the inhibition of the protein–protein interaction by test compounds was performed as previously reported [20]. Briefly, microtiter plates (96‐well, Nuova Aptaca) were coated with 300 ng of purified 6His‐NS3(177–618) for 3 h at 37°C and then blocked with 2% (wt/vol) BSA (Sigma) in phosphate‐buffered saline (PBS) for 2 h. After washes with PBS containing 0.3% Tween20, 200 ng of 6His‐NS5(272–900) was added and incubated O/N at 20°C in the absence or the presence of test compounds. After washing with PBS‐0.3% Tween20, samples were then incubated with anti‐NS5 antibody (ThermoFisher, diluted 1:3,000 in PBS containing 2% FBS) for 2 h at RT and successively with horseradish peroxidase‐conjugated goat anti‐mouse secondary antibody (Millipore; diluted 1:5,000 in PBS containing 2% FBS) for 2 h at RT. Following washes with PBS‐0.3% Tween20, the chromogenic substrate 3,3′, 5,5′ tetramethylbenzidine (KPL Inc.) was added and acidified with 3.6% HCl. Absorbances were read at 450 nm using a spectrophotometer microplate reader (Multiscan FC, ThermoFisher Scientific).

### 5.4 Synthesis

#### 5.4.1 4,4′‐Disulfanediyldibenzene sulfonic acid 3

Diphenyl disulfide (1 g, 4.5 mmol) was dissolved in dichloromethane and cooled to −78°C by ethanol/dry ice bath. After reaching the temperature, 5 equiv. of chlorosulfonic acid (1.52 mL, 22.5 mmol) were added. The reaction mixture was then left to reach the RT and stirred overnight, when it changed color to dark‐purple/green. The liquid part was separated and evaporated to dryness giving 4,4′‐disulfanediyldibenzene sulfonic acid **3** as a green solid in 67% yield.

^1^H NMR (400 MHz, DMSO‐*d*_6_) δ: 7.54 (d, *J* = 8.5 Hz, 4H), 7.44 (d, *J* = 8.5 Hz, 4H).

^13^C NMR (101 MHz, DMSO‐*d*_6_) δ: 148.1, 136.6, 127.2, 127.1.

HRMS APCI^+^: [C_12_H_10_O_6_S_4_+H]^+^ calc 378.9432, found 378.9433 [M+H]^+^.

#### 5.4.2 4,4′‐Disulfanediylbis(*N*, *N*‐diethylbenzene sulfonamide) 4

Diphenyl disulfide was dissolved in dichloromethane and cooled to −78°C by ethanol/dry ice bath. After reaching the temperature, 3 equiv. of chlorosulfonic acid were added. The reaction mixture was then left to reach the RT and stirred until it changed to dark‐purple/green. At this time, 3 equiv. of thionyl chloride were added, and the mixture was refluxed while stirring for 2 h. The solvents and surplus thionyl chloride were distilled off, and the residues were dried under lowered pressure for 1 h. Diethyl amine (10 equiv.) was diluted with dichloromethane and added to the sulfonic chloride immediately after drying. The reaction mixture was stirred at RT overnight. pH of the reaction was then adjusted to 2 by addition of 1 M HCl, and the product was extracted to dichloromethane. Organic phases were dried over MgSO_4_. After filtration, the solvents were evaporated, and the residue was purified by flash chromatography (ethyl acetate in cyclohexane 10%–100%). The product was obtained as a white solid in 21% yield after two steps.

^1^H NMR (400 MHz, CDCl_3_) δ: 7.72 (d, *J = *8.4 Hz, 4H), 7.54 (d, *J =* 8.4 Hz, 4H), 3.19 (q, *J =*  5.3 Hz, 8H), 1.10 (t, *J =*  5.2 Hz, 12H).

^13^C NMR (101 MHz, CDCl_3_) δ: 141.4, 139.3, 127.9, 126.6, 42.2, 14.3

HRMS ESI^+^: [C_20_H_28_N_2_O_4_S_4_ +H]^+^ calc 489.1004, found 489.1007, [M+H]^+^.

#### 5.4.3 4‐(*N*, *N‐*Diethylsulfamoyl)benzene sulfonic acid 5

4,4′‐Disulfanediylbis(*N*, *N*‐diethylbenzene sulfonamide) **4** (0.5 g, 1 mmol) was dissolved or dispersed in acetonitrile and cooled by an ice‐bath to 0°C. Into the stirred solution, hydrogen peroxide (30% in water, 0.7 mL, 6 equiv.) and thionyl chloride (1.5 mL, 20 equiv.) were added. The mixture was stirred for 2 h at RT. Acetonitrile was evaporated giving gummy slurry. The residue was taken up with ethyl acetate and washed with water (2 × 100 mL) and brine. The organic layer was dried over MgSO_4_ and after filtration evaporated to dryness. The products were used without further purification.

^1^H NMR (400 MHz, DMSO‐*d*_6_) δ: 7.72 (m, 4H), 3.09 (d, *J* = 7.2 Hz, 4H), 0.98 (t, *J* = 7.2 Hz, 6H).

^13^C NMR (101 MHz, DMSO‐*d*_6_) δ: 152.2, 140.0, 127.1, 127.0, 42.4, 14.6.

HRMS ESI^+^: [C_10_H_15_NO_5_S_2_ +H]^+^ calculated for 294.0465; found m/z: 294.0473 [M+H]^+^.

#### 5.4.4 Precursors for Compounds 8

##### 5.4.4.1 2‐Bromo‐N‐Phenylacetamide 6

Aniline (1 mL, 11 mmol) was dissolved in dichloromethane (DCM) (50 mL), and the solution was cooled to 0°C by an ice‐bath. Bromoacetyl bromide (0.5 mL, 5.5 mmol) was added dropwise. The solution was stirred until it reached the RT and then quenched by addition of water. The organic layer was separated and the water phase was washed with DCM (2 × 100 mL). Organic layers were combined and dried over MgSO_4_. After filtration, the evaporation gave pure product as a white solid in quantitative yield. ^1^H NMR spectra were in agreement with the literature [28].

^1^H NMR (400 MHz, CDCl_3_) δ: 8.23 (bs, 1H), 7.53 (dd, *J* = 8.3, 1.2 Hz, 2H), 7.36 (dd, *J* = 8.2, 7.5 Hz, 2H), 7.13 (t, *J* = 7.3 Hz, 1H), 4.03 (s, 2H).

##### 5.4.4.2 2‐Azido‐N‐Phenylacetamide 6a

2‐Bromo‐*N*‐phenylacetamide (0.5 g, 2.35 mmol) was dissolved in acetonitrile (50 mL). Sodium azide (0.15 g, 2.35 mmol) and sodium iodide (35 mg, 0.235 mmol) were added, and the reaction mixture was refluxed overnight. The reaction mixture was poured into water. The product was extracted to ethyl acetate (2 x100 mL); the organic phases were dried over MgSO_4_ and after filtration evaporated to dryness. The product was isolated as white solid in 90% yield. ^1^H NMR spectra were in agreement with the literature [29].

^1^H NMR (400 MHz, CDCl_3_) δ: 8.12 (brs, 1H), 7.54 (d, *J* = 8.6 Hz, 2H), 7.35 (dd, *J* = 8.4 Hz, *J* = 7.5 Hz, 2H), 7.16 (t, *J* = 8.6 Hz, 1H), 4.15 (s, 2H).

##### 5.4.4.3 2‐Amino‐N‐Phenylacetamide 7

2‐Azido‐*N*‐phenylacetamide (0.37 g, 21 mmol) was dissolved in methanol (50 mL) in an autoclave. Palladium (10% on carbon, 0.1 equiv.) was carefully added, the autoclave was filled by 8 bar of hydrogen gas, and the reaction mixture was stirred at RT for 16 h. The autoclave was opened and the reaction was filtered to remove Pd/C. After evaporation of methanol, the crude product was purified by flash chromatography (ethyl acetate in cyclohexane, 10%–100%). 2‐Amino‐N‐phenylacetamide was obtained as clear oil in 85% yield. ^1^H NMR spectra were in agreement with the literature [29].

^1^ *H NMR* (*CDCl*_3_, *400 MHz*) δ: *9.45* (*brs*, *1H*), *7.55* (*d*, *J = 8.0 Hz*, *2H*), *7.03* (*t*, *J = 7.2 Hz*, *1H*), *7.27* (*t*, *J = 7.6 Hz*, *2H*), *3.32* (*s*, *2H*), *1.69* (*brs*, *1H*)*.*

#### 5.4.5 General Procedure I

##### 5.4.5.1 Compounds 8

2‐Bromo‐*N*‐phenylacetamide (0.1 g, 0.47 mmol) was dissolved in acetonitrile (100 mL) and a corresponding amine (1 mol equiv.) was added together with Diisopropyl Ethyl Amine (DIPEA) (0.08 mL, 1 mol equiv.). The reaction mixture was stirred at 50°C for 1 h. The solvent was removed by evaporation under reduced pressure; the slurry was dissolved in DCM (100 mL) and washed with water (2 x 100 mL). The organic layer was dried over MgSO_4_ and after filtration evaporated to dryness.

##### 5.4.5.2 2‐((Furan‐2‐ylmethyl)amino)‐N‐Phenylacetamide 8a

The title compound was obtained according to the general procedure I using furfurylamine. The product was isolated by flash chromatography (ethyl acetate in cyclohexane 10%–100%), as a clear oil in 83% yield.

^1^H NMR (400 MHz, CDCl_3_) δ: 9.25 (s, 1H, NH), 7.56 (dd, *J* = 8.6, 1.6 Hz, 2H), 7.35 (dd, *J* = 1.6, 0.7 Hz, 1H), 7.31 (dd, *J* = 8.1, 7.4 Hz, 2H), 7.09 (t, *J* = 7.4 Hz, 1H), 6.30 (dd, *J* = 3.2, 1.7 Hz, 1H), 6.21 (dd, *J* = 3.2, 0.7 Hz, 1H), 3.82 (s, 2H), 3.38 (s, 2H), 2.01 (s, 1H, NH).

^13^C NMR (101 MHz, CDCl_3_) δ: 169.6, 152.6, 142.5, 137.7, 129.1, 124.2, 119.5, 110.4, 107.9, 52.0, 46.2.

HRMS ESI^+^: [C_13_H_14_N_2_O_2_+H]^+^ calc 231.1128, found 231.1131, [M+H]^+^.

##### 5.4.5.3 2‐(((1‐Methyl‐1H‐Pyrrol‐2‐yl)methyl)amino)‐N‐Phenylacetamide 8b

2‐Amino‐*N*‐phenylacetamide (0.3 g, 2 mmol) was dissolved in DCM (100 mL) and 1‐methyl‐1H‐pyrrole‐2‐carbaldehyde was added. The reaction mixture was stirred overnight at RT. The solvent was removed by evaporation at the reduced pressure, the slurry was suspended in methanol, NaBH_4_ (0.15 g, 4 mmol) was added, and the reaction mixture was stirred for 1 h. The reaction was quenched by the addition of water. The volatiles were removed under reduced pressure, and the water phase was extracted to DCM (3 × 200 mL). The combined organic layers were dried over MgSO_4_ and after filtration evaporated to dryness.

The product was isolated by flash chromatography (ethyl acetate in cyclohexane, 10%–100%) as yellowish solid in 55% yield.

^1^H NMR (400 MHz, CDCl_3_) δ: 9.15 (br s, 1H), 7.54 (dd, *J* = 8.3, 1.6 Hz, 2H), 7.33 (dd, *J* = 8.1, 7.3 Hz, 2H), 7.11 (t, *J* = 7.2 Hz, 1H), 6.63–6.60 (m, 1H), 6.12–6.07 (m, 2H), 3.81 (s, 2H), 3.67 (s, 3H), 3.45 (s, 2H), 1.74 (br s, 1H).

^13^C NMR (101 MHz, CDCl_3_) δ: 169.7, 137.7, 130.0, 129.2, 124.3, 123.0, 119.5, 108.6, 107.1, 52.6, 45.6, 33.9.

HRMS ESI^+^: [C_14_H_17_N_3_O+H]^+^ calc 244.1444, found 244.1447, [M+H]^+^.

##### 5.4.5.4 N‐Phenyl‐2‐((Pyridin‐4‐ylmethyl)amino)acetamide 8c

The title compound was obtained according to the general procedure I using pyridine‐4‐methylamine. The product was isolated by preparative TLC using mixture of methanol in DCM 20:1 as eluent (R_f_ 0.3), as yellowish oil in 50% yield.

^1^H NMR (400 MHz, CDCl_3_) δ: 9.09 (br s, 1H), 8.59 (d, *J* = 6.0 Hz, 2H), 7.54 (d, *J* = 7.3 Hz, 1H), 7.33 (t, *J* = 7.9 Hz, 2H), 7.28–7.25 (m, 2H), 7.12 (t, *J* = 7.4 Hz, 1H), 3.88 (s, 2H), 3.44 (s, 2H), 1.79 (br s, 1H).

^13^C NMR (101 MHz, CDCl_3_) δ: 169.1, 150.3, 148.0, 137.5, 129.2, 124.5, 122.9, 119.6, 52.8, 52.6.

HRMS ESI^+^: [C_14_H_15_N_3_O+H]^+^ calc 242.1288, found 242.1293, [M+H]^+^.

##### 5.4.5.5 N‐Phenyl‐2‐((pyridin‐3‐ylmethyl)amino)acetamide 8d

The title compound was obtained according to the general procedure I using pyridine‐3‐methylamine. The product was isolated by preparative TLC using mixture of methanol in DCM 20:1 as eluent (R_f_ 0.3), as yellowish oil in 52% yield.

^1^H NMR (400 MHz, CDCl_3_) δ: 9.14 (br s, 1H), 8.56 (d, *J* = 2.9 Hz, 1H), 8.49 (dd, *J* = 4.6, 2.9 Hz, 1H), 7.63–7.61 (m, 1H), 7.52 (dd, *J* = 8.8, 1.3 Hz, 2H), 7.30–7.26 (m, 2H), 7.25 – 7.23 (m, 1H), 7.07 (dt, *J* = 8.8, 1.3 Hz, 1H), 3.81 (s, 2H), 3.37 (s, 2H).

^13^C NMR (101 MHz, CDCl_3_) δ: 169.3, 149.7, 149.0, 137.5, 135.8, 134.5, 129.1, 124.3, 123.6, 119.5, 52.5, 51.4.

HRMS ESI^+^: [C_14_H_15_N_3_O+H]^+^ calc 242.1288, found 242.1292, [M+H]^+^.

#### 5.4.6 General Procedure II

##### 5.4.6.1 Series 1 Compounds

4‐(*N*, *N‐*Diethylsulfamoyl)benzene sulfonic acid **5** (100 mg, 0.26 mmol) was suspended in DCM (50 mL) and thionyl chloride (0.1 mL, 5 mol. equiv.) was added. The mixture was refluxed for 1 h, and the solvents were distilled off to dryness. The appropriate amine **8** (1 mol. equiv.) was dissolved in DCM together with DIPEA (0.15 mL, 3 mol. equiv.) and added to the slurry. The mixture was stirred overnight at RT. Water was added and the product was extracted into DCM (2 x 100 mL). The combined organic layers were dried over MgSO_4_ and after filtration evaporated to dryness.

##### 5.4.6.2 2‐((4‐(*N*,*N*‐Diethylsulfamoyl)‐*N′*‐(furan‐2‐ylmethyl)phenyl)sulfonamido)‐*N′*‐phenylacetamide 1a

The title compound was prepared according to general procedure II using amine **8a** as the starting material. The product was purified by flash chromatography (ethyl acetate in cyclohexane, 10%–100%) obtaining **1a** as an off‐white solid in 52% yield.

^1^H NMR (400 MHz, CDCl_3_) δ: 9.95 (s, 1H), 7.98 (d, *J* = 8.6 Hz, 2H), 7.90 (d, *J* = 8.6 Hz, 2H), 7.49 (dd, *J* = 1.8, 0.9 Hz, 1H), 7.42 (d, *J* = 7.9 Hz, 2H), 7.27 – 7.22 (m, 2H), 7.01 (t, *J* = 8.2 Hz, 1H), 6.32 (dd, *J* = 3.2, 1.8 Hz, 1H), 6.29 (dd, *J* = 3.3, 0.8 Hz, 1H), 4.55 (s, 2H), 3.99 (s, 2H), 3.11 (q, *J* = 7.3 Hz, 4H), 0.99 (t, *J* = 7.1 Hz, 6H).

^13^C NMR (101 MHz, DMSO‐*d*_6_) δ: 166.0, 149.2, 143.9, 143.8, 143.6, 138.9, 129.3, 128.6, 127.9, 124.0, 119.7, 111.1, 110.7, 49.7, 44.8, 42.4, 14.6.

HRMS ESI^+^: [C_23_H_27_N_3_O_6_S_2_+Na]^+^ calc 528.1233, found 528.1232, [M+Na]^+^.

##### 5.4.6.3 2‐((4‐(*N*,*N*‐Diethylsulfamoyl)‐*N′‐*((1‐methyl‐1H‐pyrrol‐2‐yl)methyl)phenyl)sulfonamido)‐*N*′‐phenylacetamide 1b

The title compound was prepared according to general procedure II using amine **8b** as the starting material. The product was purified by flash chromatography (ethyl acetate in cyclohexane, 10%–100%) obtaining **1b** as a yellow solid in 34% yield.

^1^H NMR (400 MHz, DMSO‐*D*_6_) δ: 7.98–7.96 (m, 4H), 7.50 (s, 1H), 7.30–7.26 (m, 4H), 7.09 (m, 1H), 6.53 (dd, *J* = 2.6, 1.8 Hz, 1H), 6.05 (dd, *J* = 3.5, 1.7 Hz, 1H), 5.99 (dd, *J* = 3.5, 2.7 Hz, 1H), 4.46 (s, 2H), 3.77 (s, 2H), 3.69 (s, 3H), 3.26 (q, *J* = 6.9 Hz, 4H), 1.14 (t, *J* = 7.0 Hz, 6H).

^13^C NMR (101 MHz, DMSO‐*D*_6_) δ: 165.4, 145.9, 141.1, 137.0, 129.0, 128.3, 128.0, 124.81, 124.80, 123.6, 120.0, 112.3, 107.8, 50.9, 45.6, 42.3, 34.0, 14.3.

HRMS ESI^+^: [C_24_H_30_N_4_O_5_S_2_+Na]^+^ calc 541.1550, found 541.1546, [M+Na]^+^.

##### 5.4.6.4 2‐((4‐(*N*,*N*‐Diethylsulfamoyl)‐*N*′‐(pyridin‐4‐ylmethyl)phenyl)sulfonamido)‐*N′‐*phenylacetamide 1c

The title compound was prepared according to general procedure II using amine **8c** as the starting material. The product was purified by flash chromatography (ethyl acetate in cyclohexane, 10%–100%), obtaining **1c** as an orange solid in 65% yield.

^1^H NMR (400 MHz, CDCl_3_) δ: 8.55 (d, *J* = 6.9 Hz, 2H), 8.08, (s, 1H), 8.06 (d, *J* = 8.9 Hz, 2H), 7.99 (d, *J* = 8.9 Hz, 2H), 7.33–7.26 (m, 6H), 7.13 (dd, *J* = 6.8, 2.5 Hz, 1H), 4.55 (s, 2H), 3.97 (s, 2H), 3.28 (q, *J* = 7.2 Hz, 4H), 1.17 (t, *J* = 7.2 Hz, 6H).

^13^C NMR (101 MHz, CDCl_3_) δ: 164.9, 150.3, 145.2, 144.0, 142.0, 136.8, 129.1, 128.2, 127.9, 125.0, 123.3, 119.8, 52.0, 51.1, 42.2, 14.2.

HRMS ESI^+^: [C_24_H_28_N_4_O_5_S_2_+H]^+^ calc 517.1574, found 517.1573, [M+H]^+^.

##### 5.4.6.5 2‐((4‐(*N*,*N*‐Diethylsulfamoyl)‐*N′*‐(pyridin‐3‐ylmethyl)phenyl)sulfonamido)‐*N′*‐phenylacetamide 1d

The title compound was prepared according to general procedure II using amine **8d** as the starting material. The product was purified by flash chromatography (ethyl acetate in cyclohexane, 10%–100%), obtaining **1d** as an orange solid in 25% yield.

^1^H NMR (400 MHz, CDCl_3_) δ: 8.58 (s, 1H), 8.54–8.53 (m, 1H), 8.04–7.97 (m, 5H), 7.74 (d, *J* = 7.7 Hz, 1H), 7.31–7.25 (m, 5H), 7.12 (dd, *J* = 6.8, 2.5 Hz, 1H), 4.57 (s, 2H), 3.93 (s, 2H), 3.29 (q, *J* = 7.2 Hz, 4H), 1.17 (t, *J* = 7.2 Hz, 6H).

^13^C NMR (101 MHz, CDCl_3_) δ: 165.1, 150.0, 145.2, 141.9, 136.7, 130.4, 129.0, 128.2, 127.9, 124.9, 123.9, 119.9, 51.0, 50.8, 42.2, 14.2.

HRMS ESI^+^[ C_24_H_28_N_4_O_5_S_2_+H]^+^ calc 517.1574, found 517.1571, [M+H]^+^.

##### 5.4.6.6 *N*,*N*‐Diethyl‐4‐nitrobenzene sulfonamide 9

4‐Nitrobenzene sulfonylchloride 0.5g (2.26 mmol) and diethylamine (0.233 mL, 2.26 mmol) were stirred in ice‐cold pyridine (10 mL). The mixture was stirred overnight. After reaction completion, 100 mL of water was added, and the pH was adjusted to 3 using concentrated HCl. The mixture was extracted to ethyl acetate (3 × 100 mL), and combined organic extracts were washed once with 50 mL of saturated sodium bicarbonate solution and once with 50 mL of brine. The organic phase was dried over MgSO_4_, filtered, and concentrated under vacuum. The product was isolated in 92% yield as a red solid. ^1^H NMR spectra were in agreement with the literature [30].

^1^H NMR (400 MHz, CDCl_3_) δ: 8.35 (d, *J* = 8.8 Hz, 2H), 8.00 (d, *J* = 8.8 Hz, 2H), 3.30 (q, *J* = 7.2 Hz, 4H), 1.16 (t, *J* = 7.2 Hz, 6H).

##### 5.4.6.7 *N*,*N*‐Diethyl‐4‐aminobenzene sulfonamide 10

*N*,*N*‐Diethyl‐4‐nitrobenzene sulfonamide (0.25 g, 0.9 mmol) was dissolved in ethanol (50 mL), and tin(II)chloride dihydrate (2.03 g, 9 mmol) was added. The solution was stirred at reflux for 16 h. The reaction progress was monitored by TLC. After reaction completion, excess KOH was added to adjust the pH to approximately 9. The formed precipitate was filtered using a fritted funnel and washed several times with ethanol until the resulting filtrate was clear. Filtrate was evaporated under reduced pressure, and subsequently, 50 mL of saturated sodium bicarbonate solution was added. The product was extracted to ethyl acetate (3 × 50 mL), and combined organic extracts were washed once with brine. The organic phase was dried over MgSO_4_, filtered, and concentrated under a vacuum. The product was isolated in 96% yield as an orange solid. ^1^H NMR spectra were in agreement with the literature [29].

^1^H NMR (400 MHz, CDCl_3_) δ: 7.57 (d, *J* = 8.6 Hz, 2H), 6.66 (d, *J* = 8.6 Hz, 2H), 3.18 (q, *J* = 7.2 Hz, 4H), 1.11 (t, *J* = 7.2 Hz, 6H).

##### 5.4.6.8 *N*,*N*‐Diethyl‐4‐((furan‐2‐ylmethyl)amino)benzene sulfonamide 2b

*N*,*N*‐Diethyl‐4‐aminobenzene sulfonamide (0.4 g, 1.75 mmol) was dissolved in methanol (50 mL) and furfural (0.14 mL, 1.75 mmol) was added. The white precipitate was separated by filtration. After drying, the precipitate was re‐dissolved in methanol (5 mL) and NaBH_4_ (0.1 g, 2.63 mmol) was added portion‐wise at vigorous stirring. The reaction was stirred overnight at RT. The reaction was quenched with 10 mL of water, and methanol was removed under vacuum. To the formed residue, saturated sodium bicarbonate solution was added to adjust pH to 9. Subsequently, the product was extracted to ethyl acetate (3 x 75 mL). Combined organic layers were washed with 30 mL of brine and dried over MgSO_4_. After filtration, the solution was concentrated under vacuum. The residue was purified by flash chromatography (ethyl acetate in cyclohexane, 10%–100%). The product was isolated in 50% yield as yellow oil.

^1^H NMR (400 MHz, CDCl_3_) δ: 7.57 (d, *J* = 8.8 Hz, 2H), 7.36 (dd, *J* = 1.8, 0.8 Hz, 1H), 6.52 (d, *J* = 8.8 Hz, 2H), 6.31 (dd, *J* = 3.2, 1.8 Hz, 1H), 6.24 (dd, *J* = 3.2, 0.8 Hz, 1H), 4.54 (s, 1H), 4.34 (d, *J* = 4.8 Hz, 2H), 3.18 (q, *J* = 7.1 Hz, 4H), 1.10 (t, *J* = 7.1 Hz, 6H).

^13^C NMR (101 MHz, CDCl_3_) δ: 151.5, 150.7, 142.4, 129.1, 128.0, 112.1, 110.5, 107.6, 42.0, 40.8, 14.3.HRMS ESI^+^: calc [C_15_H_19_N_2_O_3_S+H]^+^: 309.1267, found 309.1270 [M+H]^+^.

##### 5.4.6.9 2‐((4‐(*N*,*N*‐diethylsulfamoyl)phenyl)amino)‐*N′*‐phenylacetamide 2c and 2,2′‐((4‐(*N*,*N*‐diethylsulfamoyl)phenyl)azanediyl)bis(‐*N*′‐phenylacetamide) 2d

*N*,*N*‐diethyl 4‐aminobenzene sulfonamide (0.3 g, 1.31 mmol) was dissolved in DMF (10 mL), and NaI (0.05 g), 2‐bromo‐*N*‐phenylacetamide (0.31 g, 1.44 mmol) and DIPEA (0.231 mL) were added. Reaction mixture was heated to reflux over 4 days, and reaction rate was monitored using TLC. After cooling, the reaction mixture was poured to 50 mL of saturated sodium bicarbonate solution. The product was extracted to ethyl acetate (3 × 50 mL). The combined organic layers were washed with brine (50 mL), dried over MgSO_4_, filtered, and concentrated under vacuum. The residue was purified by flash chromatography (ethyl acetate in cyclohexane, 10%–100%). Compounds **2c** and **2d** were obtained in the approximate ratio of 12:1 as yellow solids, which were further recrystallized from mixture of 80% ethyl acetate in cyclohexane. Compound **2c** was isolated in 64%. Compound **2d** was isolated as a side‐product.

Data for **2c**:

^1^H NMR (400 MHz, CDCl_3_) δ: 8.23 (s, 1H), 7.62 (d, *J* = 4.9 Hz, 2H), 7.49 (d, *J* = 3.5 Hz, 2H), 7.31 (t, *J* = 7 Hz, 2H), 7.12 (t, *J* = 7.6 Hz, 1H), 6.66 (d, *J* = 8.0 Hz, 2H), 4.95 (t, *J* = 5.3 Hz, 1H), 3.94 (d, *J* = 2.9 Hz, 2H), 3.19 (q, *J* = 7.1, 1.1 Hz, 4H), 1.10 (t, *J* = 6.4 Hz, 6H).

^13^C NMR (101 MHz, CDCl_3_) δ: 167.6, 150.2, 137.1, 129.6, 129.28, 129.20, 124.9, 120.0, 112.8, 48.6, 42.1, 14.3.

HRMS ESI^+^: [C_18_H_23_N_3_O_3_S+H]^+^ calc 362.1531, found 362.1537, [M+H]^+^.

Data for **2d**:

^1^H NMR (400 MHz, CDCl_3_) δ: 9.66 (s, 2H), 7.66 (d, *J* = 8.8 Hz, 4H), 7.60 (d, *J* = 9.1 Hz, 2H), 7.34 (t, *J* = 8.1 Hz, 4H), 7.12 (t, J = 6.9 Hz, 2H), 6.59 (d, *J* = 9.1 Hz, 2H), 4.24 (s, 4H), 3.15 (q, *J* = 7.1 Hz, 4H), 1.10 (t, *J* = 7.2 Hz, 6H).

^13^C NMR (101 MHz, CDCl_3_) δ: 168.3, 149.5, 137.8, 129.20, 129.18, 128.9, 124.8, 120.0, 111.7, 58.7, 42.4, 14.5.HRMS ESI^+^: [C_26_H_30_N_4_O_4_S+H]^+^ calc 495.2080, found 495.2082, [M+H]^+^.

##### 5.4.6.10 2‐((4‐(*N*,*N*‐Diethylsulfamoyl)phenyl)(propyl)amino)‐*N′*‐phenylacetamide 2e

*N*,*N*‐Diethyl‐4‐aminobenzene sulfonamide (0.3 g, 0.83 mmol) was dissolved in DMF (1.5 mL), and propyl iodide (0.41 mL, 4.15 mmol) and DIPEA (0.16 mL 1.14 mmol) were added. The reaction mixture was transferred to a 7 mL microwave reaction vessel and heated in a microwave reactor to 140°C at 200 W for 2 h. The reaction mixture was poured into 50 mL of saturated sodium bicarbonate solution. The mixture was extracted to ethyl acetate (3 × 75 mL). The combined organic layers were washed with brine (50 mL), dried over MgSO_4_, filtered, and concentrated under vacuum. The residue was purified by flash chromatography (ethyl acetate in cyclohexane, 10%–100%). Compound **2e** was obtained as an off‐white solid, which was further recrystallized from ethyl acetate. The isolated yield was 51%.

^1^H NMR (400 MHz, CDCl_3_) δ: 7.87 (s, 1H), 7.67 (d, *J* = 9.0 Hz, 2H), 7.43 (d, *J* = 8.5 Hz, 2H), 7.30 (t, *J* = 7.9 Hz, 2H), 7.12 (t, *J* = 6.3 Hz, 1H), 6.75 (d, *J* = 9.0 Hz, 2H), 4.05 (s, 2H), 3.46 (t, *J* = 8.5 Hz, 2H), 3.18 (q, *J* = 7.1 Hz, 4H), 1.77–1.66 (m, 2H), 1.11 (t, *J* = 7.1 Hz, 6H), 1.00 (t, *J* = 7.4 Hz, 3H).^13^C NMR (101 MHz, CDCl_3_) δ: 167.5, 150.4, 136.8, 129.5, 129.3, 129.2, 125.0, 120.1, 112.3, 56.8, 54.1, 42.1, 20.0, 14.3, 11.4.HRMS ESI^+^: [C_21_H_29_N_3_O_3_S+H]^+^ calc 404.2002, found 404.2009, [M+H]^+^.

##### 5.4.6.11 *N*‐(4‐(*N’*,*N′*‐Diethylsulfamoyl)phenyl)‐*N*‐(2‐oxo‐2‐(phenylamino)ethyl)furan‐2‐carboxamide 2f

*N*,*N*‐Diethyl 4‐aminobenzene sulfonamide (0.3 g, 0.83 mmol) was dissolved in DCM (20 mL) and DIPEA (0.16 mL, 0.91 mmol) was added. Then, 2‐furoyl chloride (0.09 mL, 0.91 mmol) was carefully introduced, and the mixture was stirred overnight. After addition of water (50 mL), the mixture was extracted to ethyl acetate (2 x 50 mL). The combined organic layers were dried over MgSO_4_, filtered, and concentrated under vacuum. Recrystallization from 1:1 mixture of boiling ethyl acetate: hexane gave the product in 75% yield as white needle‐shaped crystals.

^1^H NMR(400 MHz, CDCl_3_) δ: 8.64 (s, 1H), 7.83 (d, *J* = 8.6 Hz, 2H), 7.49 (d, *J* = 7.9 Hz, 2H), 7.41 (d, *J* = 6.6 Hz, 2H), 7.28–7.23 (m, 3H), 7.08 (t, *J* = 7.3 Hz, 1H), 6.29 (d, *J* = 2.1 Hz, 2H), 4.59 (d, *J* = 1.9 Hz, 2H), 3.26 (q, *J* = 7.1 Hz, 4H), 1.14 (t, *J* = 7.1 Hz, 6H).

^13^C NMR (101 MHz, CDCl_3_) δ: 166.2, 160.0, 146.2, 145.9, 145.5, 140.3, 137.6, 129.1, 128.6, 128.4, 124.6, 120.0, 118.9, 111.7, 56.0, 42.2, 14.3.

HRMS ESI^+^: calc [C_23_H_25_N_33_O_5_S+H]^+^: 456.1587, found 456.1587.

##### 5.4.6.12 2‐((4‐(*N*,*N*‐Diethylsulfamoyl)phenyl)(furan‐2‐ylmethyl)amino)‐*N′*‐phenylacetamide 2a, from Compound 2b

*N*,*N*‐Diethyl‐4‐((furan‐2‐ylmethyl)amino)benzene sulfonamide **2b** (0.2 g, 0.64 mmol) was dissolved in DMF (1.5 mL) and transferred to 7 mL microwave reaction vessel. 2‐Bromo‐*N*‐phenylacetamide (0.15 g, 0.71 mmol), DIPEA (0.13 mL, 0.71 mmol), and Tetrabutyl ammonium iodide (TBAI) (0.05 g) were added. The reactor was sealed and heated in a microwave reactor to 140°C at 200 W for 2 h. After completion, the reaction mixture was poured into 50 mL of saturated sodium bicarbonate solution. The mixture was extracted to ethyl acetate (3 × 75 mL). The combined organic layers were washed with brine (50 mL), dried over MgSO_4_, filtered, and concentrated under vacuum. The residue was purified by flash chromatography (ethyl acetate in cyclohexane, 10%–100%). Fractions containing product were concentrated under reduced pressure and further purified using preparative TLC with a mixture of DCM: MeOH (20:1, 0.1% TEA). The separated product was re‐dissolved in 2.5 mL of dry THF and precipitated using excess HCl/Et_2_O solution as hydrochloride salt. After filtration, the precipitate was washed with small amount of Et_2_O. The product was isolated in 18% yield as off‐white solid.

^1^H NMR (400 MHz, CDCl_3_) δ: 8.19 (s, 1H), 7.65 (d, *J* = 9.0 Hz, 2H), 7.49 – 7.36 (m, 3H), 7.29 (t, *J* = 7.9 Hz, 2H), 7.10 (t, *J* = 7.4 Hz, 1H), 6.90 (d, *J* = 9.0 Hz, 2H), 6.45–6.31 (m, 2H), 4.65 (s, 2H), 4.14 (s, 2H), 3.18 (q, *J* = 7.1 Hz, 4H), 1.10 (t, *J* = 7.1 Hz, 6H).^13^C NMR (101 MHz, CDCl_3_) δ: 167.5, 150.6, 150.1, 143.0, 137.1, 130.1, 129.17, 129.12, 124.8, 119.9, 113.0, 111.0, 109.3, 57.6, 49.5, 42.1, 14.4.

HRMS ESI^+^: [C_23_H_27_N_3_O_3_S+H]^+^ calc 442.1795, found 442.1789, [M+H]^+^.

#### 5.4.7 General Procedure III

*N*,*N*‐Diethyl 4‐bromobenzene sulfonamide (0.1 g, 0.34 mmol), amine **8** (1 mol. equiv.), Pd_2_(dba)_3_ (0.012 g, 0.014 mmol), BINAP (0.042 g, 0.078 mmol), and *t*‐BuOK (0.14 g, 1.25 mmol) were inserted into a Schlenk flask. The flask was connected to a vacuum line, evacuated, and filled with argon atmosphere. The procedure was repeated three times. Through a rubber septum, anhydrous toluene (10 mL) was injected, and the mixture was stirred at 100°C for 24 h. After reaction completion, saturated sodium bicarbonate (50 mL) was added, and the toluene layer was separated. The water phase was extracted with ethyl acetate (2–×–50 mL). Combined organic layers were washed with brine (50 mL), dried over MgSO_4_, filtered, and concentrated under vacuum.

##### 5.4.7.1 2‐((4‐(*N*,*N*‐diethylsulfamoyl)phenyl)(furan‐2‐ylmethyl)amino)‐*N′*‐phenylacetamide 2a, alternative

The compound was prepared using amine **8a** as the starting material. After drying, the residue was purified by flash chromatography (ethyl acetate in cyclohexane, 20%–100%). The product was obtained as a pale‐yellow solid in 28% yield. Spectroscopic data: see Section 5.4.6.12.

##### 5.4.7.2 *N*,*N*‐diethyl‐4‐((2‐oxo‐2‐phenylethyl)(pyridin‐4‐ylmethyl)amino)benzene sulfonamide 2g

The compound was prepared using amine **8c** as the starting material. After drying, the residue was purified by preparative TLC using mixture of methanol in DCM 20:1 as eluent (R_f_ 0.65). The product was obtained as a pale‐yellow solid in 5% yield.

^1^H NMR (400 MHz, CDCl_3_) δ: 8.57–8.55 (m, 2H), 8.07 (s, 1H), 7.62 (d, *J* = 8.6 Hz, 2H), 7.46 (d, *J* = 8.0 Hz, 2H), 7.30 (t, *J* = 7.9 Hz, 2H), 7.14–7.11 (m, 3H), 6.72 (d, *J* = 8.5 Hz, 2H), 4.76 (s, 2H), 4.16 (s, 2H), 3.16 (q, *J* = 7.1 Hz, 4H), 1.11 (t, *J* = 7.2 Hz, 6H).

^13^C NMR (101 MHz, CDCl_3_) δ: 166.9, 150.5, 150.5, 145.8, 137.0, 129.8, 129.3, 129.2, 125.1, 121.7, 120.2, 112.5, 56.3, 54.7, 42.2, 14.4.

HRMS ESI^+^: [C_24_H_28_N_4_O_3_S+H]^+^ calc 453.1956, found 453.1962, [M+H]^+^.

*N*,*N*‐diethyl‐4‐((2‐oxo‐2‐phenylethyl)(pyridin‐3‐ylmethyl)amino)benzene sulfonamide **2h**

The compound was prepared using amine **8d** as the starting material. After drying, the residue was purified by preparative TLC using mixture of methanol in DCM 20:1 as eluent (R_f_ 0.65). The product was obtained as a pale‐yellow solid in 28% yield.

^1^H NMR (400 MHz, CDCl_3_) δ: 8.55 (d, *J* = 4.8 Hz, 1H), 8.50 (s, 1H), 8.20 (s, 1H), 7.60 (d, *J* = 8.9 Hz, 2H), 7.54 (d, *J* = 7.9 Hz, 1H), 7.45 (d, *J* = 8.1 Hz, 2H), 7.31–7.27 (m, 3H), 7.12 (t, *J* = 7.6 Hz, 1H), 6.75 (d, *J* = 8.8 Hz, 2H), 4.79 (s, 2H), 4.13 (s, 2H), 3.17 (q, *J* = 7.1 Hz, 4H), 1.10 (t, *J* = 7.1 Hz, 6H).^13^C NMR (101 MHz, CDCl_3_) δ: 167.1, 150.7, 149.3, 148.5, 137.1, 134.7, 132.1, 129.4, 129.2, 129.1, 125.0, 123.9, 120.2, 112.6, 56.0, 53.3, 42.2, 14.4.

HRMS ESI^+^: [C_24_H_28_N_4_O_3_S+H]^+^ calc 453.1956, found 453.1952, [M+H]^+^.

## Author Contributions

**Jakub Janáč** and **Jan Drahorád** synthesis (equal). **Jakub Harvalík** computing. **Tereza Horáčková** and **Manfred Brinker** synthesis (supporting). **Arianna Loregian** and **Andrea Brancale** funding acquisition, project administration, review and editing (supporting). **Beatrice Mercorelli** and **Petra Cuřínová** conceptualization, supervision, methodology, investigation, writing original draft (lead, equal).

## Funding

This work was supported by the New Technologies for Translational Research in Pharmaceutical Sciences/NETPHARM (CZ.02.01.01/00/22_008/0004607), Ministero dell’Università e della Ricerca (MIUR), Italy (20223RYYFC, P20222YKP8), NextGenerationEU‐MUR PNRR Extended Partnership initiative on Emerging Infectious Diseases (PE00000007), Associazione Italiana per la Ricerca sul Cancro (2021‐ID. 25899), Fondazione Cassa di Risparmio di Padova e Rovigo (55777 2020.0162‐ARREST‐COV), and University of Padua, Italy (PRID2021).

## Conflicts of Interest

The authors declare no conflicts of interest.

## Supporting information

Supplementary Material

## Data Availability

The data supporting this research are available as a supporting information.

## References

[1] J. Hu , B. P. Horton , T. W. Yeo , J. J. Y. Sung , and Y. H. L. Steve , “Mosquito and Global Dengue Cases in a Warming World,” BMJ Global Health 10 (2025), 10.1136/bmjgh-2023-014688. PMC12056631 40335075

[2] E. Abbasi , “The Impact of Climate Change on Travel‐Related Vector‐Borne Diseases: A Case Study on Dengue Virus Transmission,” Travel Medicine and Infectious Disease 65 (2025): 102841, 10.1016/j.tmaid.2025.102841. 40118163

[3] M. A. Feinberg , M. T. Le , K. L. Carpio , E. Knyazhanskaya , A. D. T. Barrett , and K. H. Choi , “Interaction of West Nile Virus NS5 With Orthoflavivirus SLA RNAs and Their Effects on Viral Replication and Inhibition,” Journal of Virology 99 (2025), 10.1128/jvi.02023-24. PMC12455937 40824093

[4] M. Z. Palo , B. Ha , C. P. Lapointe , et al., “Conserved Long‐Range Interactions Are Required for Stable Folding of Orthoflaviviral Genomic RNA,” Nucleic Acids Research 53 (2025), 10.1093/nar/gkaf514. PMC12168079 40521663

[5] S. Sornprasert , W. Sornjai , and D. R. Smith , “The Interaction of Orthoflavivirus Nonstructural Proteins 3 and 5 With Human Fatty Acid Synthase,” PLoS ONE 20 (2025), 10.1371/journal.pone.0319207. PMC11936160 40131913

[6] S. P. Lim , C. G. Noble , C. C. Seh , et al., “Potent Allosteric Dengue Virus NS5 Polymerase Inhibitors: Mechanism of Action and Resistance Profiling,” PLoS Pathogens 12 (2016), 10.1371/journal.ppat.1005737. PMC4976923 27500641

[7] F. Benmansour , I. Trist , B. Coutard , et al., “Discovery of Novel Dengue Virus NS5 Methyltransferase Non‐Nucleoside Inhibitors by Fragment‐Based Drug Design,” European Journal of Medicinal Chemistry 125 (2017): 865–880, 10.1016/j.ejmech.2016.10.007. 27750202

[8] R. Cannalire , D. Tarantino , A. Astolfi , et al., “Functionalized 2,1‐Benzothiazine 2,2‐Dioxides as New Inhibitors of Dengue NS5 RNA‐Dependent RNA Polymerase,” European Journal of Medicinal Chemistry 143 (2018): 1667–1676, 10.1016/j.ejmech.2017.10.064. 29137867

[9] S. A. Moquin , O. Simon , R. Karuna , et al., “NITD‐688, a Pan‐Serotype Inhibitor of the Dengue Virus NS4B Protein, Shows Favorable Pharmacokinetics and Efficacy in Preclinical Animal Models,” Science Translational Medicine 13 (2021), 10.1126/scitranslmed.abb2181. 33536278

[10] Z. Li , M. Brecher , Y.‐Q. Deng , et al., “Existing Drugs as Broad‐Spectrum and Potent Inhibitors for Zika Virus by Targeting NS2B–NS3 Interaction,” Cell Research 27 (2017), 10.1038/cr.2017.88. PMC5539352 28685770

[11] S. A. Shiryaev , C. Farhy , A. Pinto , et al., “Characterization of the Zika Virus Two‐Component NS2B–NS3 Protease and Structure‐Assisted Identification of Allosteric Small‐Molecule Antagonists,” Antiviral Research 143 (2017), 10.1016/j.antiviral.2017.04.015. PMC5558795 28461069

[12] Z. Li , S. Sakamuru , R. Huang , et al., “Erythrosin B Is a Potent and Broad‐Spectrum Orthosteric Inhibitor of the Flavivirus NS2B–NS3 Protease,” Antiviral Research 150 (2018), 10.1016/j.antiviral.2017.12.018. PMC5892443 29288700

[13] C. Basavannacharya and S. G. Vasudevan , “Suramin Inhibits Helicase Activity of NS3 Protein of Dengue Virus in a Fluorescence‐Based High‐Throughput Assay Format,” Biochemical and Biophysical Research Communications 453 (2014), 10.1016/j.bbrc.2014.09.113. 25281902

[14] A. Fikatas , P. Vervaeke , E. Meyen , et al., “A Novel Series of Indole Alkaloid Derivatives Inhibit Dengue and Zika Virus Infection by Interference With the Viral Replication Complex,” Antimicrobial Agents and Chemotherapy 65 (2021), 10.1128/AAC.02349-20. PMC8284442 34001508

[15] S. Pambudi , N. Kawashita , S. Phanthanawiboon , et al., “A Small Compound Targeting the Interaction Between Nonstructural Proteins 2B and 3 Inhibits Dengue Virus Replication,” Biochemical and Biophysical Research Communications 440 (2013), 10.1016/j.bbrc.2013.09.078. 24070610

[16] S. J. F. Kaptein , O. Goethals , D. Kiemel , et al., “A Pan‐Serotype Dengue Virus Inhibitor Targeting the NS3–NS4B Interaction,” Nature 598 (2021), 10.1038/s41586-021-03990-6. 34671169

[17] G. Nannetti , B. Mercorelli , A. Bazzacco , et al., “New Dengue Virus Inhibitors Targeting NS3–NS5 Interaction Identified by in Silico Screening,” Frontiers in Microbiology 16 (2025), 10.3389/fmicb.2025.1663404. PMC12615485 41244686

[18] R. Cannalire , K. Wing Ki Chan , M. Sole Burali , et al., “Pyridobenzothiazolones Exert Potent Anti‐Dengue Activity by Hampering Multiple Functions of NS5 Polymerase“,” ACS Medicinal Chemistry Letters 11 (2020): 773–782. 32435384 10.1021/acsmedchemlett.9b00619 PMC7236247

[19] P. Vincetti , F. Caporuscio , S. Kaptein , et al., “Discovery of Multitarget Antivirals Acting on Both the Dengue Virus NS5‐NS3 Interaction and the Host Src/Fyn Kinases,” Journal of Medicinal Chemistry 58 (2015): 4964–4975. 26039671 10.1021/acs.jmedchem.5b00108

[20] M. Celegato , M. Sturlese , V. V. Costa , et al., “Small‐Molecule Inhibitor of Flaviviral NS3–NS5 Interaction with Broad‐Spectrum Activity and Efficacy In Vivo,“ mBio 14 (2023), 10.1128/mbio.03097-22. PMC9973282 36622141

[21] D. F. Hayman , V. Petrow , O. Stephenson , and A. J. Thomas , “Hypoglycaemic Agents. Part II,” Journal of Pharmacy and Pharmacology 14 (1962), 10.1111/j.2042-7158.1962.tb11123.x. 13905665

[22] E. J. Emmett , B. R. Hayter , and M. C. Willis , “Palladium‐Catalyzed Three‐Component Diaryl Sulfone Synthesis Exploiting the Sulfur Dioxide Surrogate DABSO,” Angewandte Chemie 125 (2013), 10.1002/ange.201305369. PMC4138993 24115325

[23] R. V. Hoffman , “m‐Trifluoromethylbenzenesulphonyl Chloride,” Organic Syntheses 60 (1981), 10.15227/orgsyn.060.0121.

[24] K. Bahrami , M. M. Khodaei , and M. Soheilizad , “Direct Conversion of Thiols to Sulfonyl Chlorides and Sulfonamides,” The Journal of Organic Chemistry 74 (2009), 10.1021/jo901924m. 19919028

[25] M. Loos , C. Gerber , F. Corona , J. Hollender , and H. Singer , “Accelerated Isotope Fine Structure Calculation Using Pruned Transition Trees,” Analytical Chemistry 87 (2015), 10.1021/acs.analchem.5b00941. 25929282

[26] Schrödinger , “Maestro,” Schrödinger LLC, 2019, accessed January 5, 2026, https://www.schrodinger.com/maestro.

[27] Chemical Computing Group ULC , “Molecular Operating Environment (MOE),“ Chemical Computing Group ULC, 2024, accessed January 5, 2026, https://www.chemcomp.com/Products.htm.

[28] J. Feng , T. Gao , F. Morlet‐Savary , et al., “Sunlight‐Driven Photoinitiating Systems for Photopolymerization and Application in Direct Laser Writing,” Polymer Chemistry 15 (2024), 10.1039/d4py00558a.

[29] X. Chen , J. Wang , J. Cui , Z. Xu , and X. Peng , “A Ratiometric and Exclusively Selective Cell Fluorescent Probe Based on Internal Charge Transfer,” Tetrahedron 67 (2011), 10.1016/j.tet.2011.05.001.

[30] A. B. Pinkerton , E. H. Sessions , P. Hershberger , et al., “Optimization of a Urea‐Containing Series of Nicotinamide Phosphoribosyltransferase (NAMPT) Activators,” Bioorganic & Medicinal Chemistry Letters 48 (2021), 10.1016/j.bmcl.2021.128007. 33798699

