# Supplementary material for: Synthesis and Evaluation of Aromatic Sulfonamide and Disulfonamide Derivatives as Dengue Virus NS3–NS5 Interaction Inhibitors
Source: ChemMedChem. 2026 Jun 5;21(11):e70337. doi: 10.1002/cmdc.70337 (PMC13241294; doi:10.1002/cmdc.70337)
Supplement: Supplementary file 1 — Supplementary Material [file CMDC-21-e70337-s001.pdf]

Supporting information for:

Synthesis and evaluation of aromatic sulfonamides and disulfonamides derivatives as dengue virus NS3-NS5 interaction inhibitors.

Janáč Jakub [a], Jan Drahorád [a], Harvalík Jakub [a], Tereza Horáčková [a], Manfred Brinker [a], Arianna Loregian [b,c], Andrea Brancale [a], Beatrice Mercorelli* [b], Petra Cuřínová* [a]

[a] *Department of Organic Chemistry, University of Chemistry and Technology in Prague, Prague, Czech Republic*

[b] *Department of Molecular Medicine, University of Padua, Padua, Italy*

[c] *Microbiology and Virology Unit, Padua University Hospital, Padua, Italy*

Content

1. Spectral characterization of the compounds: 2
2. Molecular docking 30
3. Results of biological testing 33

1. Spectral characterization of the compounds:

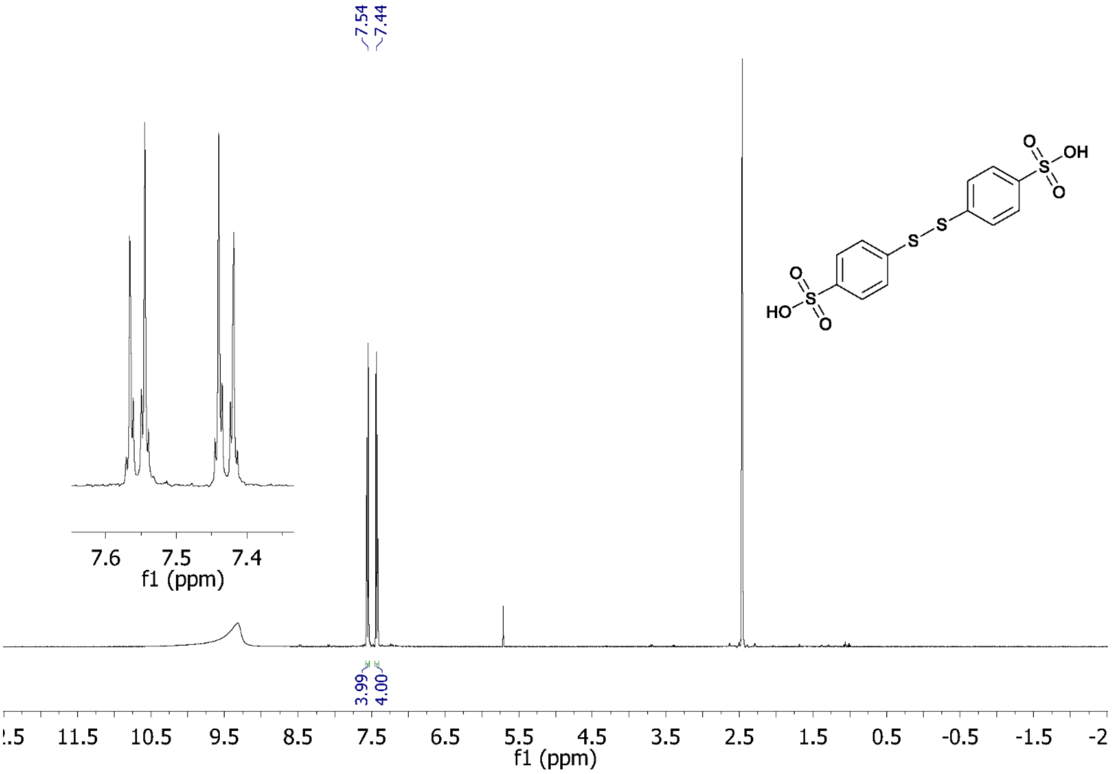

Compound **3**, ^{1}H NMR (400 MHz, DMSO-d_6).

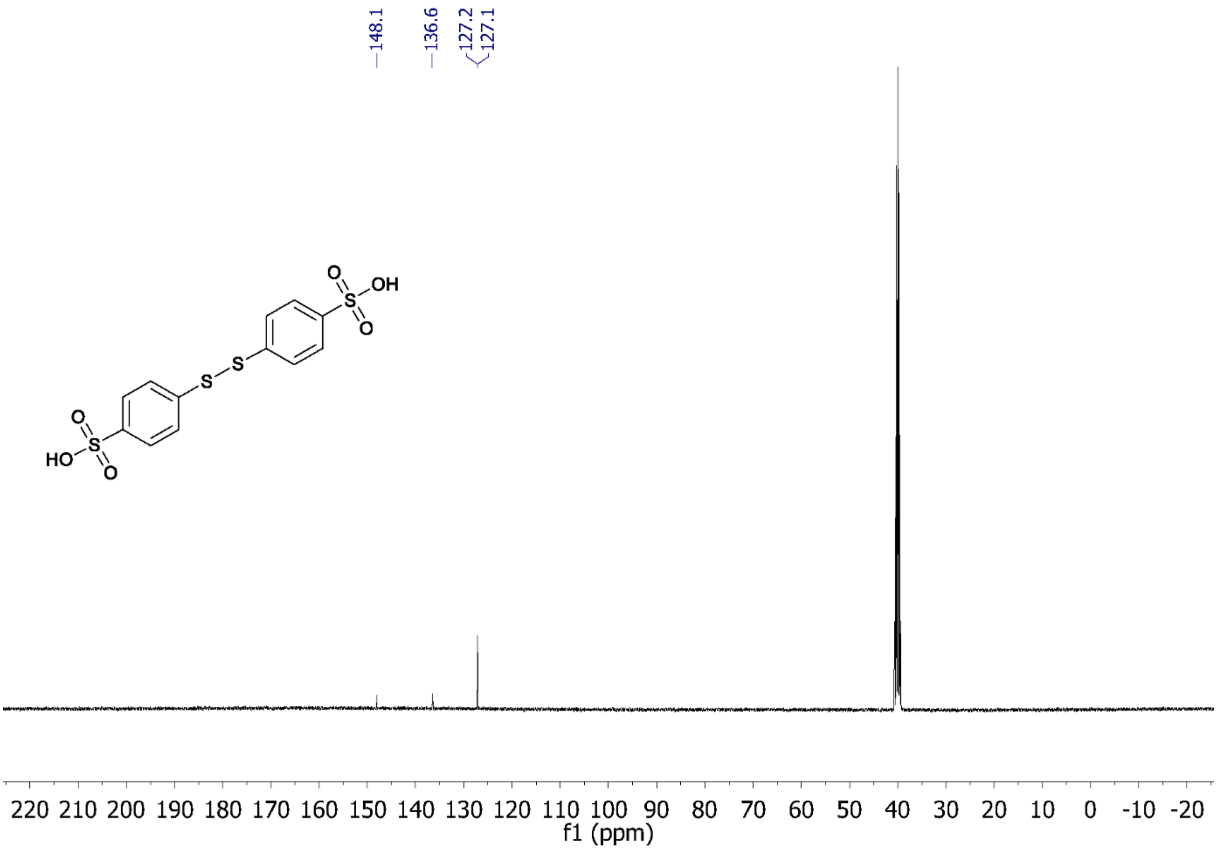

Compound **3**, ^{13}C NMR (400 MHz, DMSO-d_6).

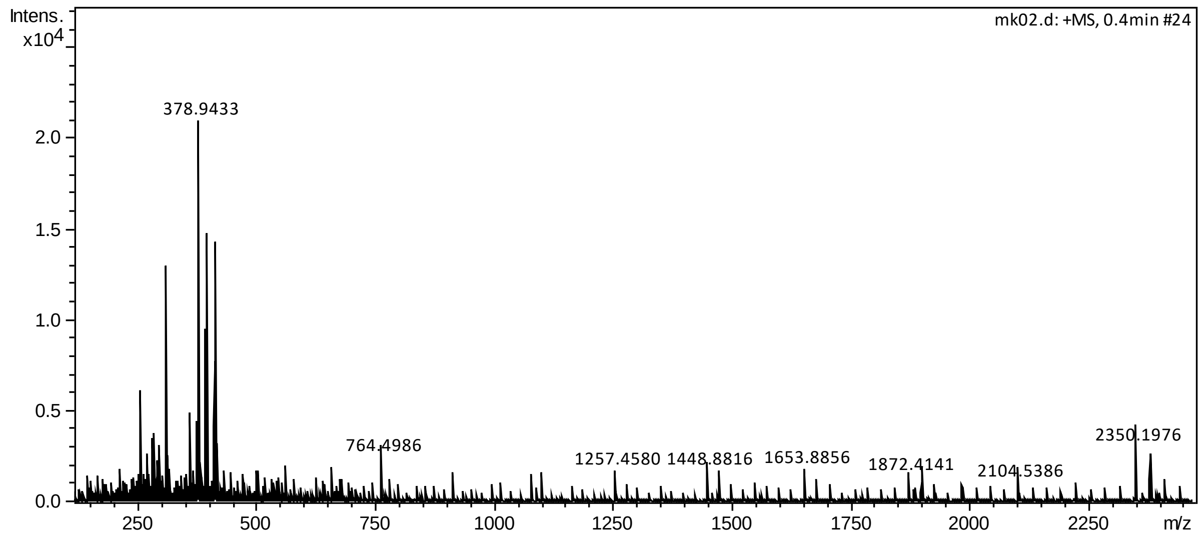

Compound **3,** HRMS APCI$^+$: $[C_{12}H_{10}O_6S_4+H]^+$ calc 378.9433.

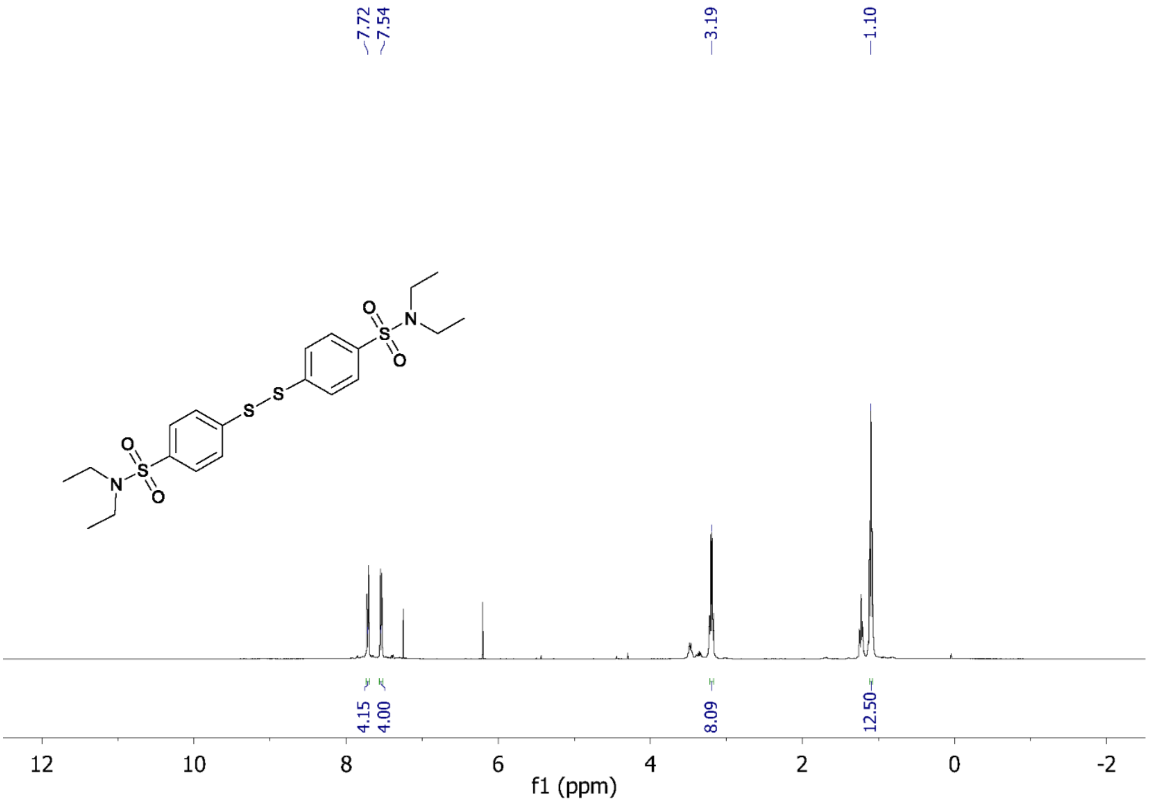

Compound **4**, ^{1}H NMR (400 MHz, $CDCl_3$).

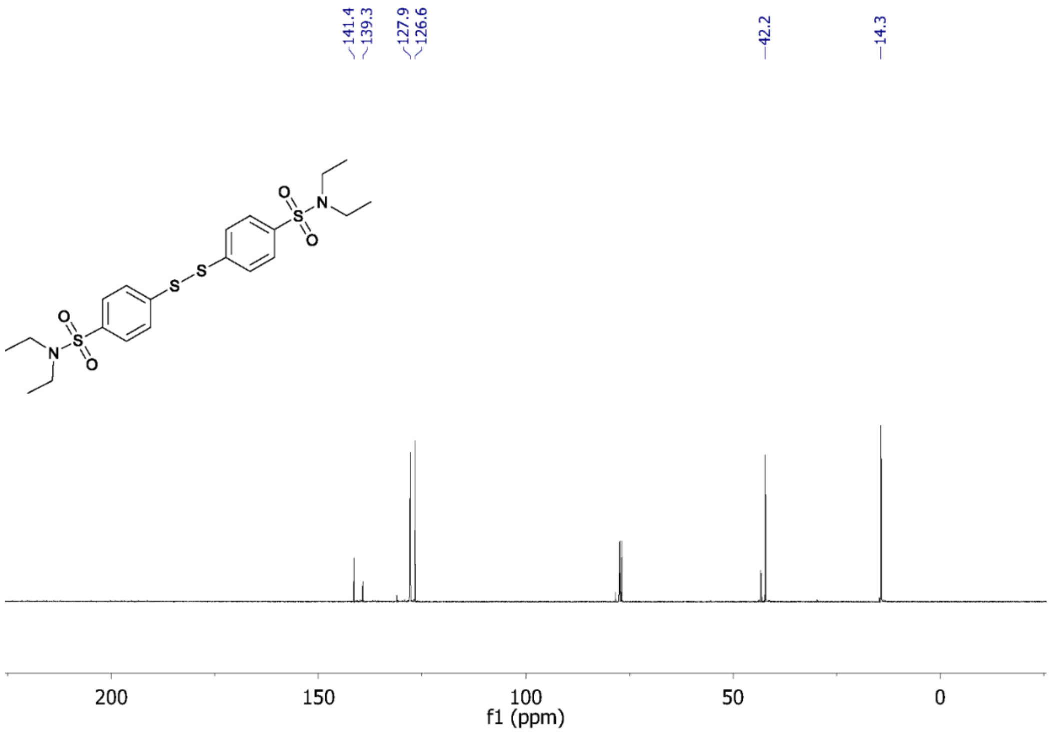

Compound **4**, ^{13}C NMR (400 MHz, $CDCl_3$).

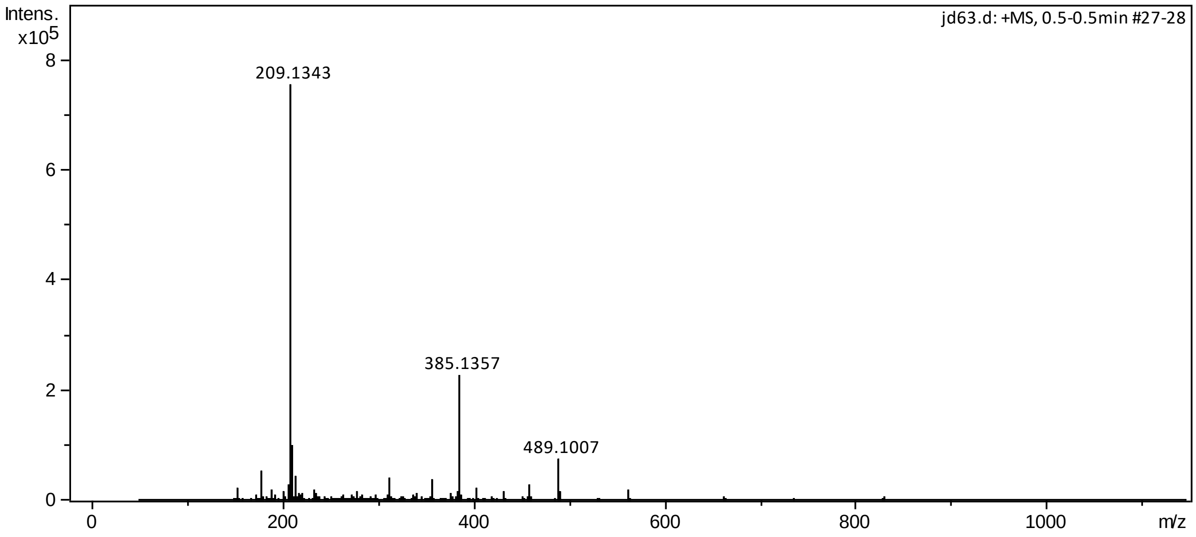

Compound **4**, HRMS ESI$^+$: $[C_{20}H_{28}N_2O_4S_4 + H]^+$ calc 489.1004.

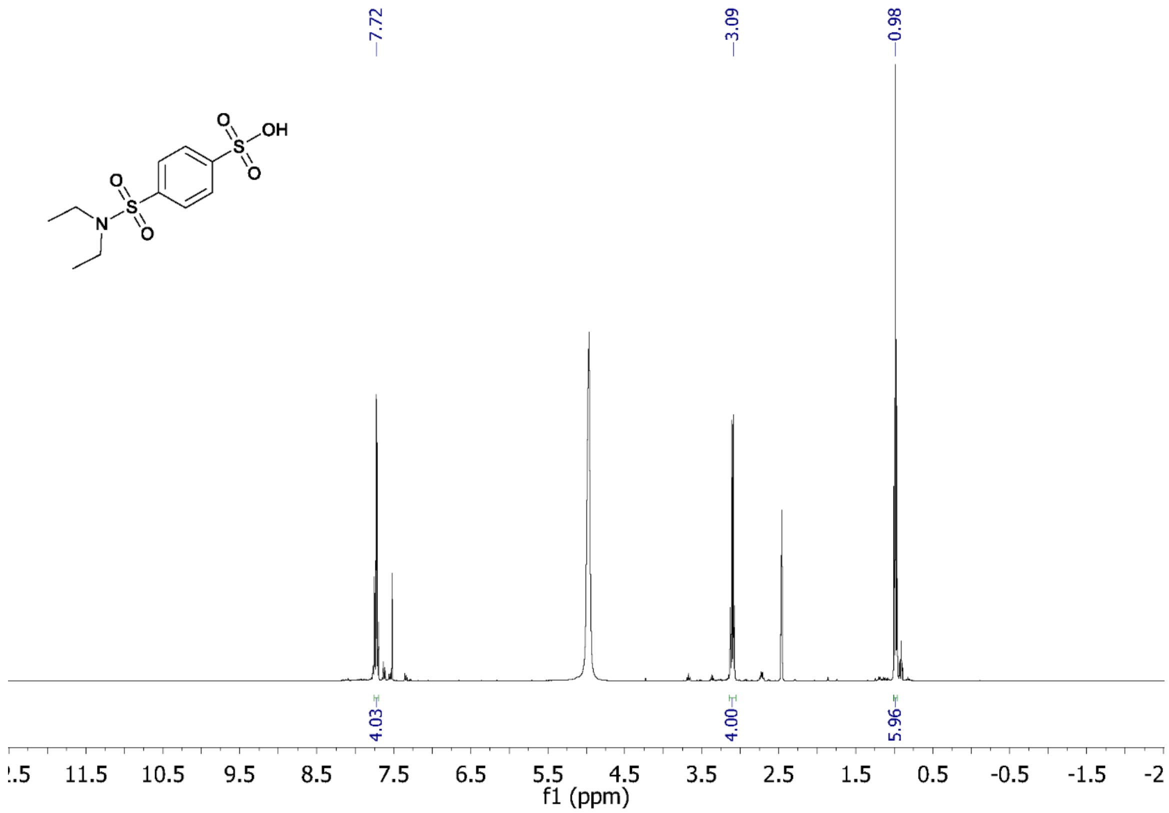

Compound **5**, ^{1}H NMR (400 MHz, DMSO-d_6).

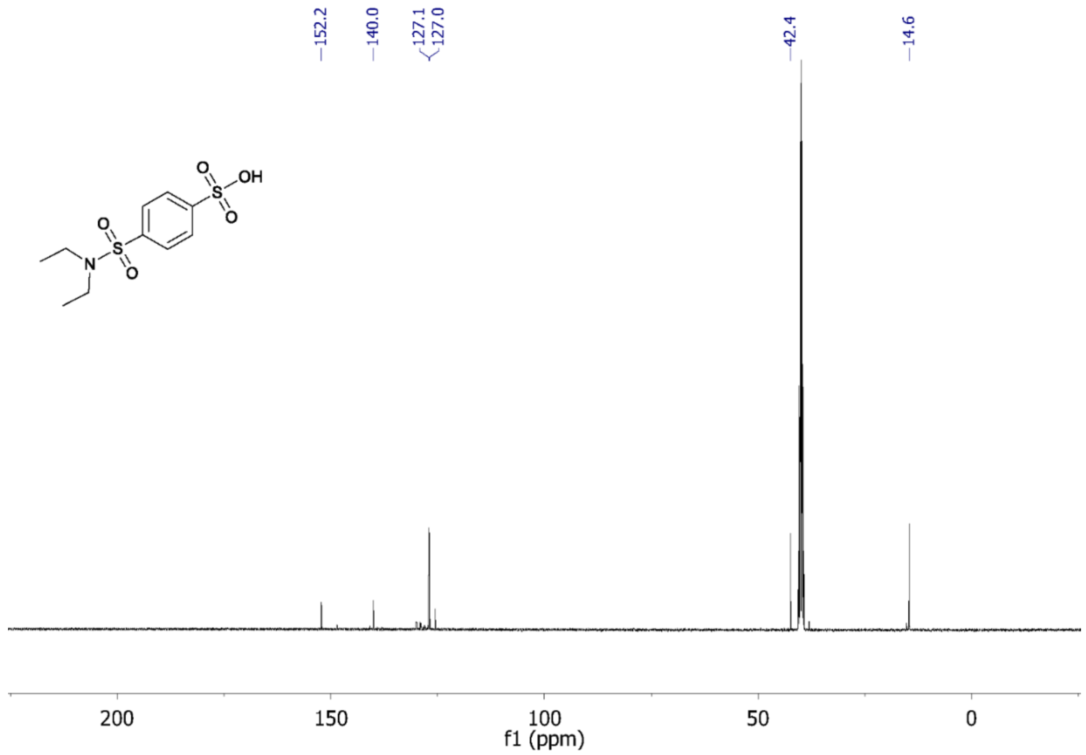

Compound **5**, ^{1}H NMR (400 MHz, DMSO-d_6).

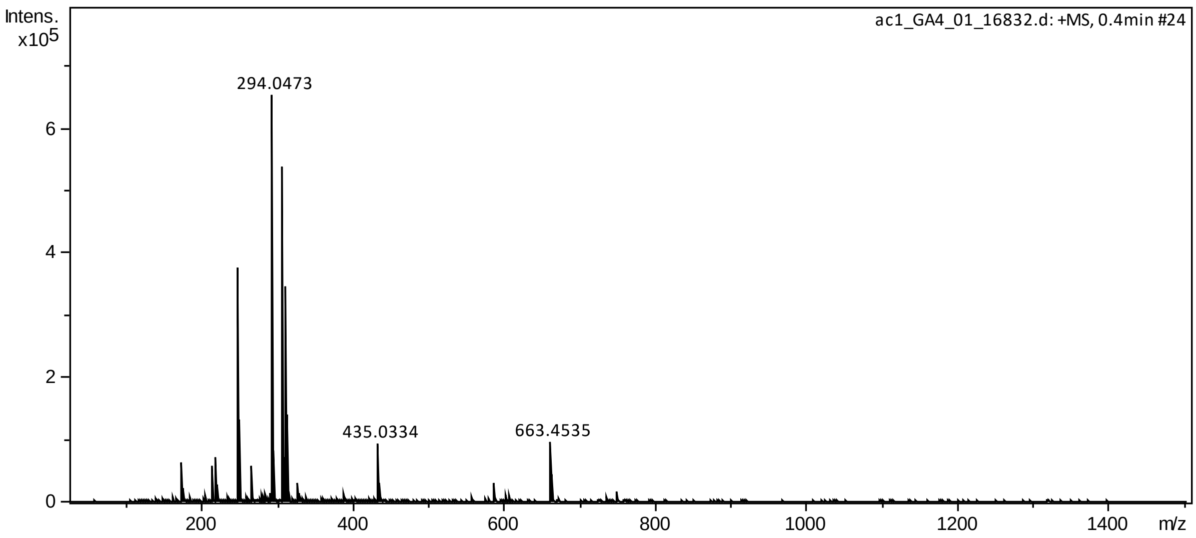

Compound **5**, HRMS ESI$^+$:[$C_{10}H_{15}NO_5S_2$ +H]$^+$ calc. 294.0465.

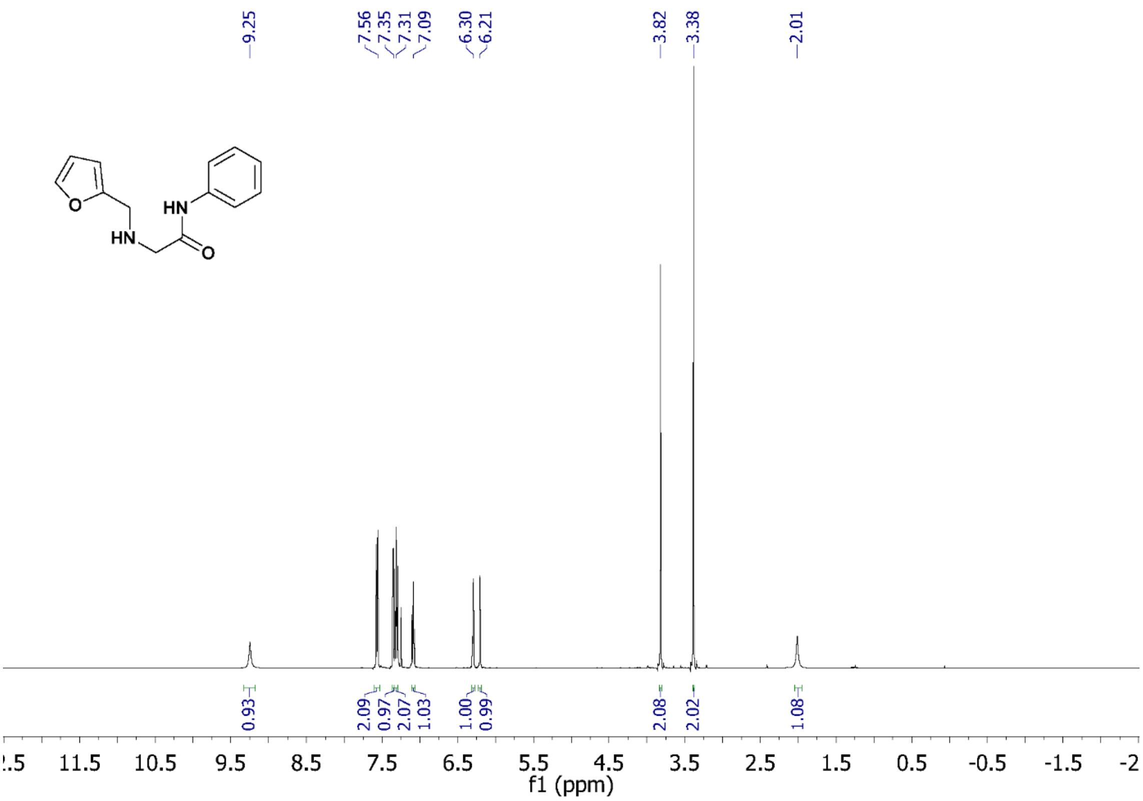

Compound **8a**, ^{1}H NMR (400 MHz, $CDCl_3$).

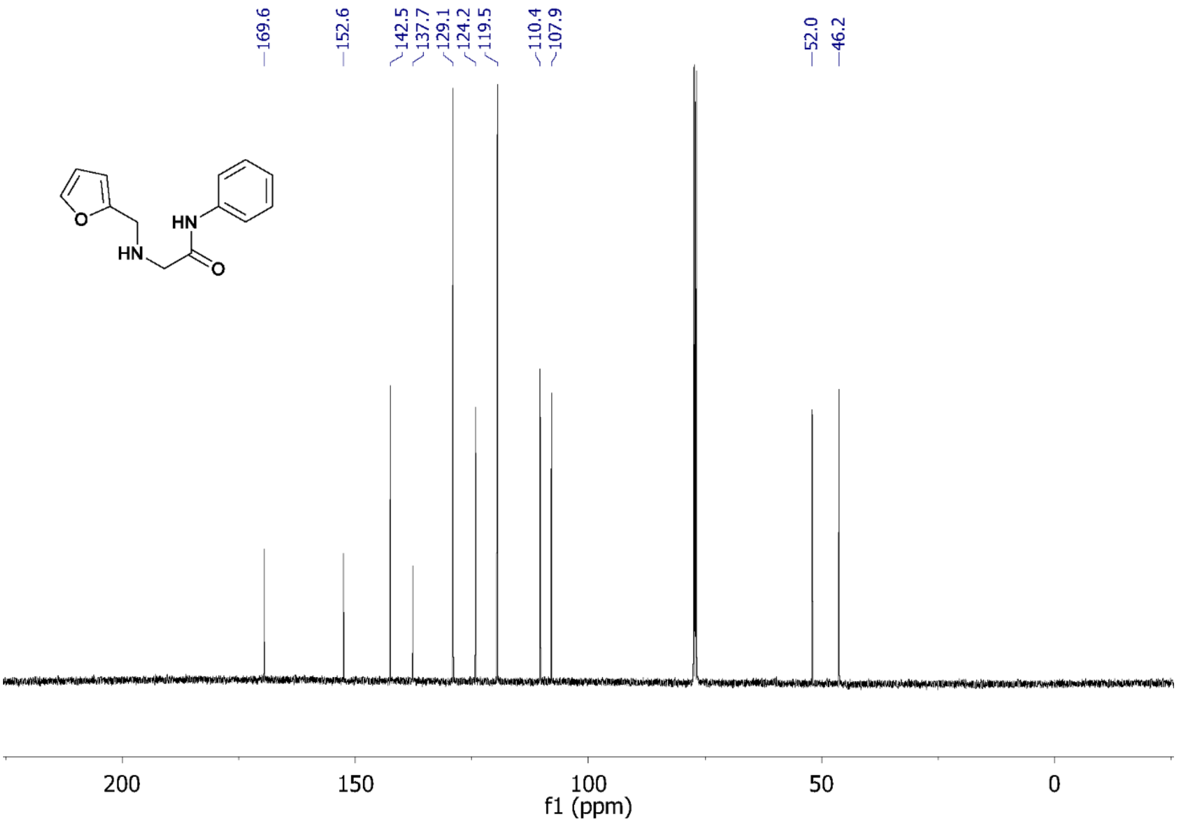

Compound **8a**, ^{13}C NMR (400 MHz, $CDCl_3$).

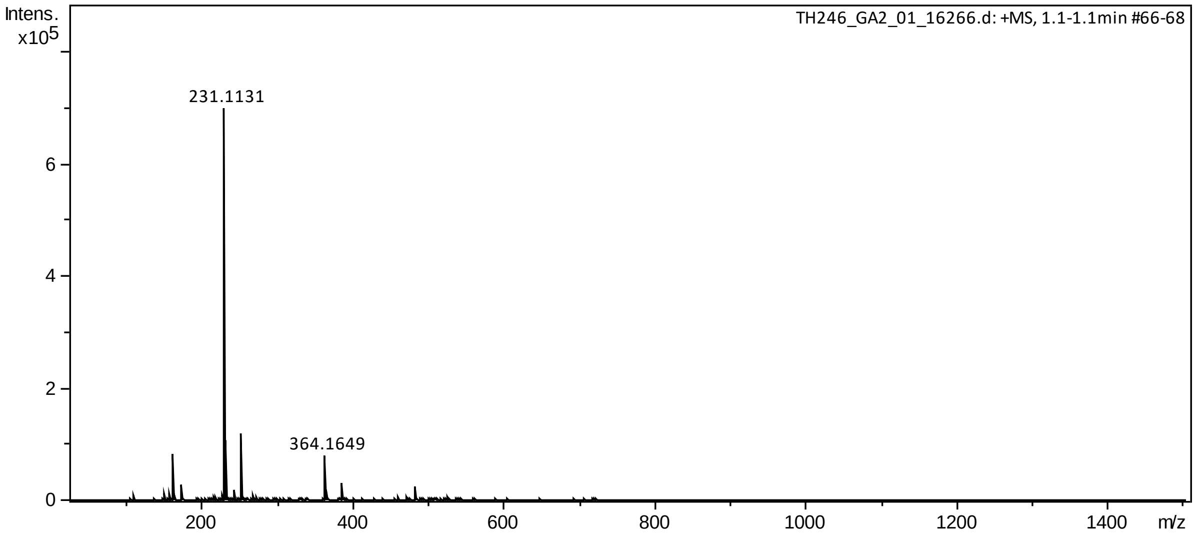

Compound **8a**, HRMS ESI$^+$: $[C_{13}H_{14}N_2O_2+H]^+$ calc 231.1128.

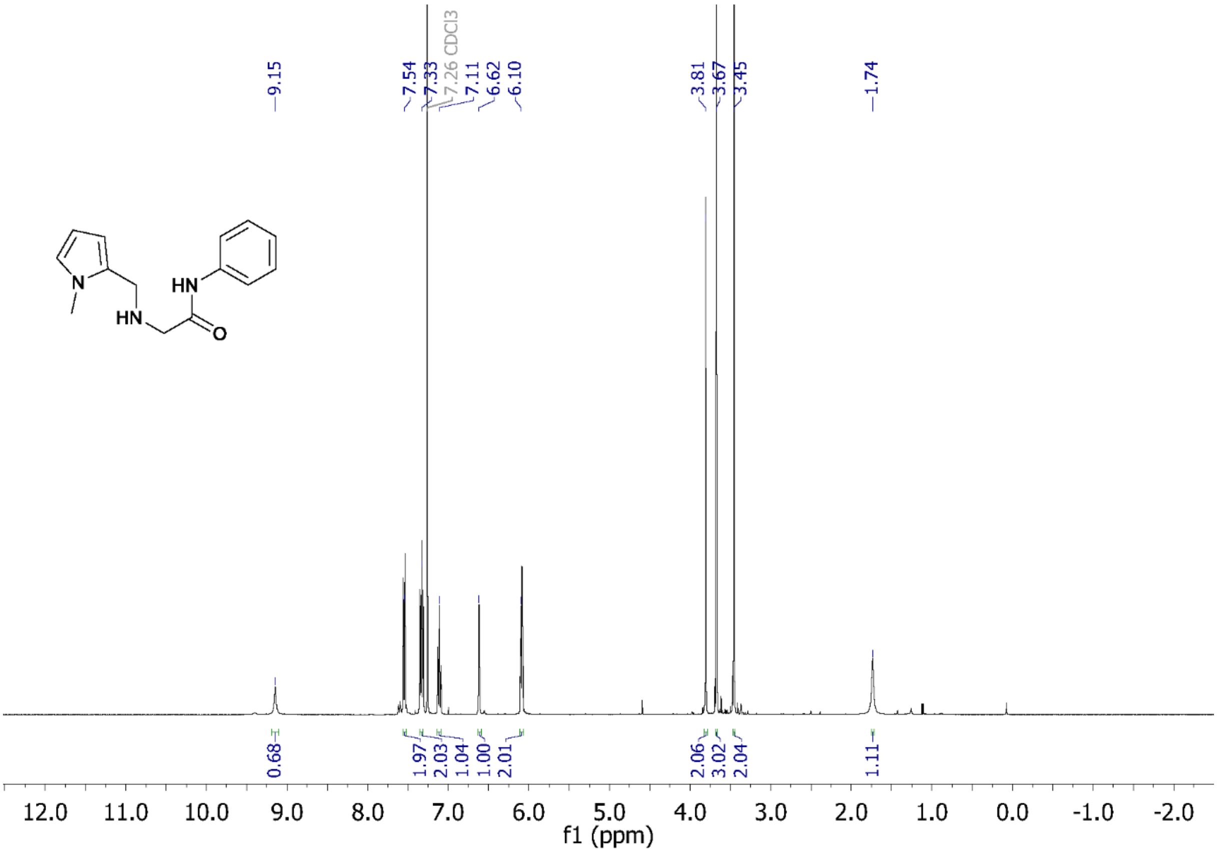

Compound **8b**, ^{1}H NMR (400 MHz, $CDCl_3$).

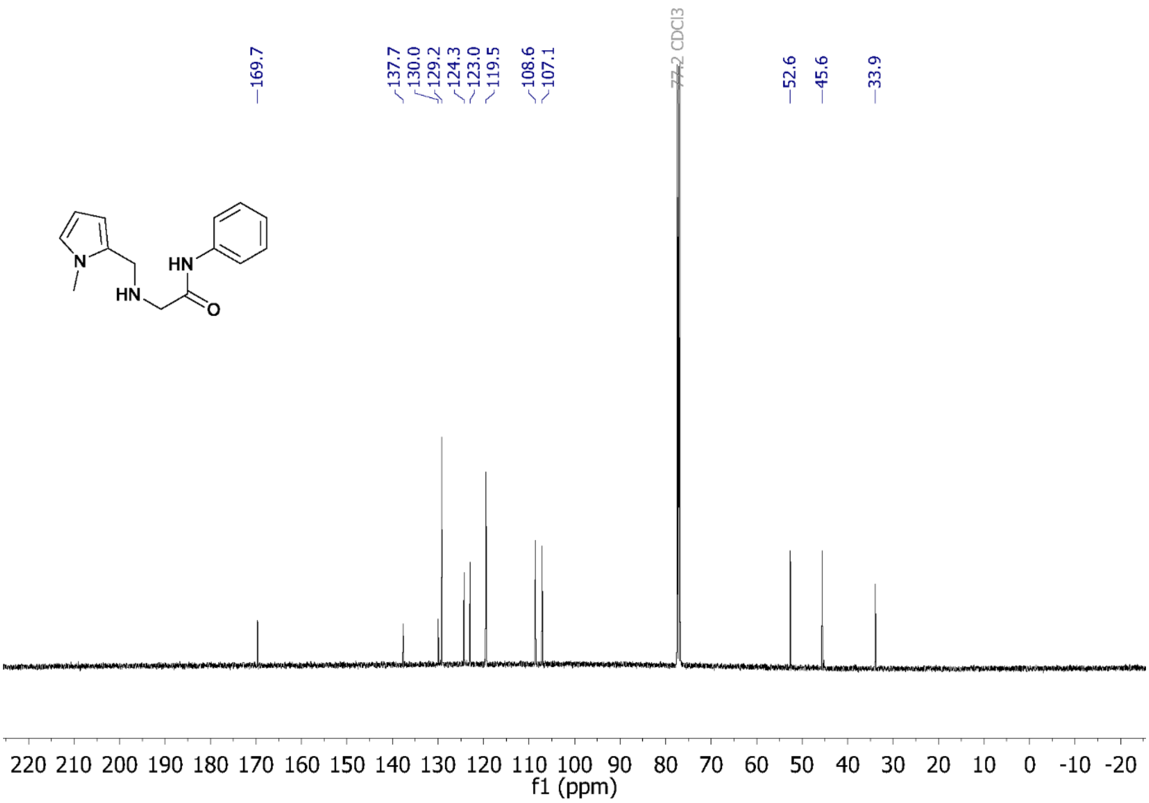

Compound **8b**, ^{13}C NMR (400 MHz, $CDCl_3$).

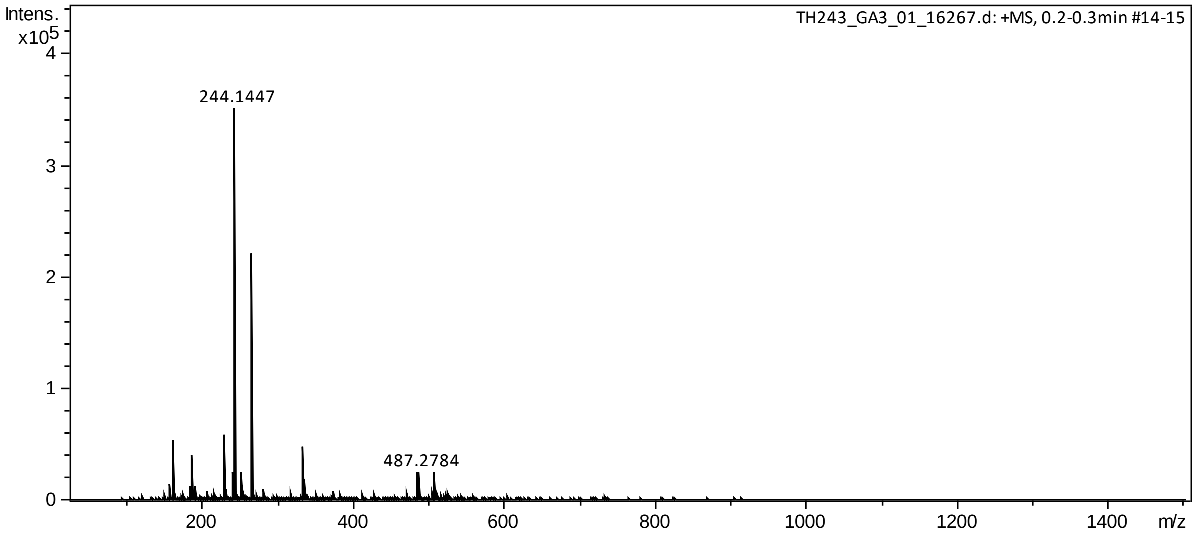

Compound **8b**, HRMS ESI$^+$: $[C_{14}H_{17}N_3O+H]^+$ calc 244.1444.

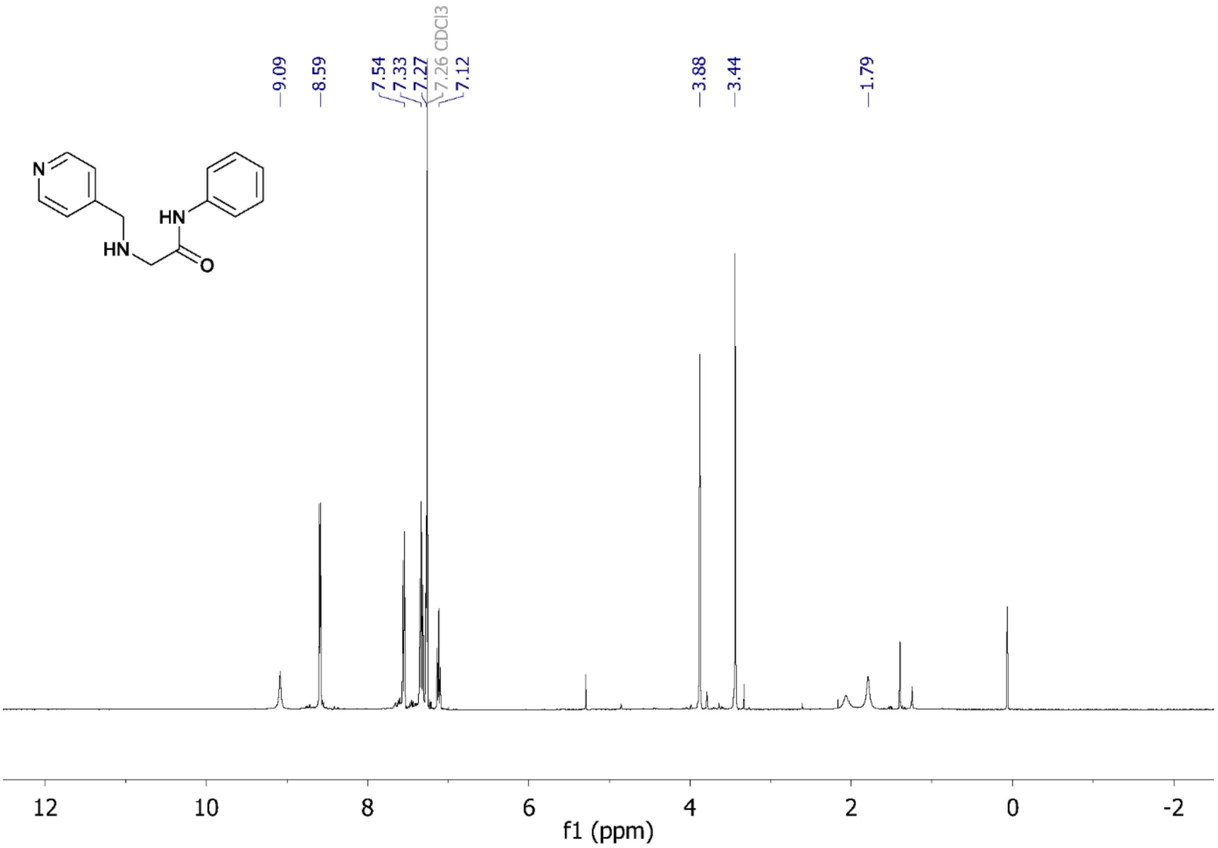

Compound **8c**, ^{1}H NMR (400 MHz, $CDCl_3$).

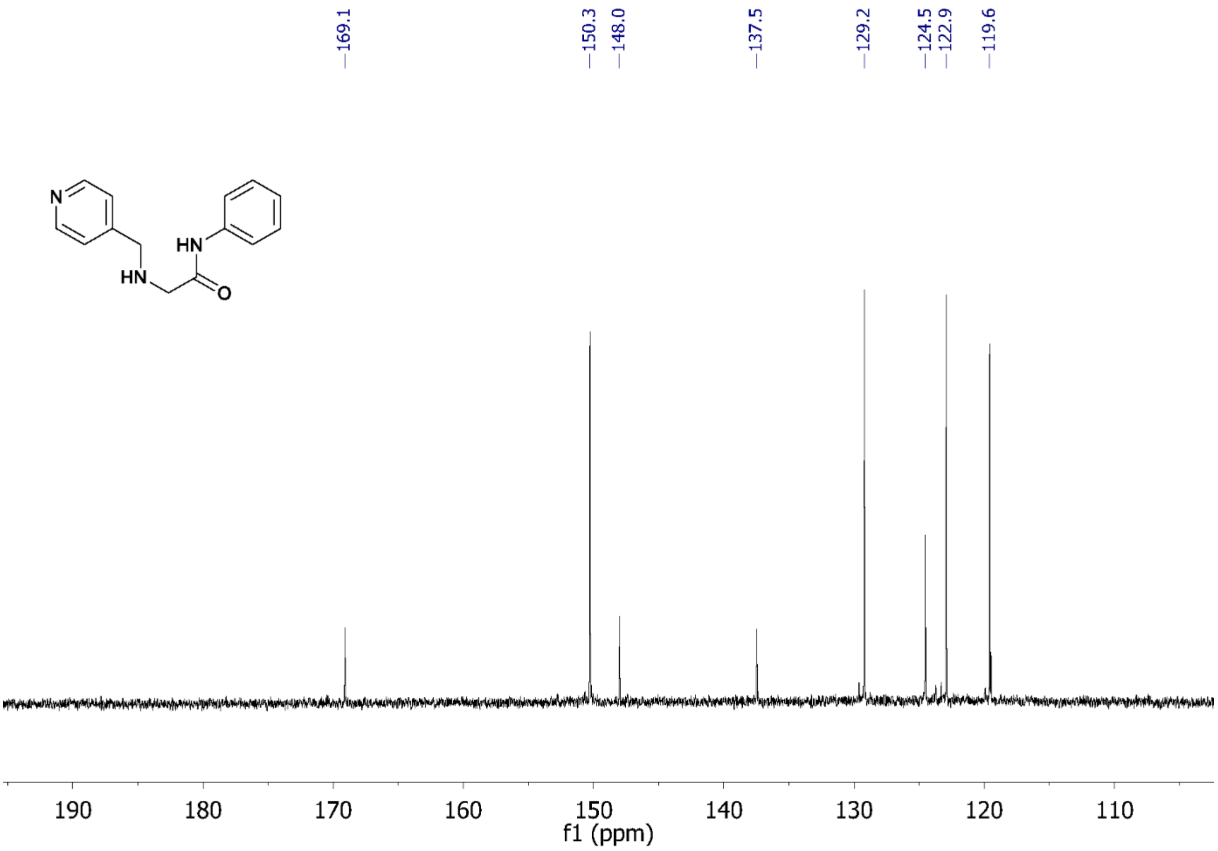

Compound **8c**, ^{13}C NMR (400 MHz, $CDCl_3$).

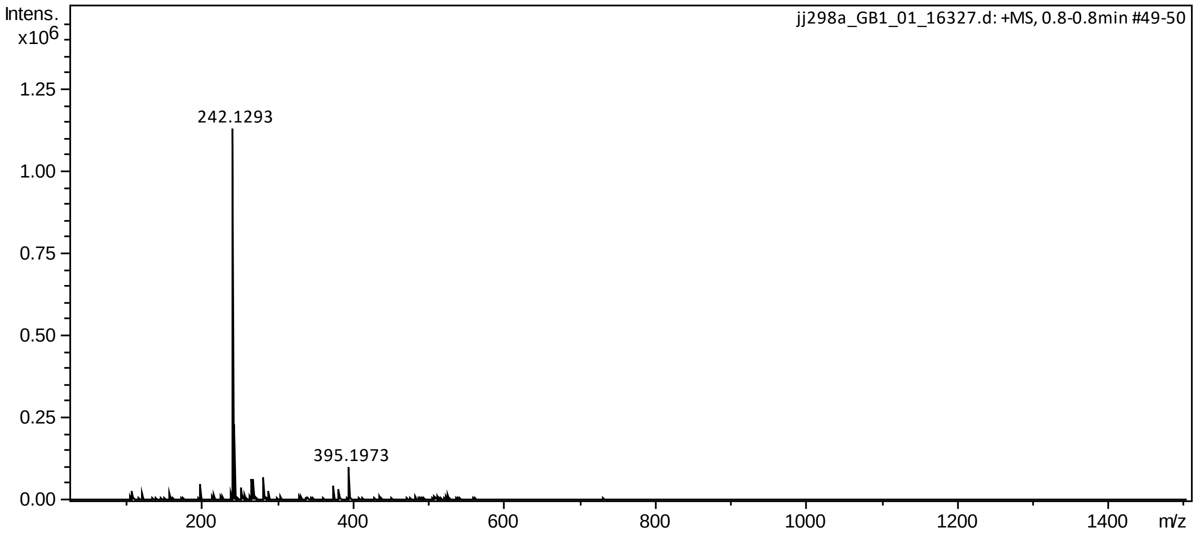

Compound **8c**, HRMS (ESI)$^+$: $[C_{14}H_{15}N_3O+H]^+$ calc 242.1288

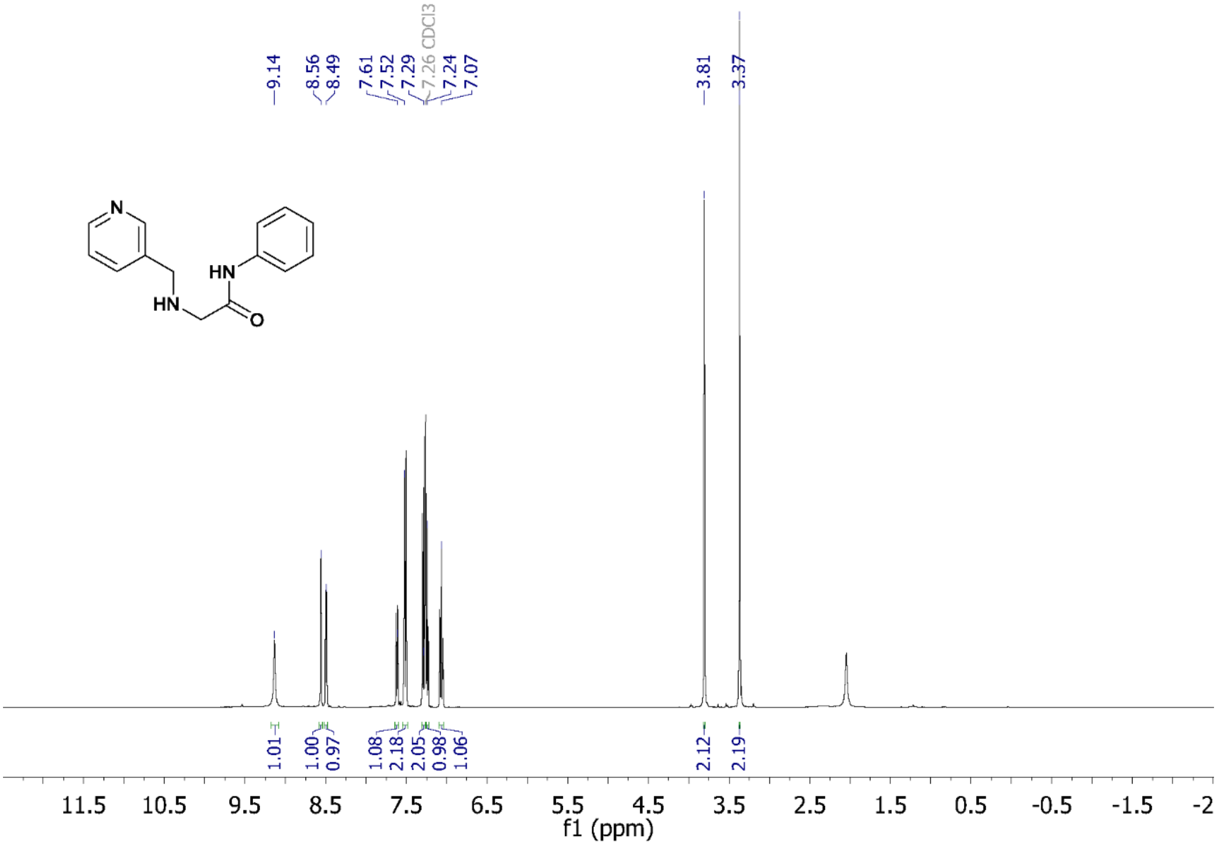

Compound **8d**, ^{1}H NMR (400 MHz, $CDCl_3$).

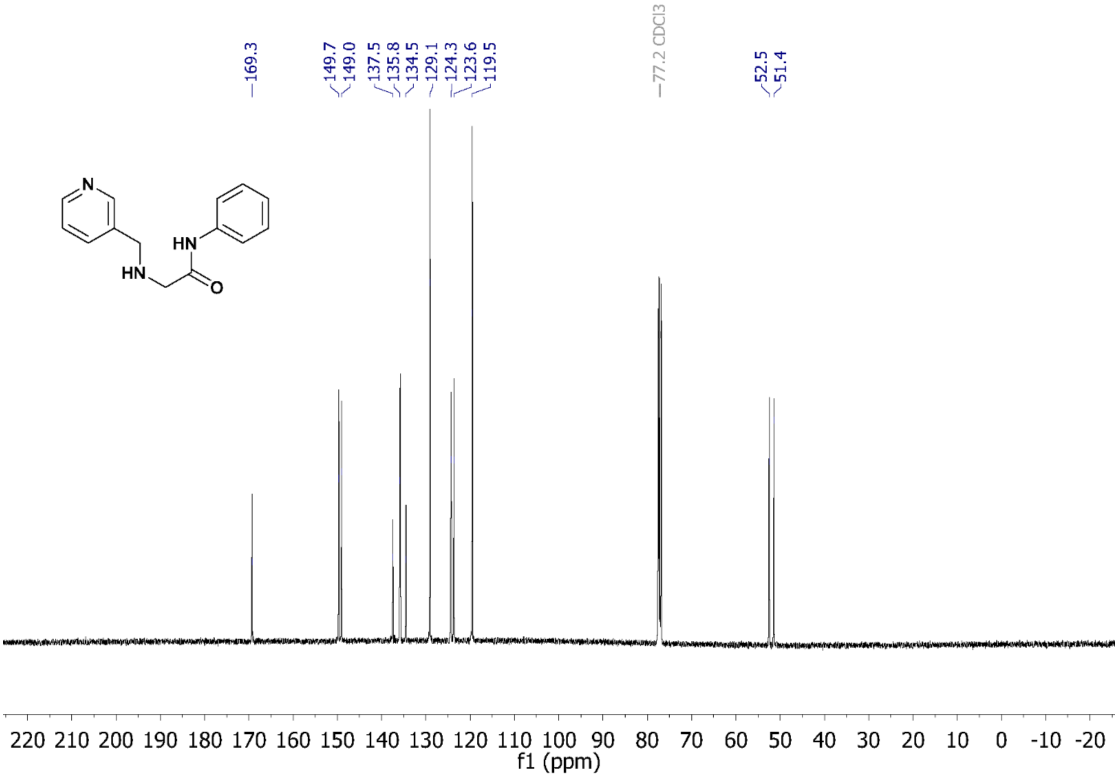

Compound **8d**, ^{13}C NMR (400 MHz, $CDCl_3$).

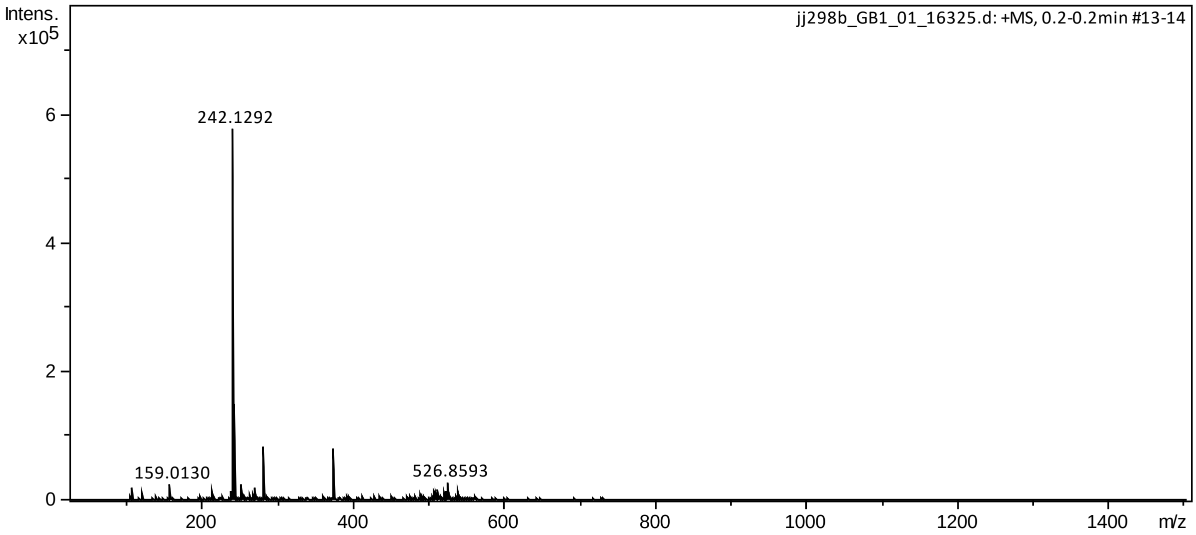

Compound **8d**, HRMS ESI$^+$: $[C_{14}H_{15}N_3O+H]^+$ calc 242.1288.

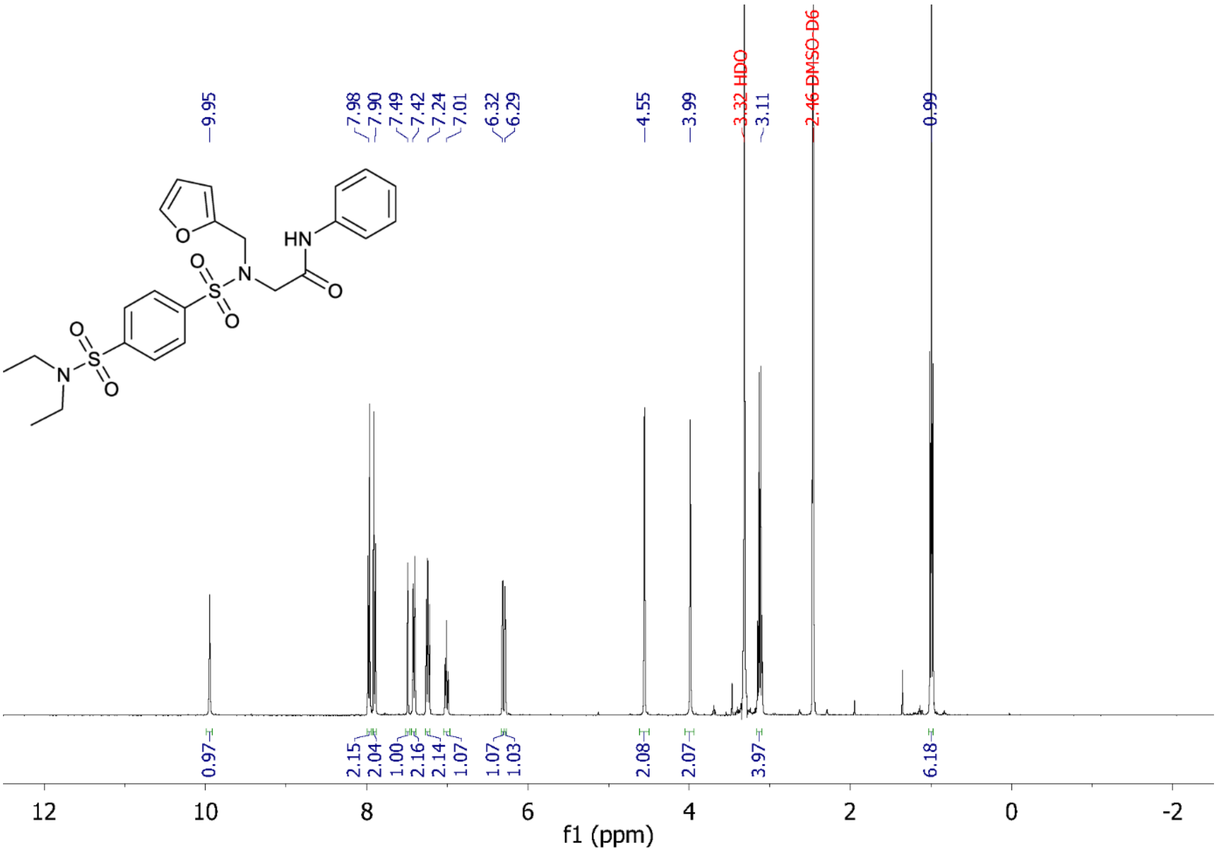

Compound **1a**, ^{1}H NMR (400 MHz, DMSO-d_6).

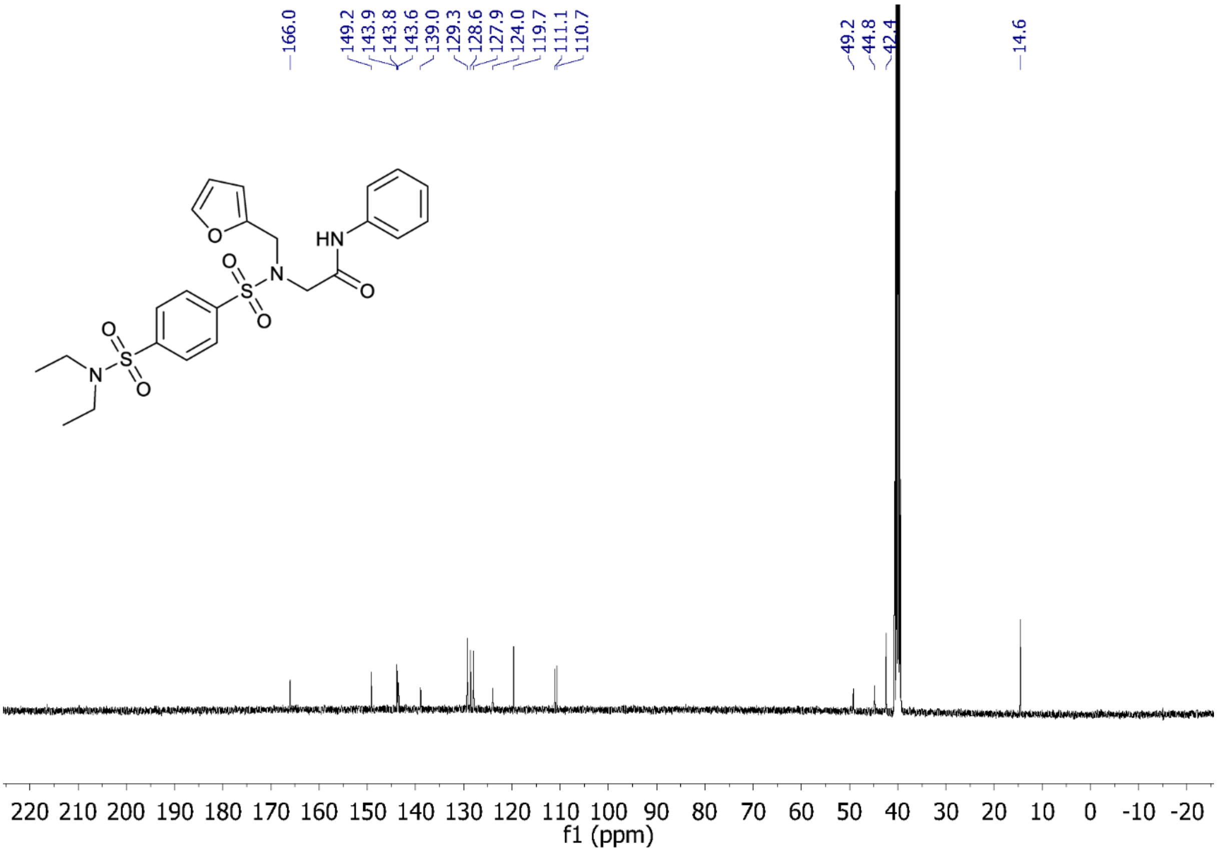

Compound **1a**, ^{13}C NMR (400 MHz, DMSO-d_6).

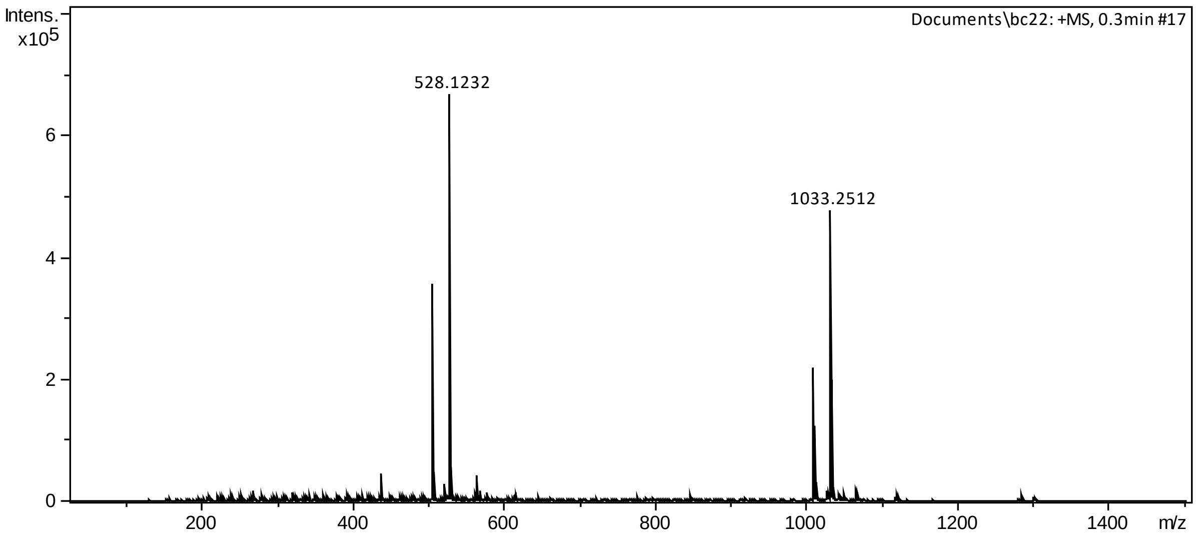

Compound **1a**, HRMS ESI$^+$: $[C_{23}H_{27}N_3O_6S_2+Na]^+$ calc 528.1233.

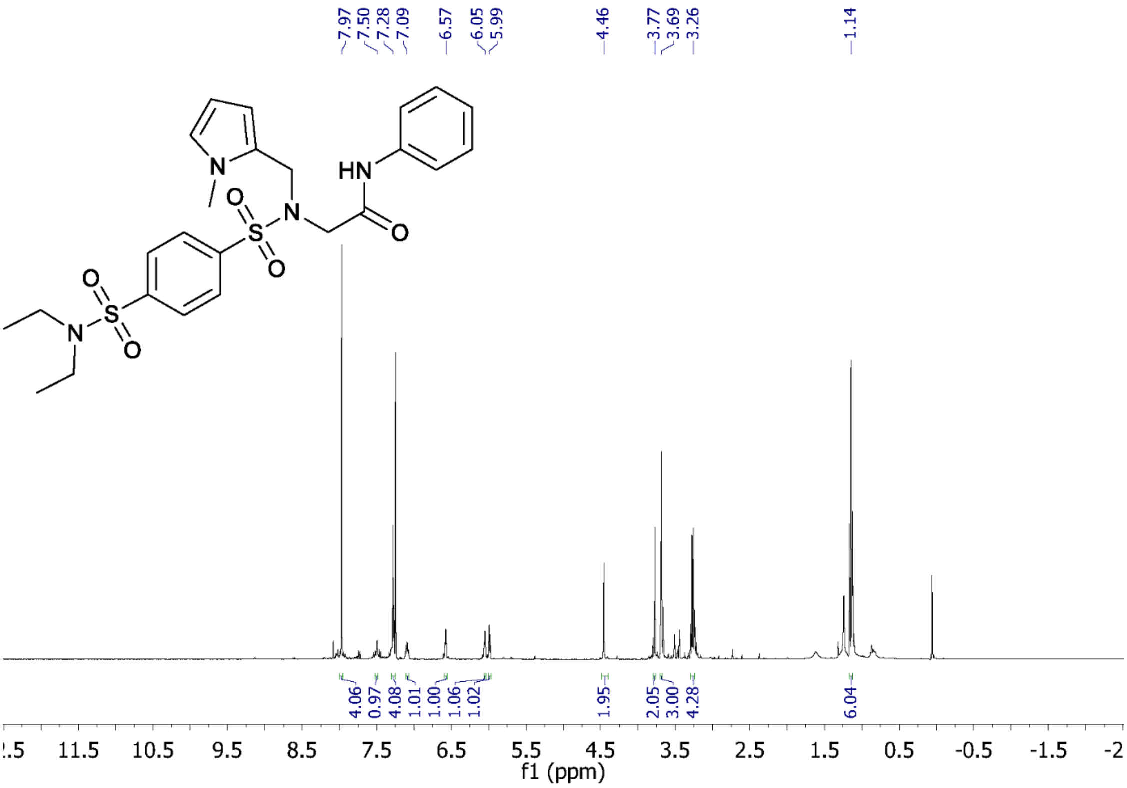

Compound **1b**, ^{1}H NMR (400 MHz, $CDCl_3$).

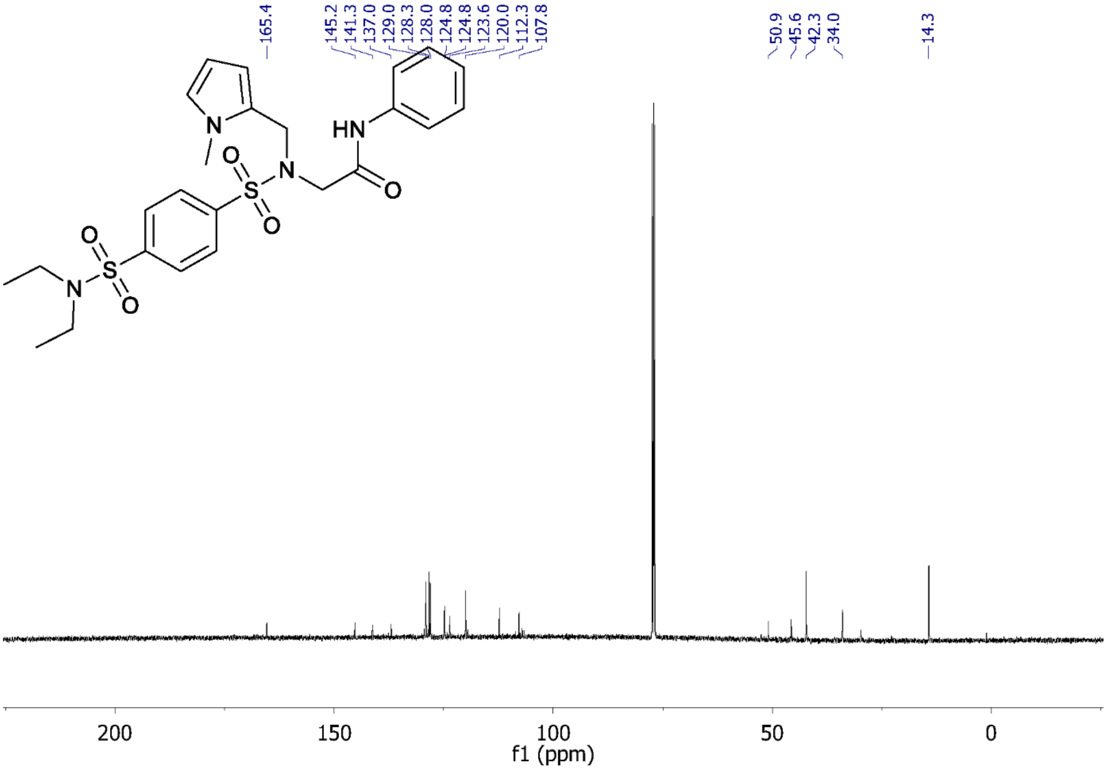

Compound **1b**, ^{13}C NMR (400 MHz, $CDCl_3$).

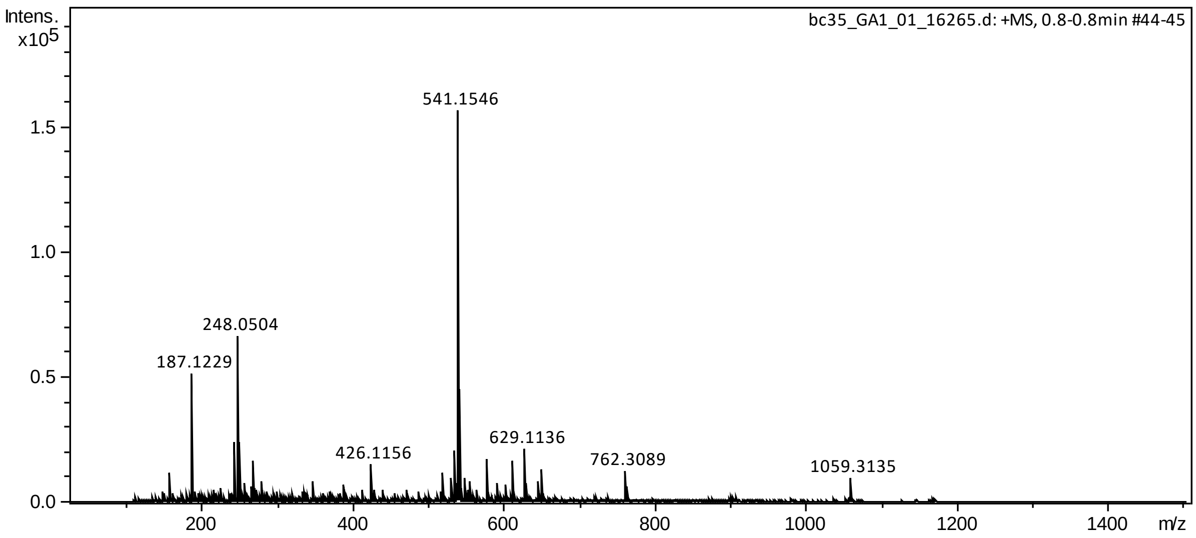

Compound **1b**, HRMS ESI+: $[C_{24}H_{30}N_4O_5S_2+Na]^+$ calc 541.1550.

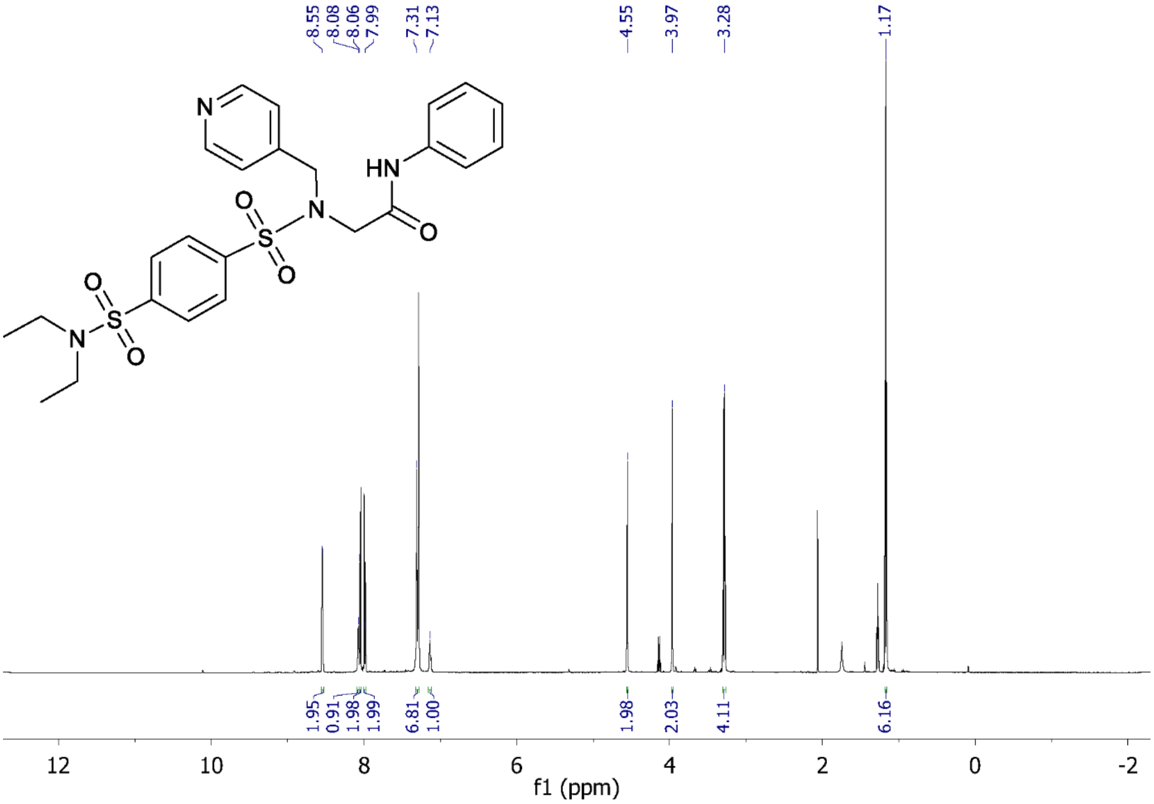

Compound **1c**, ^{1}H NMR (400 MHz, $CDCl_3$).

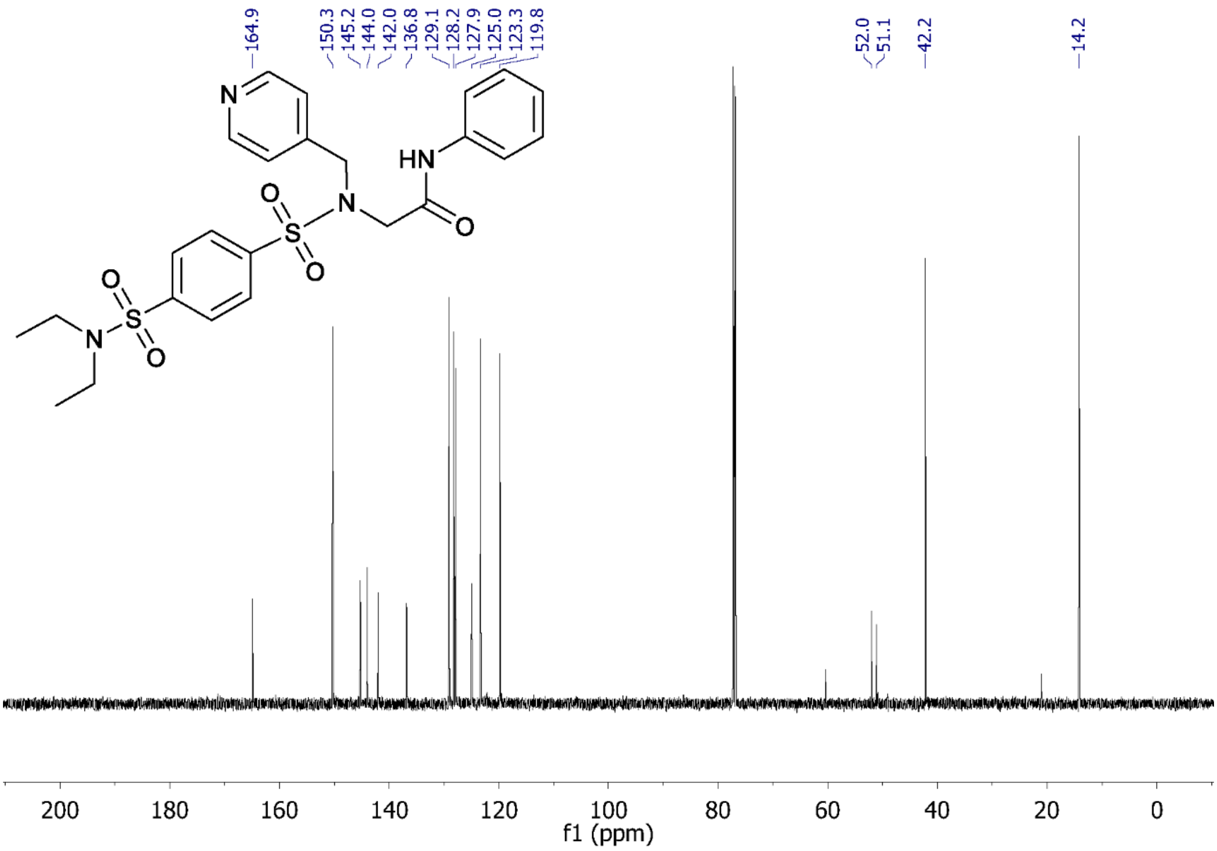

Compound **1c**, ^{13}C NMR (400 MHz, $CDCl_3$).

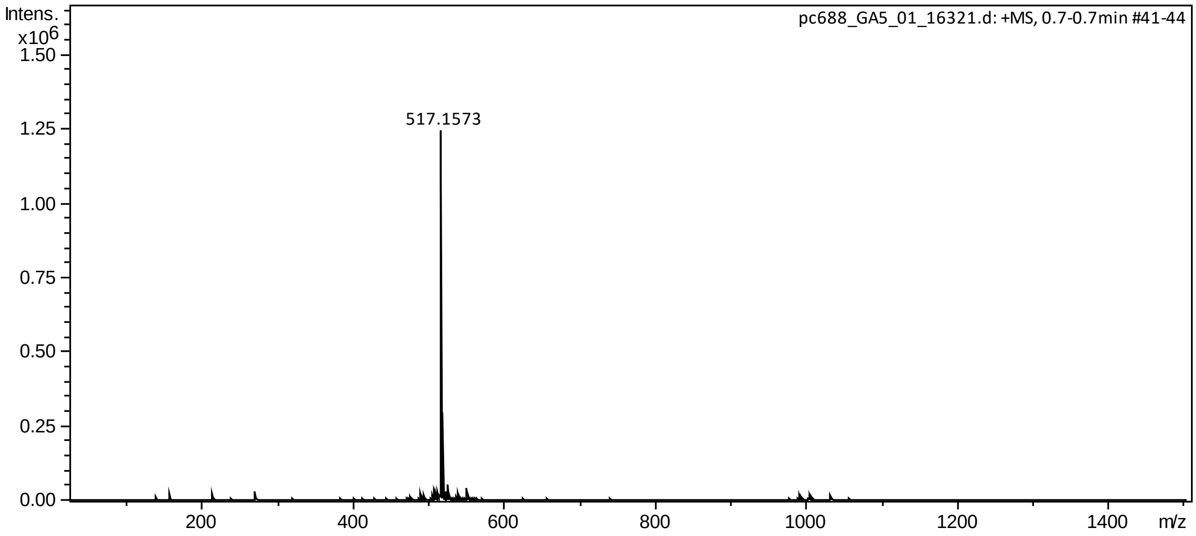

Compound **1c**, HRMS ESI$^+$[$C_{24}H_{28}N_4O_5S_2$+H]$^+$ calc 517.1574.

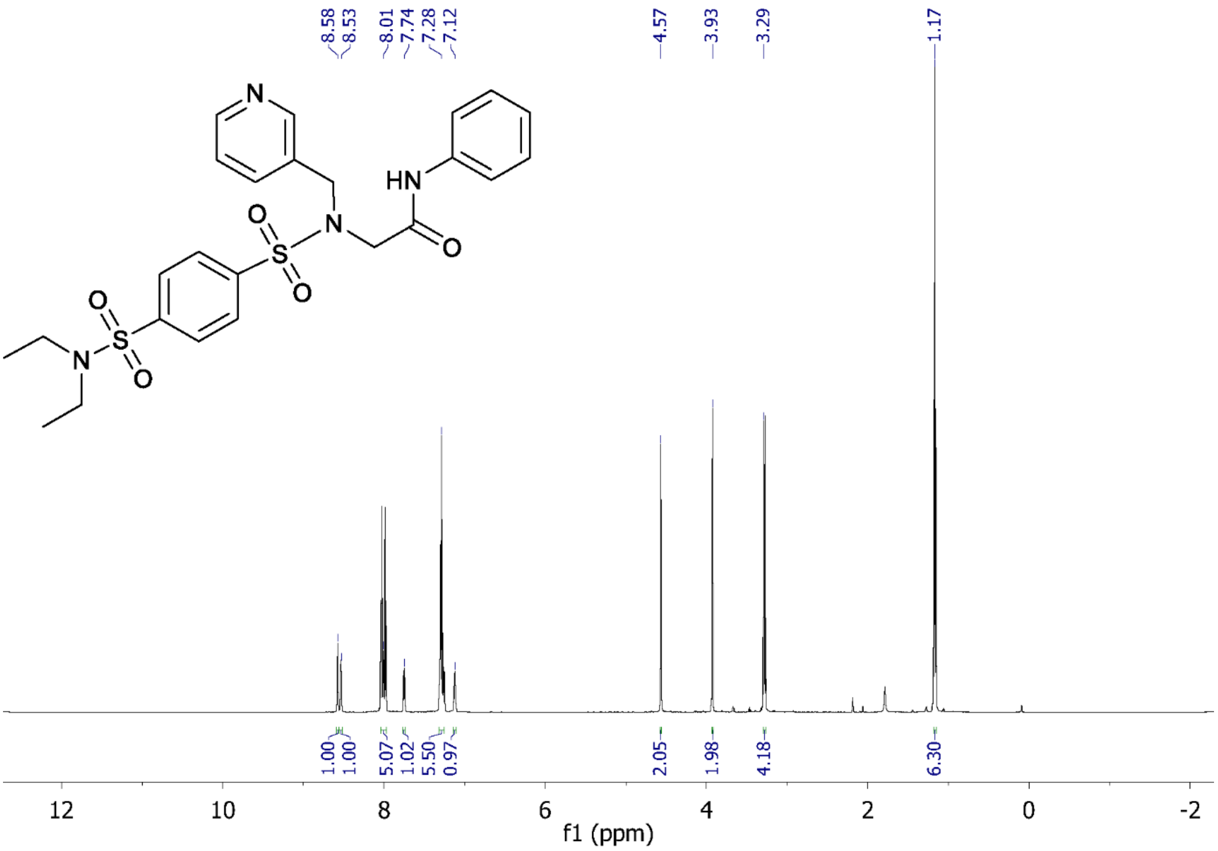

Compound **1d**, ^{1}H NMR (400 MHz, $CDCl_3$).

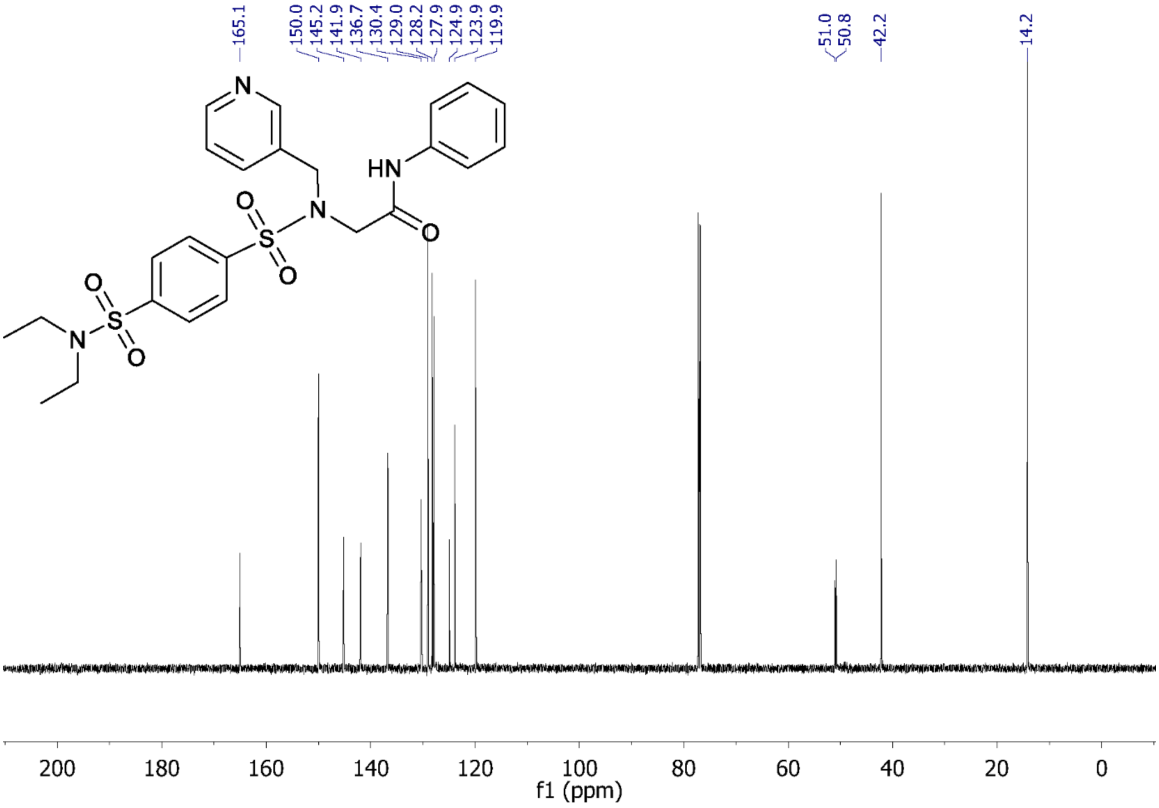

Compound **1d**, ^{13}C NMR (400 MHz, $CDCl_3$).

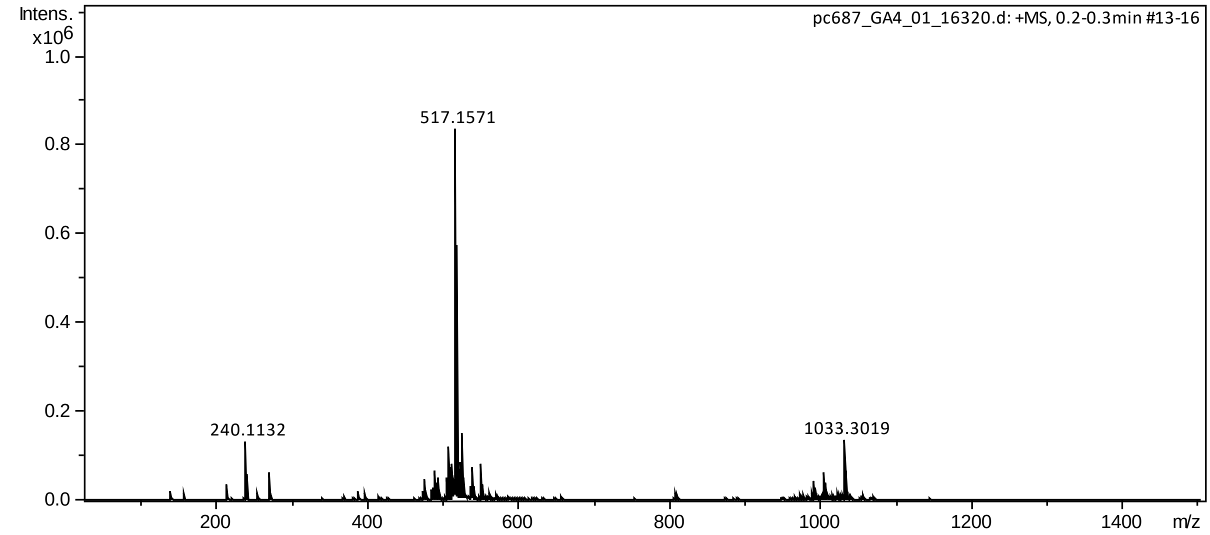

Compound **1d**, HRMS ESI$^+$[$C_{24}H_{28}N_4O_5S_2$+H]$^+$ calc 517.1574.

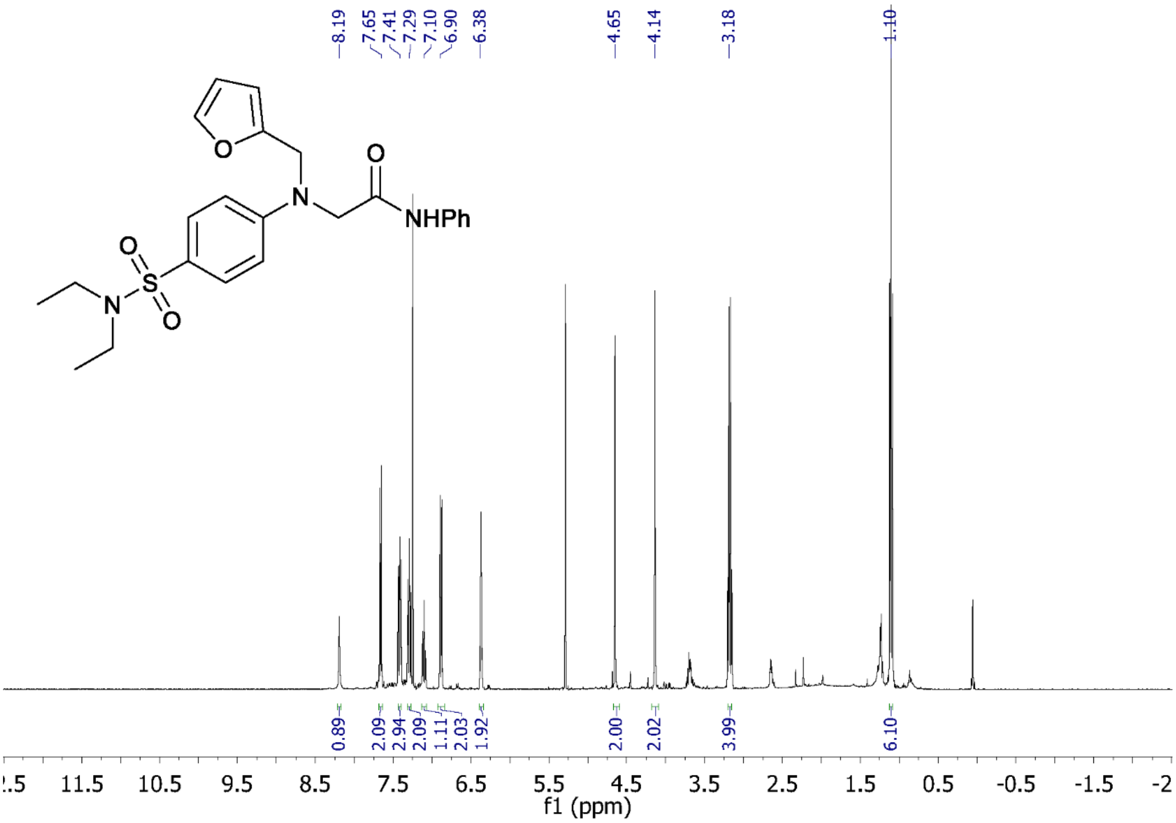

Compound **2a**, ^{1}H NMR (400 MHz, $CDCl_3$).

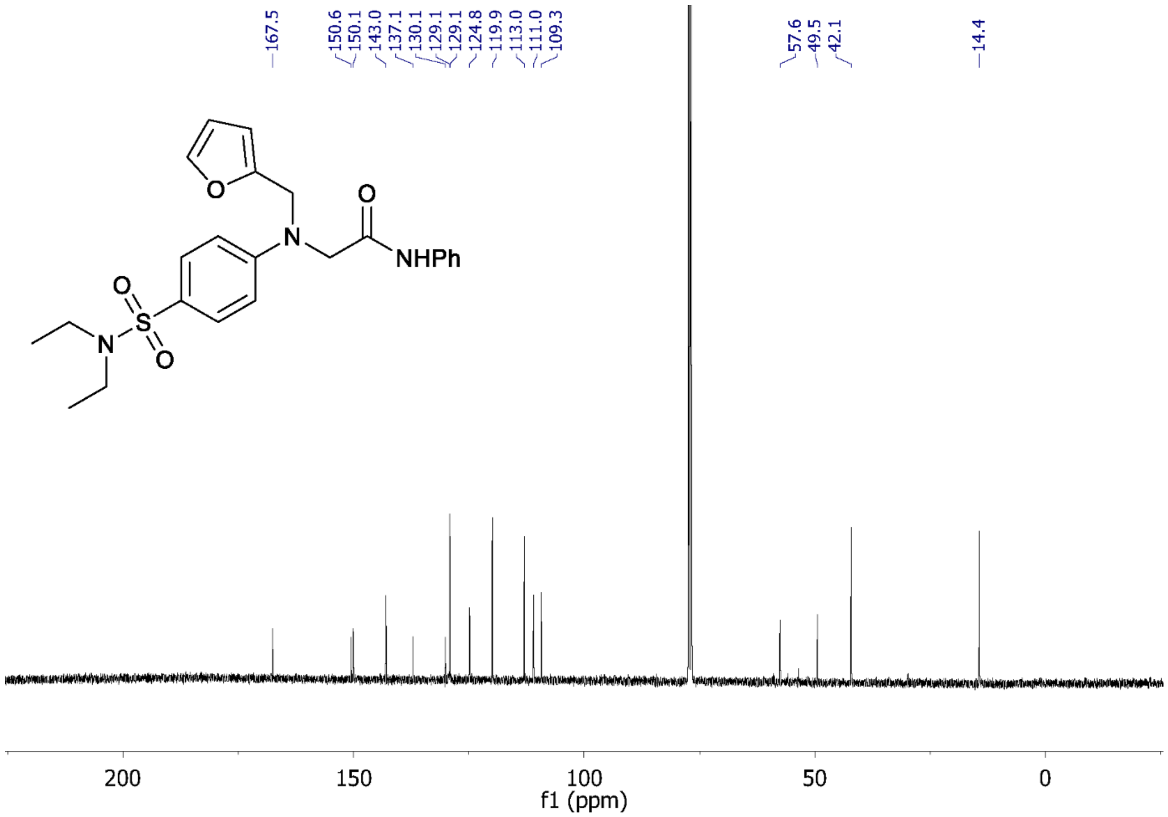

Compound **2a**, ^{13}C NMR (400 MHz, $CDCl_3$).

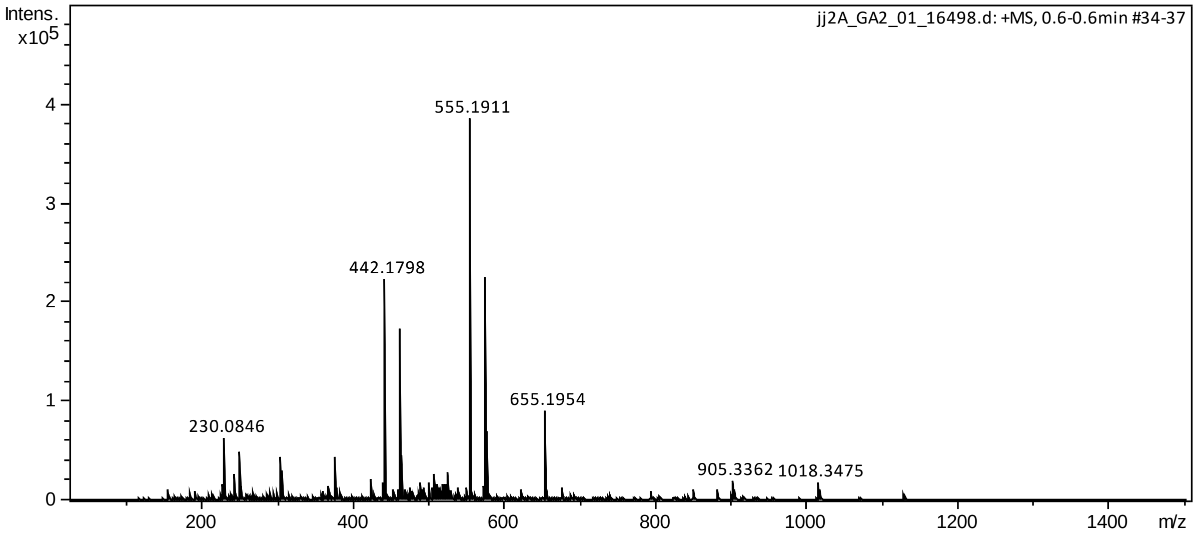

Compound **2a**, HRMS ESI$^+$: $[C_{23}H_{27}N_3O_3S+H]^+$ calc 442.1795.

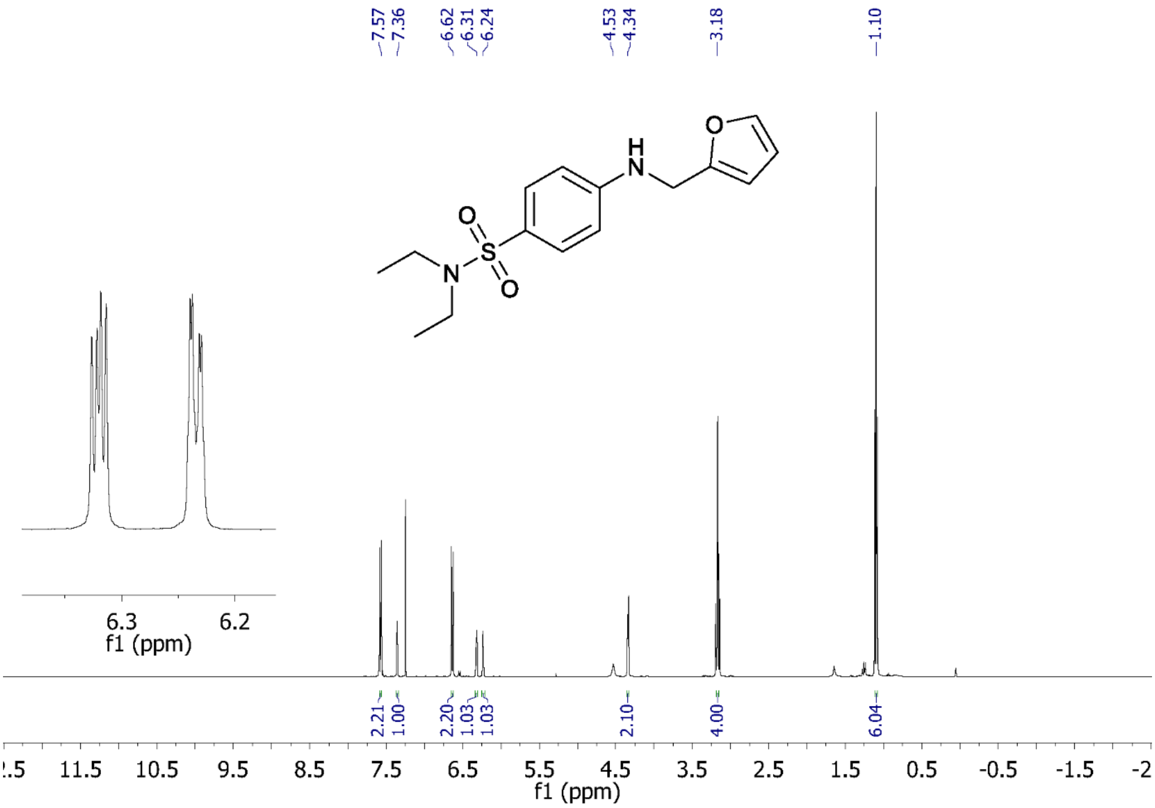

Compound **2b**, ^{1}H NMR (400 MHz, $CDCl_3$).

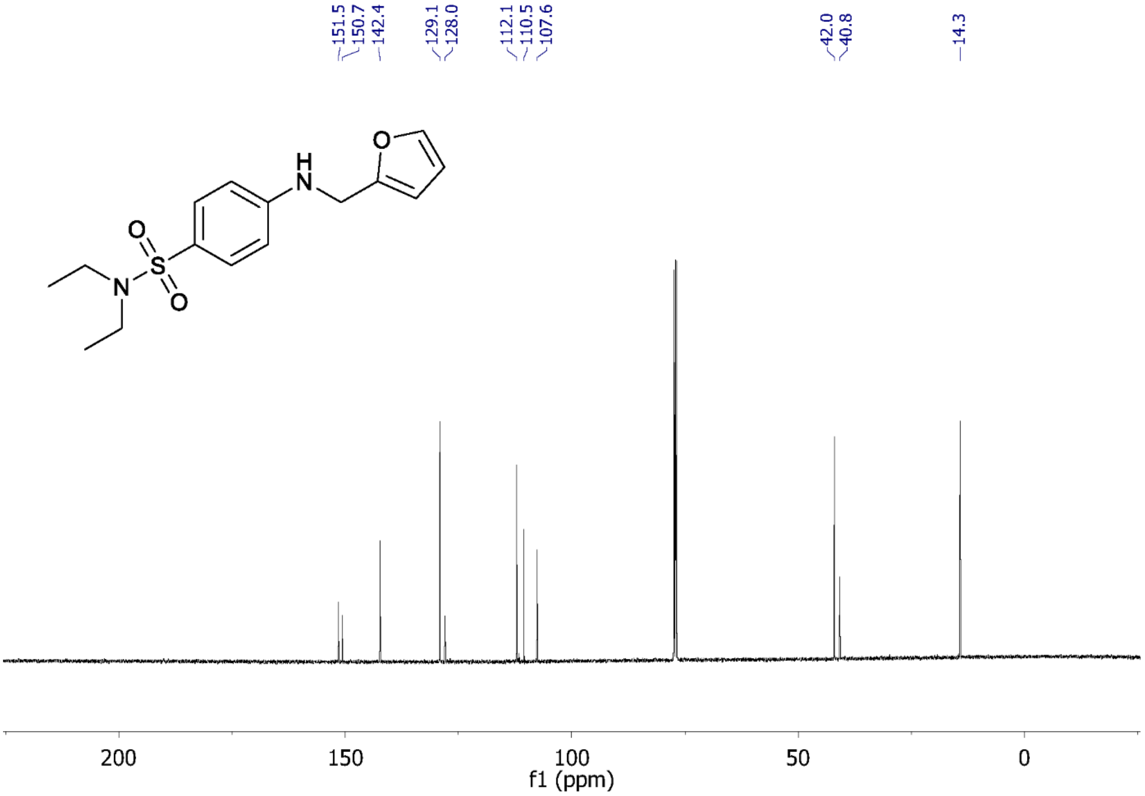

Compound **2b**, ^{13}C NMR (400 MHz, $CDCl_3$).

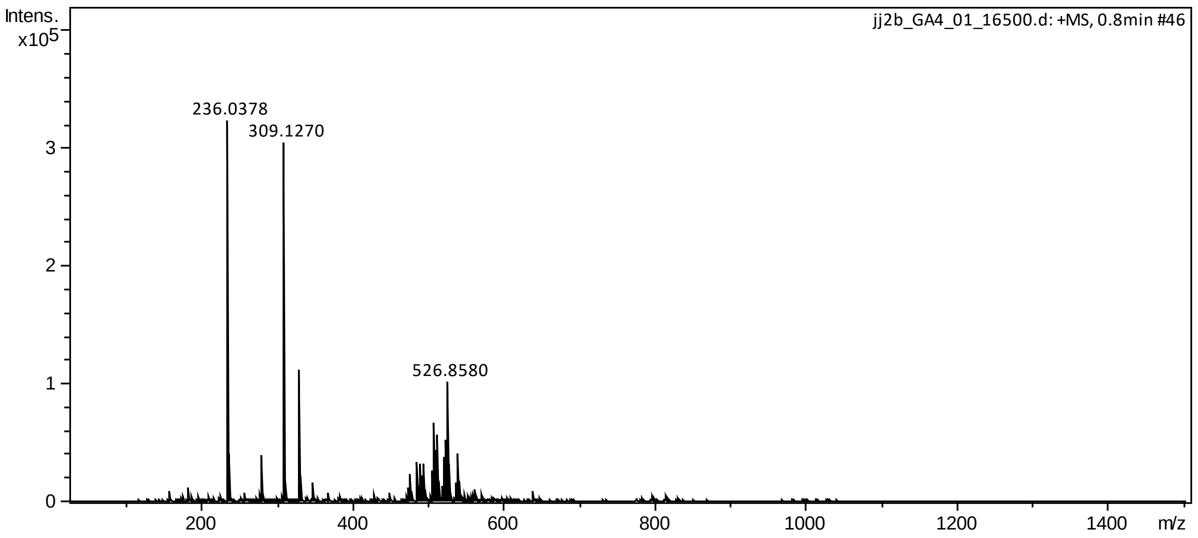

Compound **2b**, HRMS ESI$^+$: calc $[C_{15}H_{19}N_2O_3S+H]^+$: 309.1267.

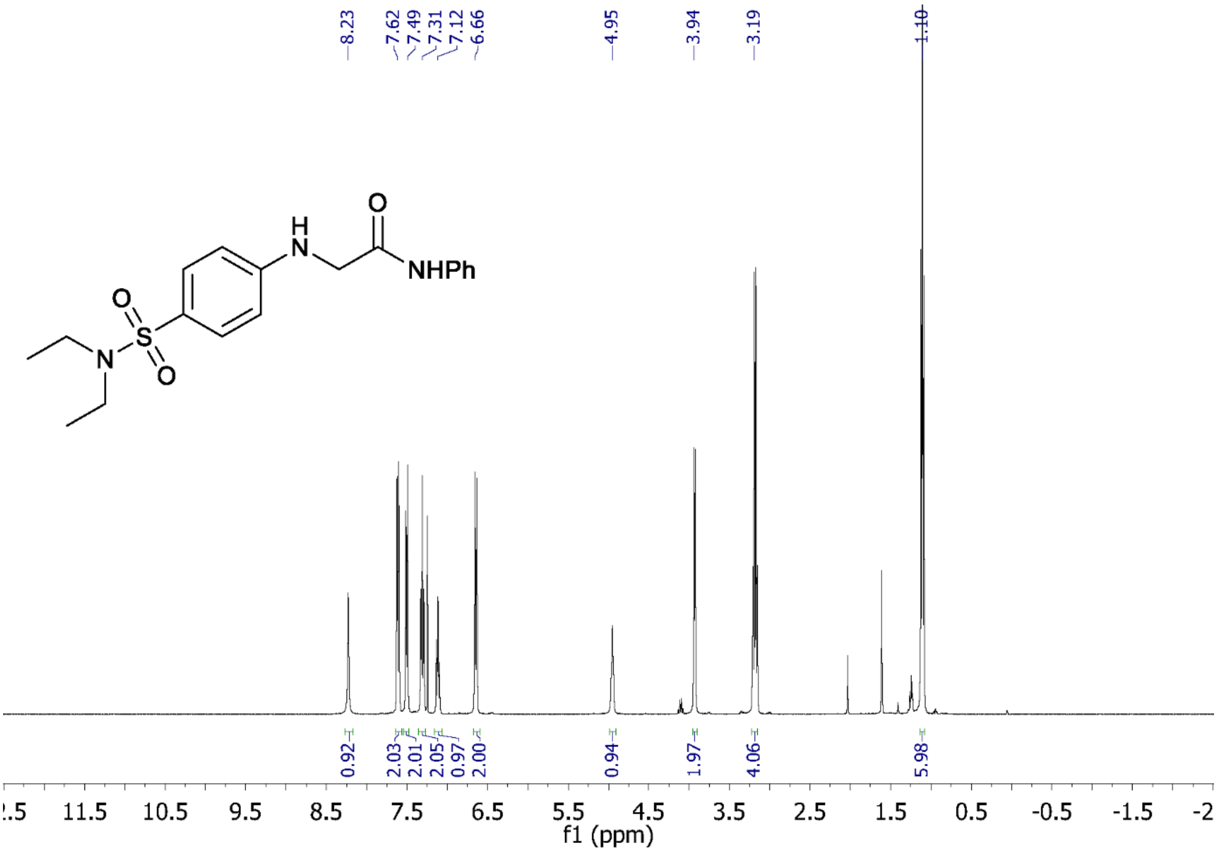

Compound **2c**, ^{1}H NMR (400 MHz, $CDCl_3$).

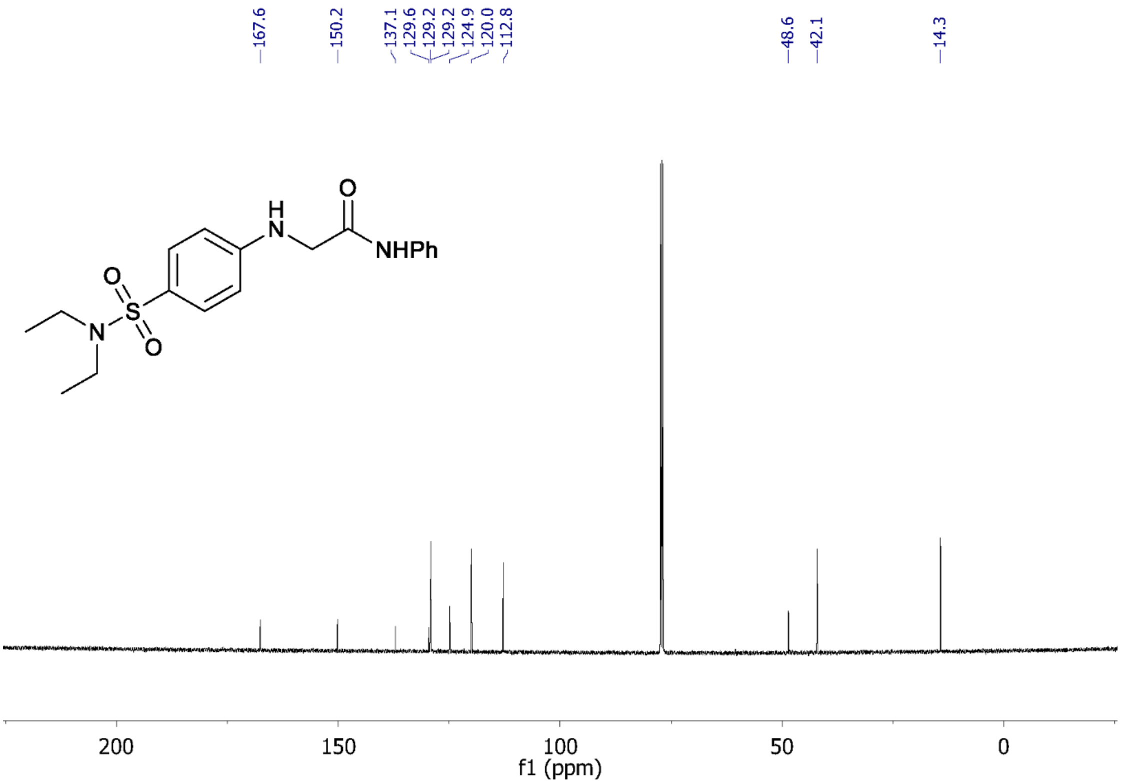

Compound **2c**, ^{13}C NMR (400 MHz, $CDCl_3$).

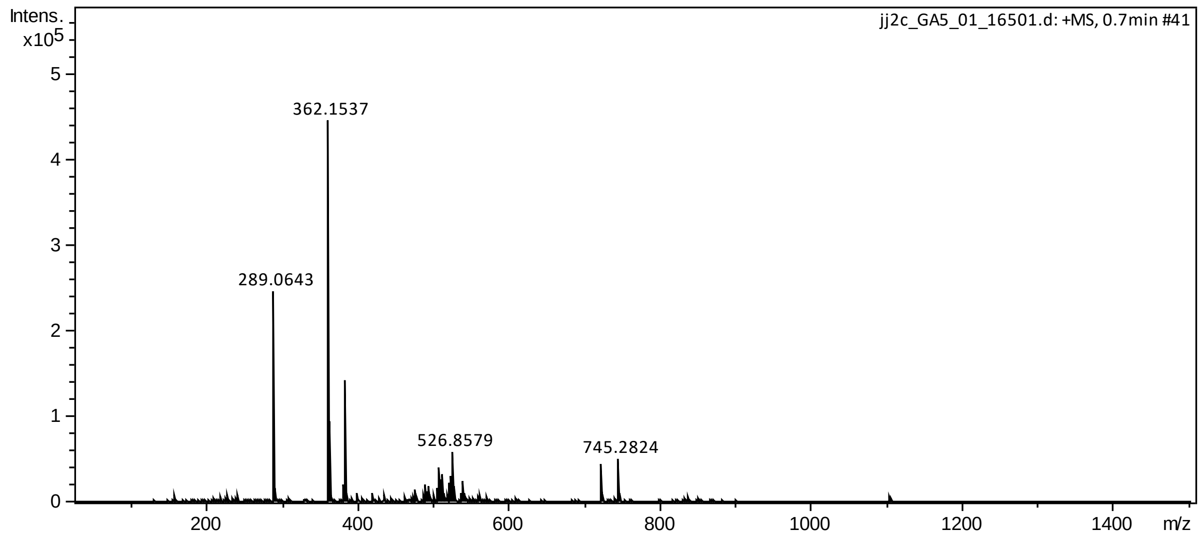

Compound **2c**, HRMS ESI$^+$: $[C_{18}H_{23}N_3O_3S+H]^+$ calc 362.1531.

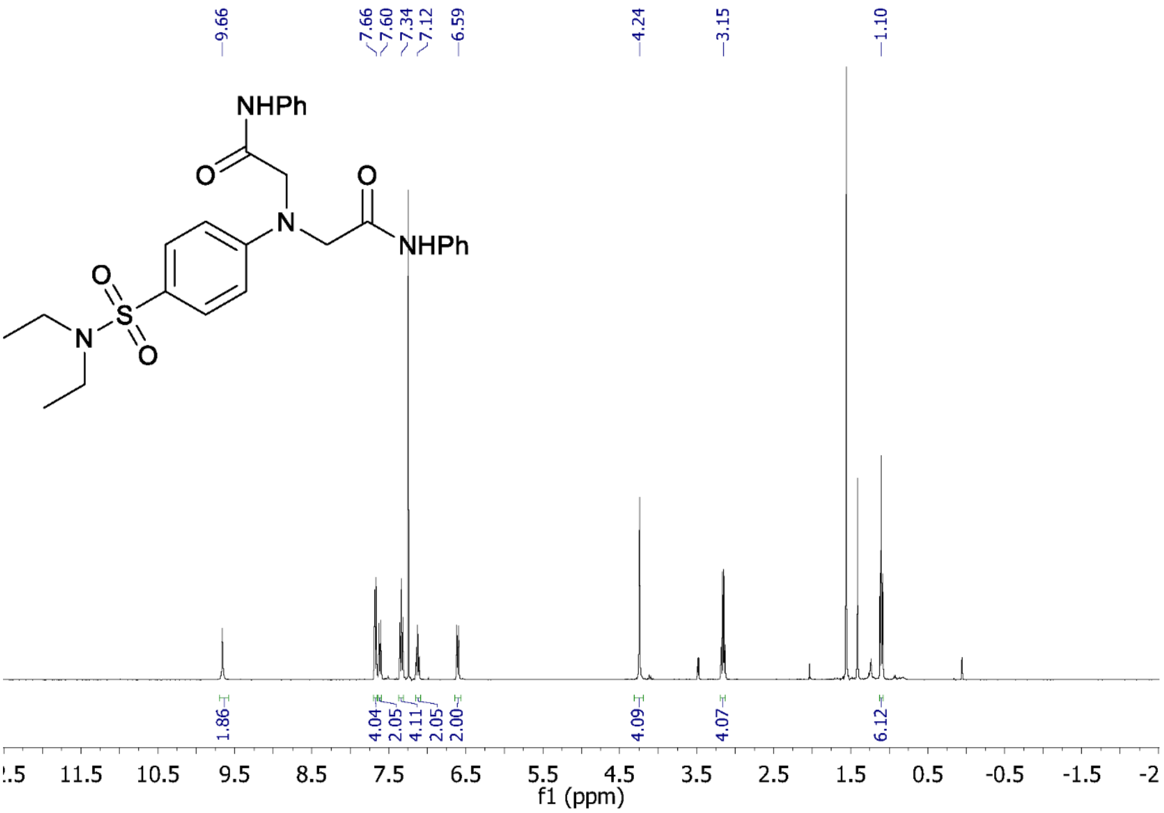

Compound **2d**, ^{1}H NMR (400 MHz, $CDCl_3$).

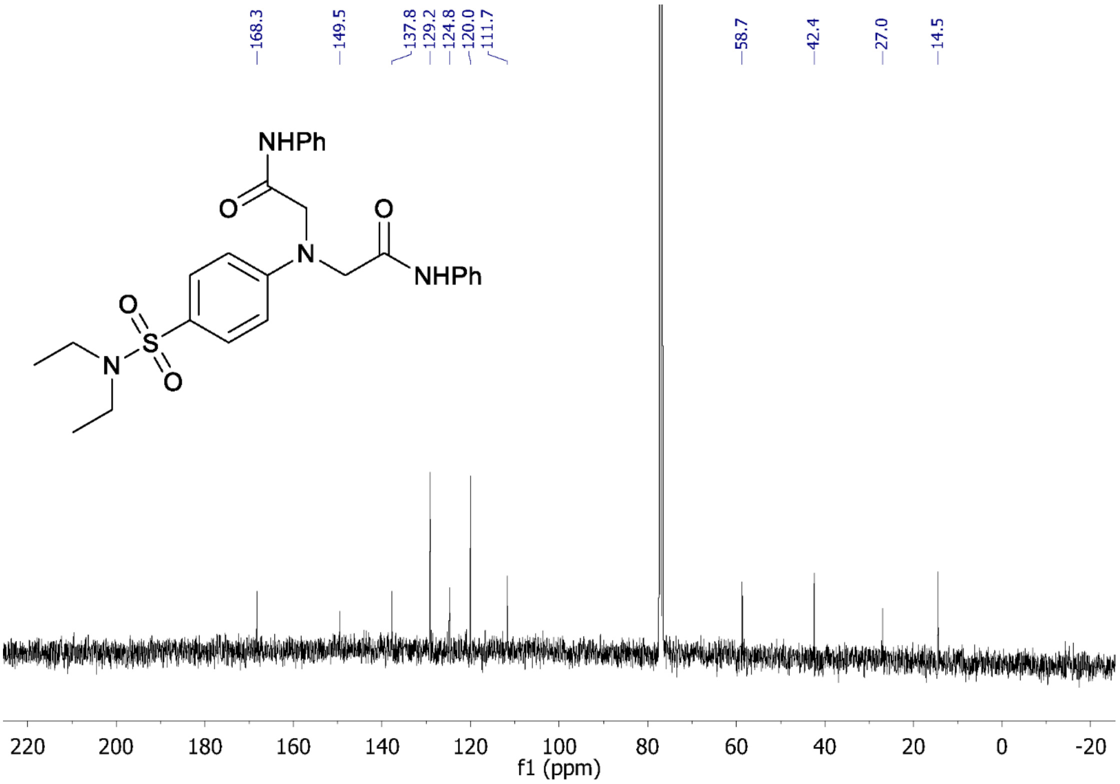

Compound **2d**, ^{13}C NMR (400 MHz, $CDCl_3$).

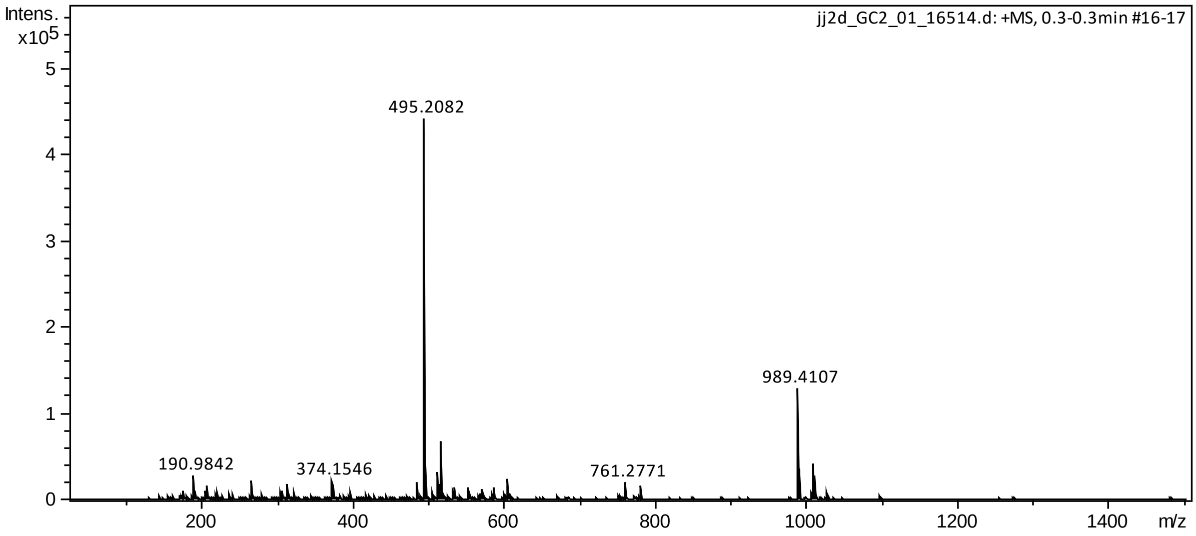

Compound **2d**, HRMS ESI$^+$: $[C_{26}H_{30}N_4O_4S+H]^+$ calc 495.2080.

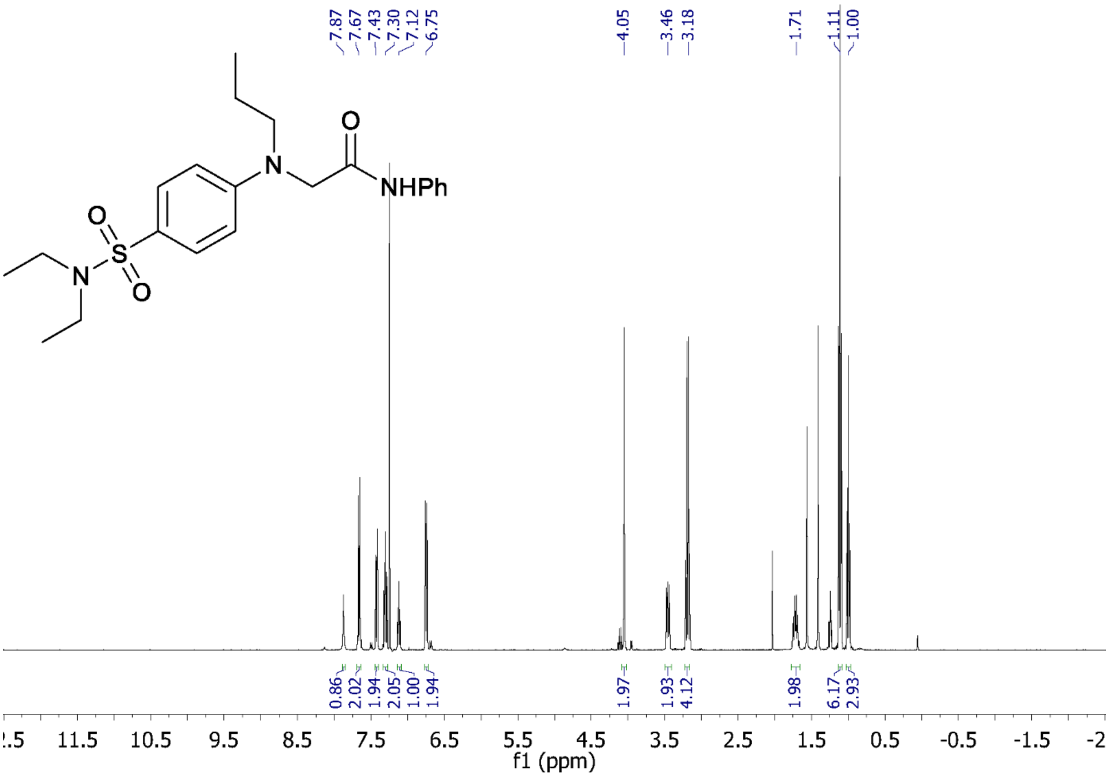

Compound **2e**, ^1H NMR (400 MHz, $CDCl_3$).

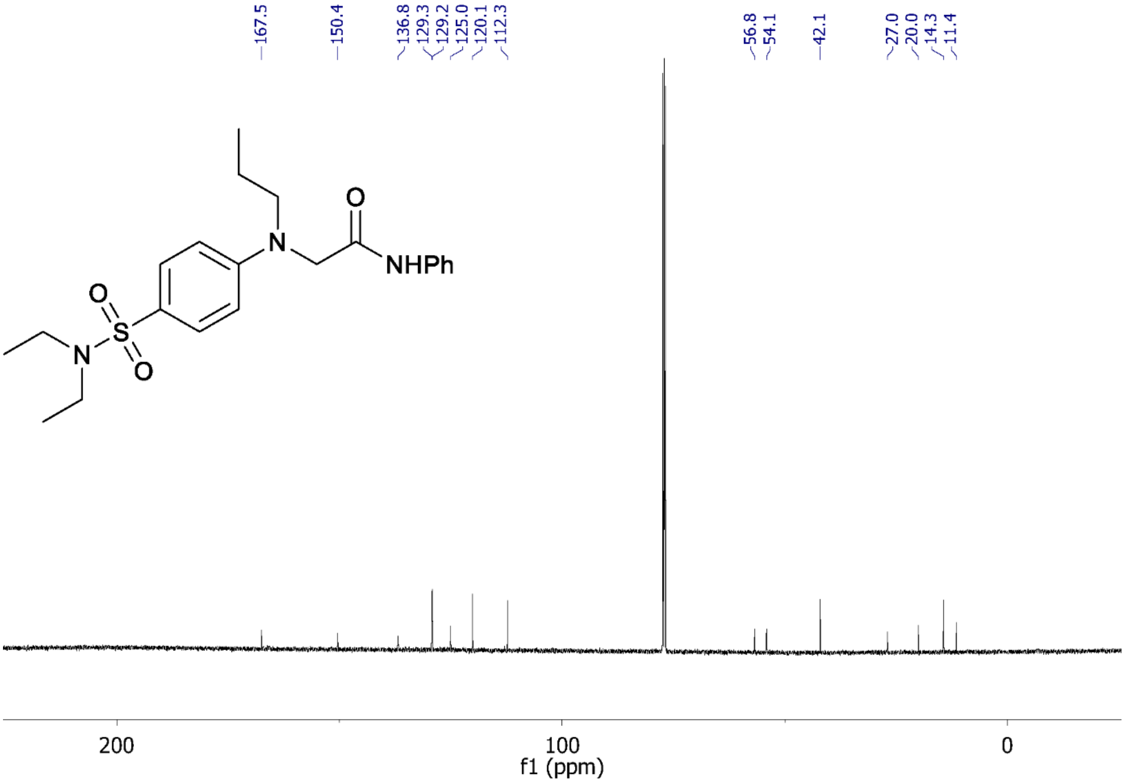

Compound **2e**, ^{13}C NMR (400 MHz, $CDCl_3$).

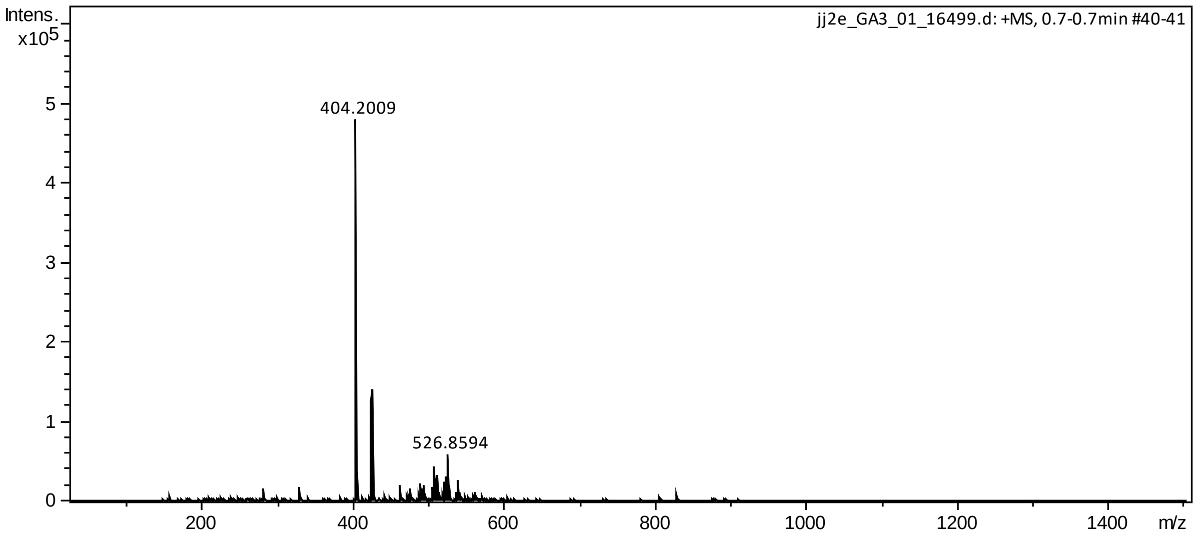

Compound **2e,** HRMS ESI$^+$: $[C_{21}H_{29}N_3O_3S+H]^+$ calc 404.2002.

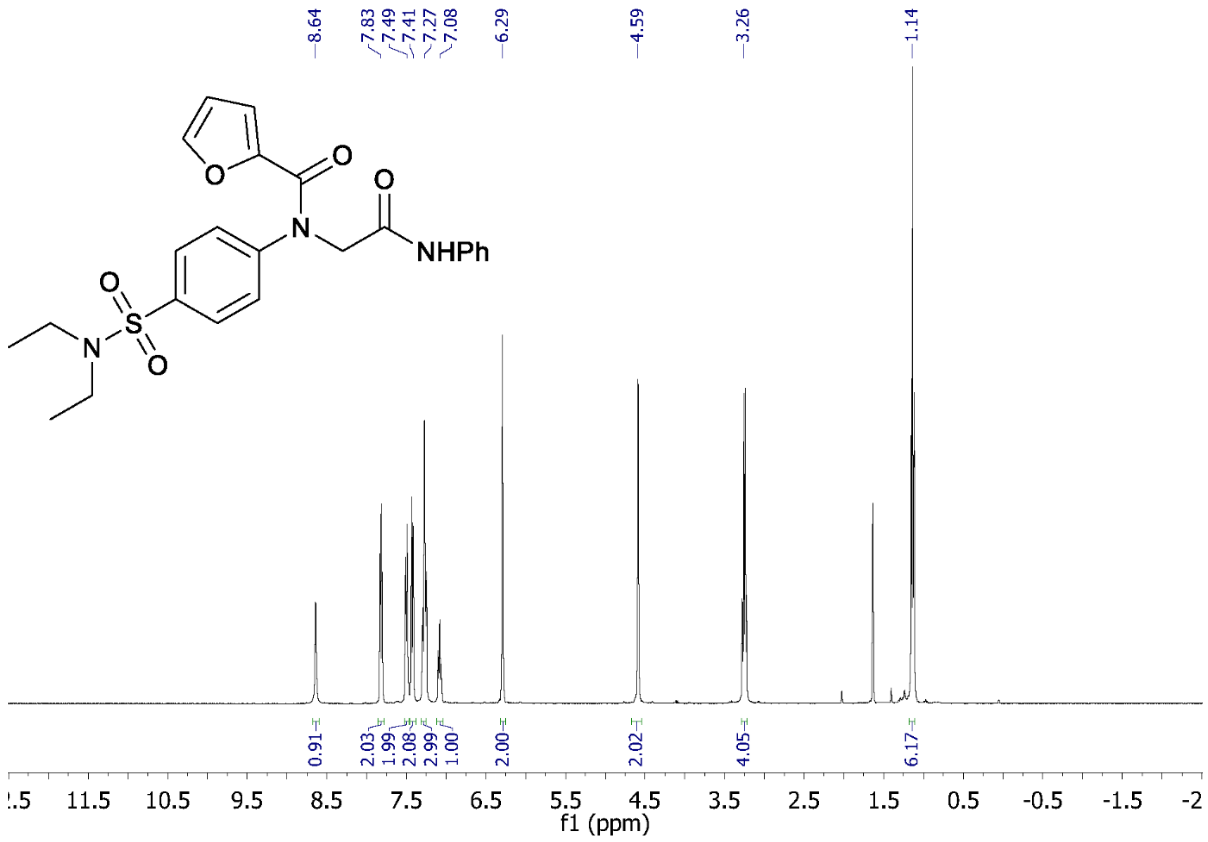

Compound **2f**, ^{1}H NMR (400 MHz, $CDCl_3$).

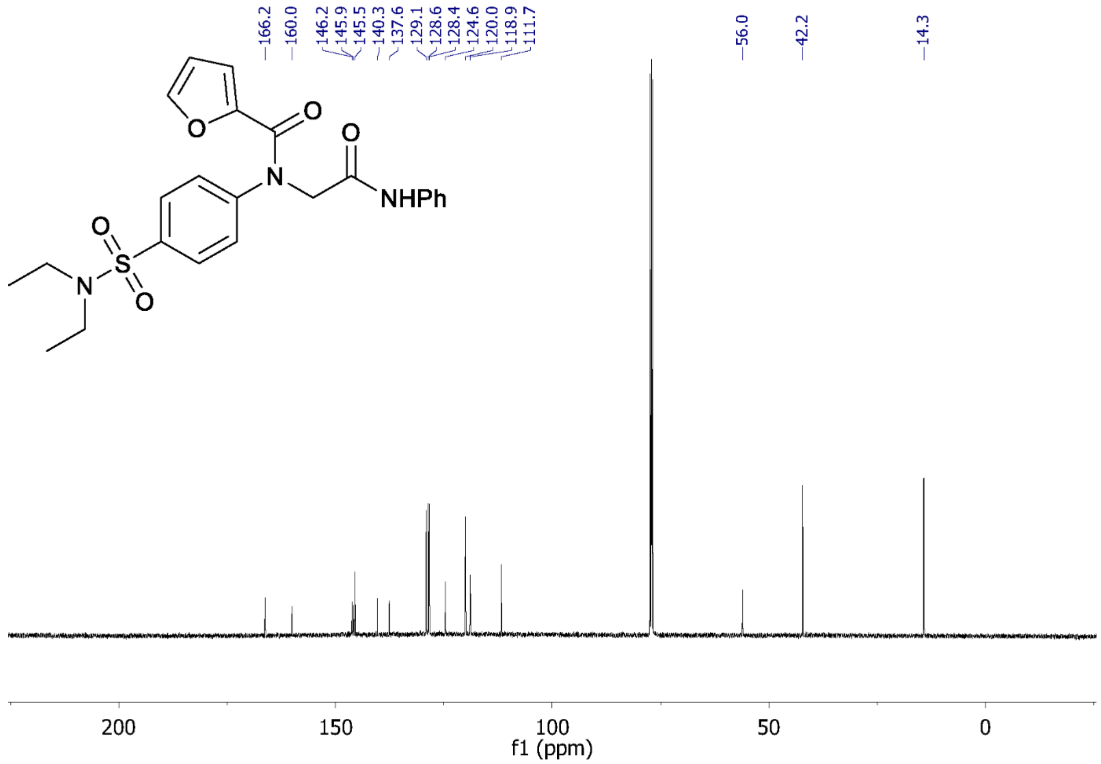

Compound **2f**, ^{13}C NMR (400 MHz, $CDCl_3$).

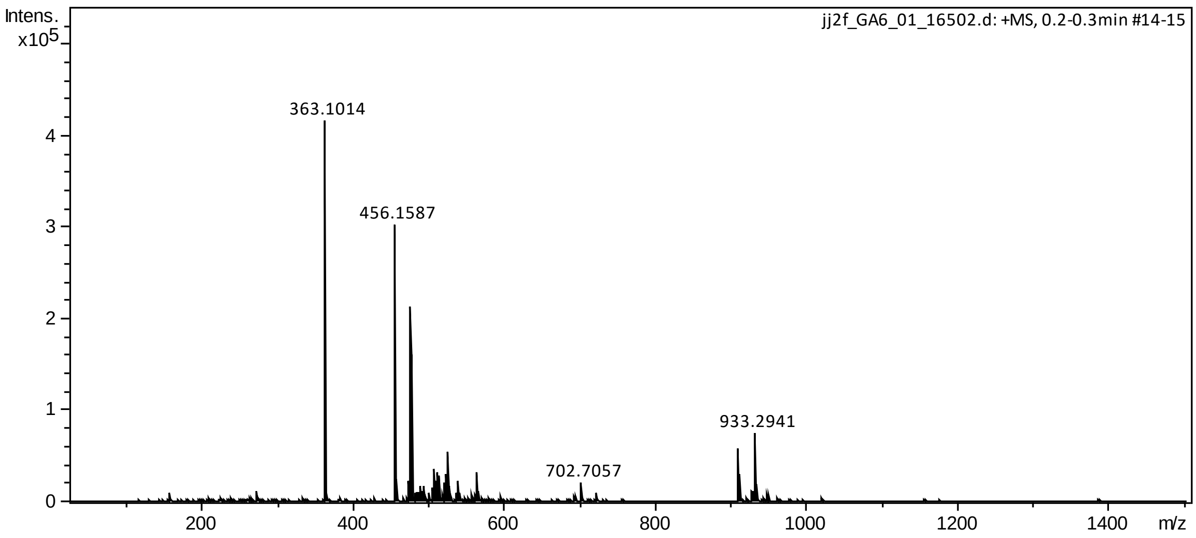

Compound **2f**, HRMS ESI$^+$: calc $[C_{23}H_{25}N_{33}O_5S+H]^+$: 456.1587.

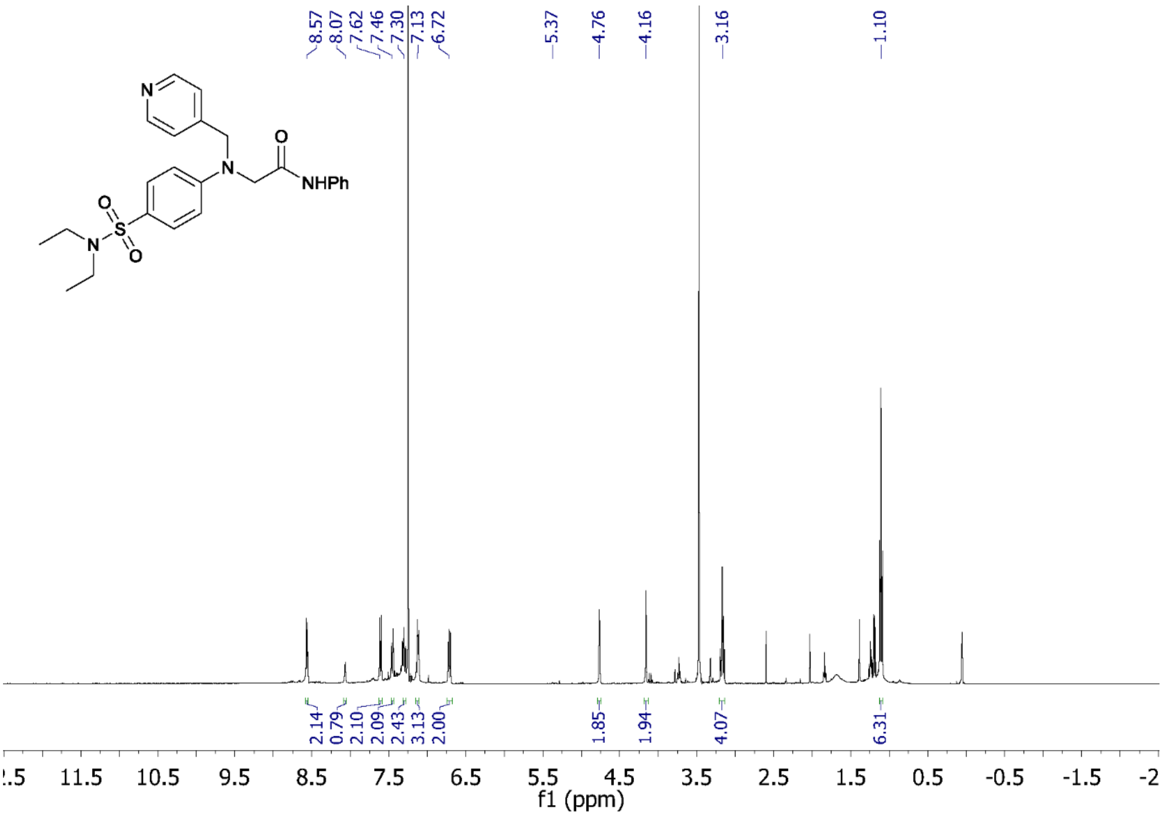

Compound **2g**, ^1H NMR (400 MHz, $CDCl_3$).

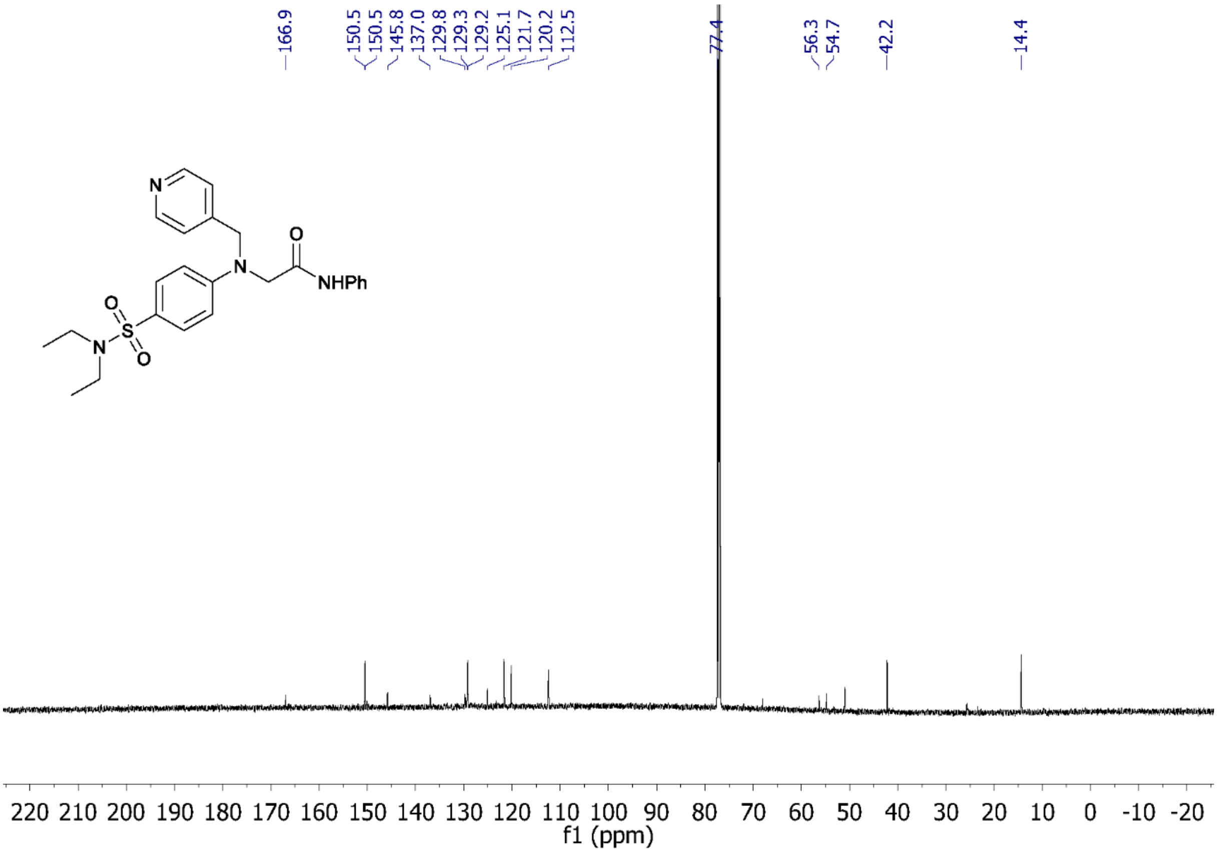

Compound **2g**, ^{13}C NMR (400 MHz, $CDCl_3$).

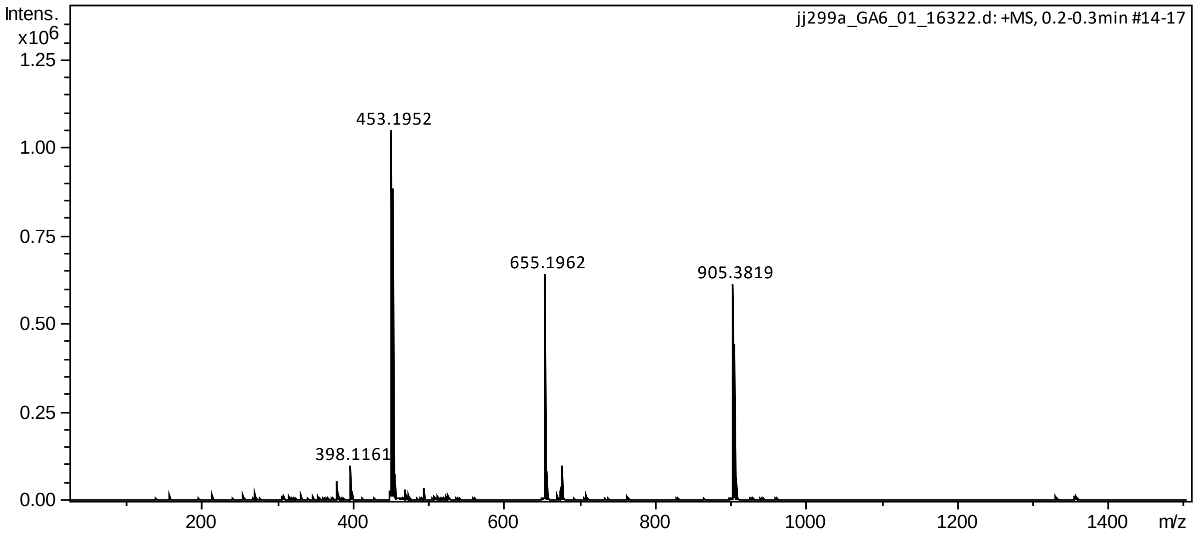

Compound **2g**, HRMS ESI$^+$: $[C_{24}H_{28}N_4O_3S+H]^+$ calc 453.1956.

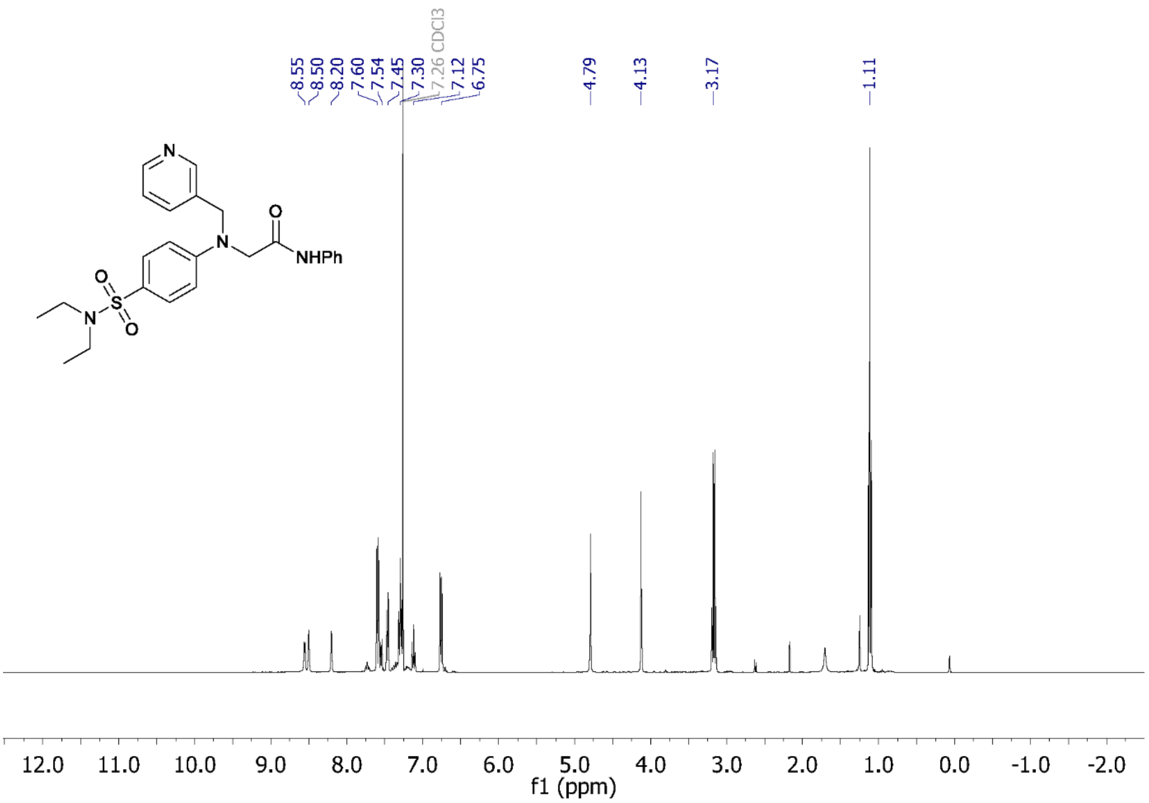

Compound **2h**, ^{1}H NMR (400 MHz, $CDCl_3$).

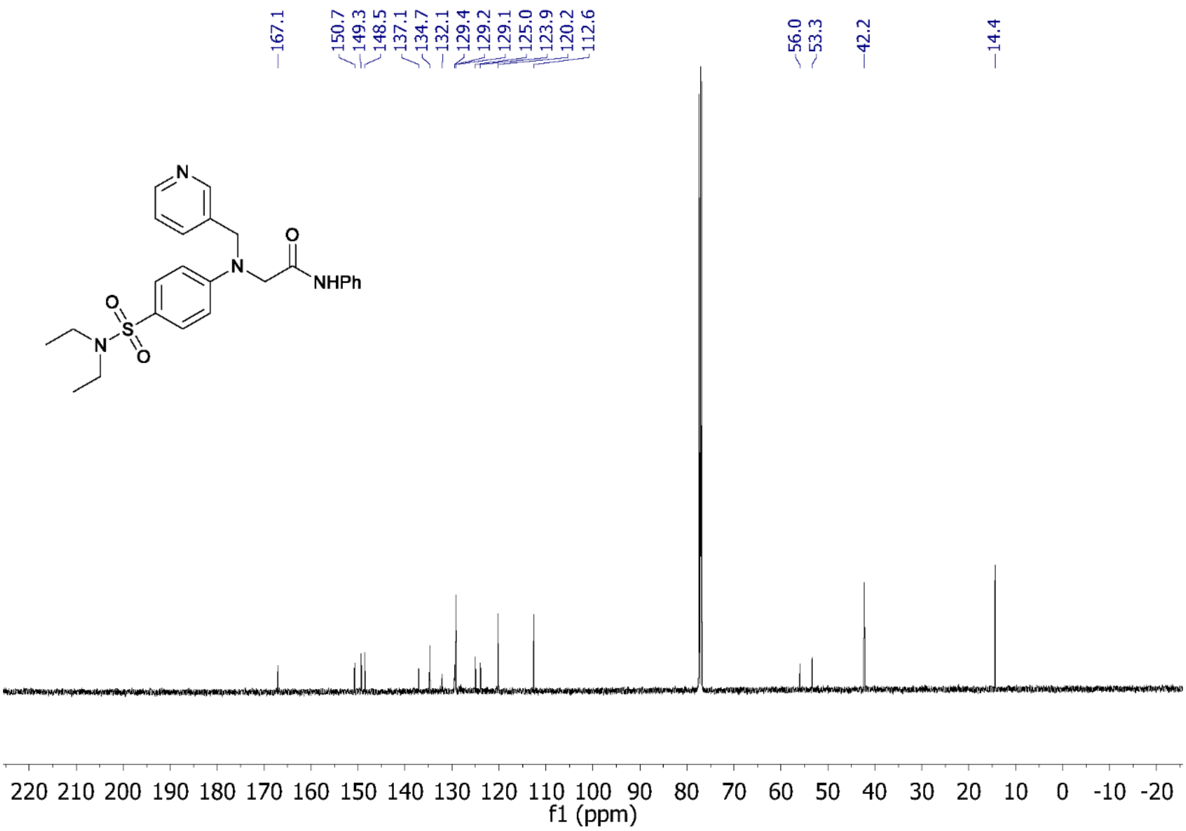

Compound **2h**, ^{13}C NMR (400 MHz, $CDCl_3$).

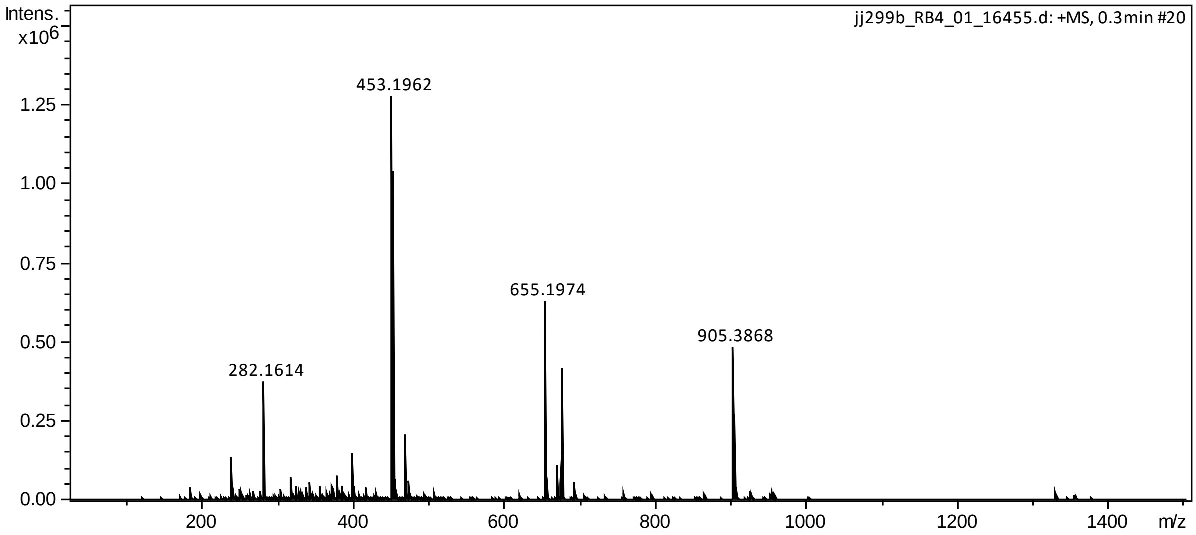

Compound **2h**, HRMS ESI$^+$: $[C_{24}H_{28}N_4O_3S+H]^+$ calc 453.1956.

2. Molecular docking

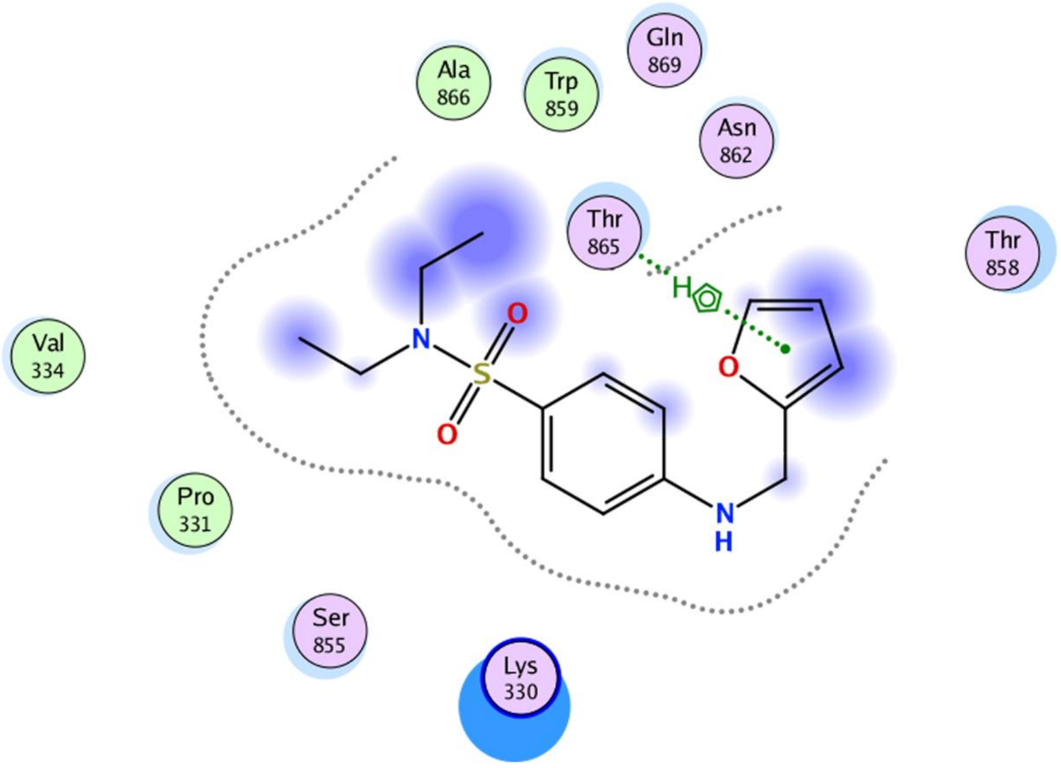

Figure S1. Molecular docking of **2b** to the binding pocket of DENV NS5.

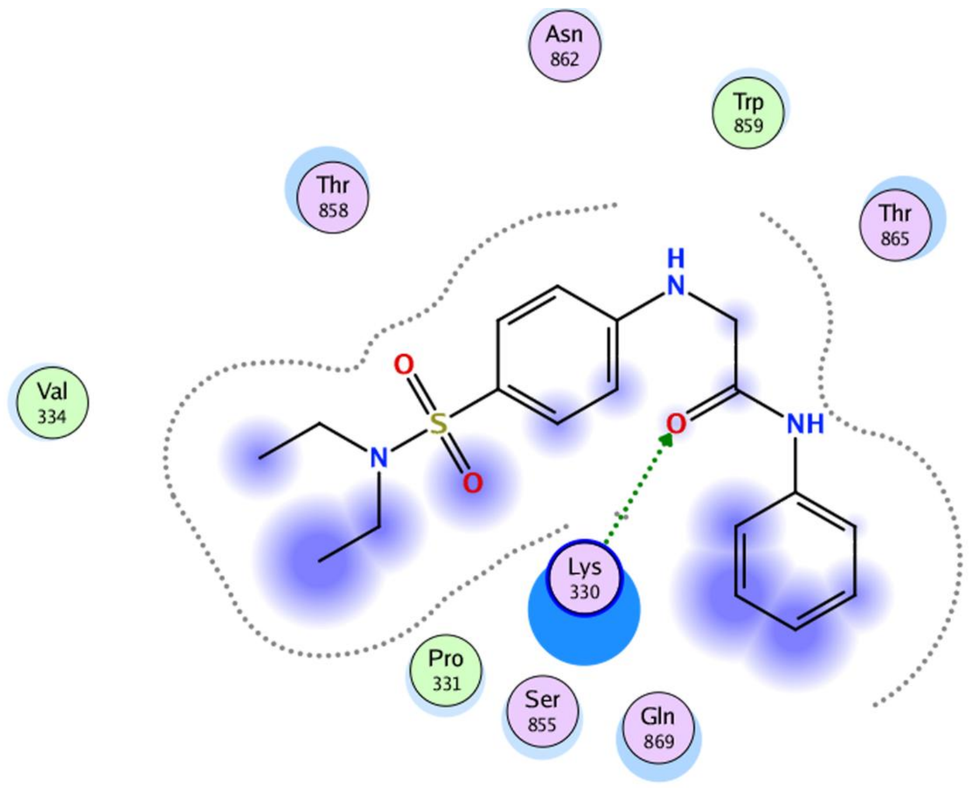

Figure S2. Molecular docking of **2c** to the binding pocket of DENV NS5.

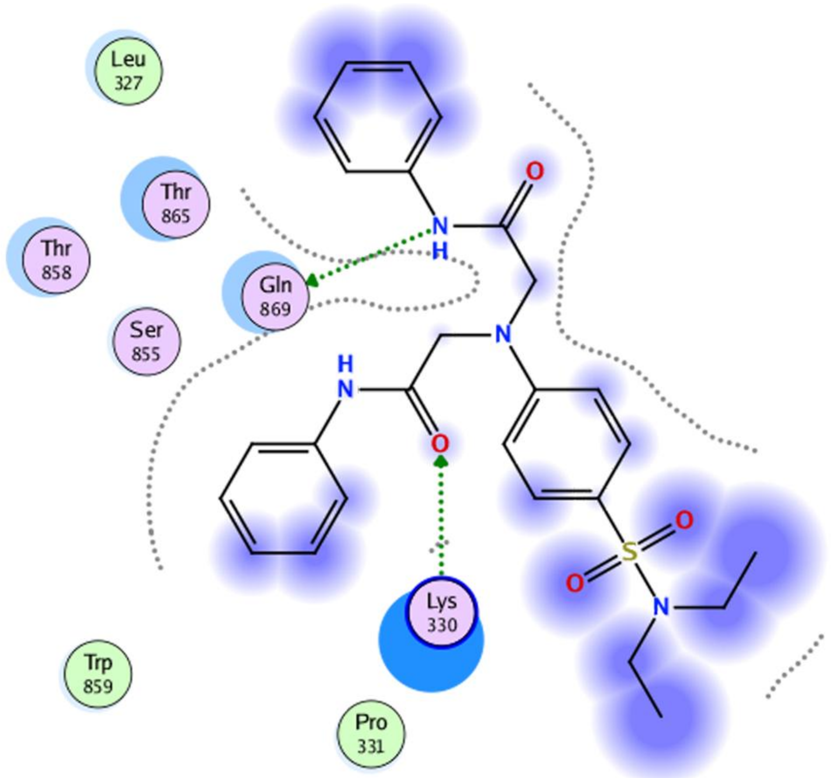

Figure S3. Molecular docking of **2d** to the binding pocket of DENV NS5.

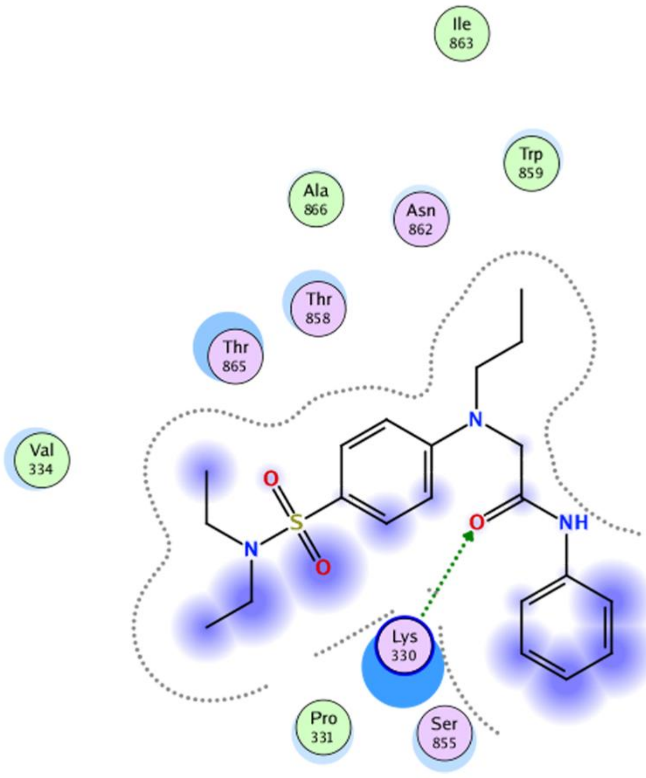

Figure S4. Molecular docking of **2e** to the binding pocket of DENV NS5.

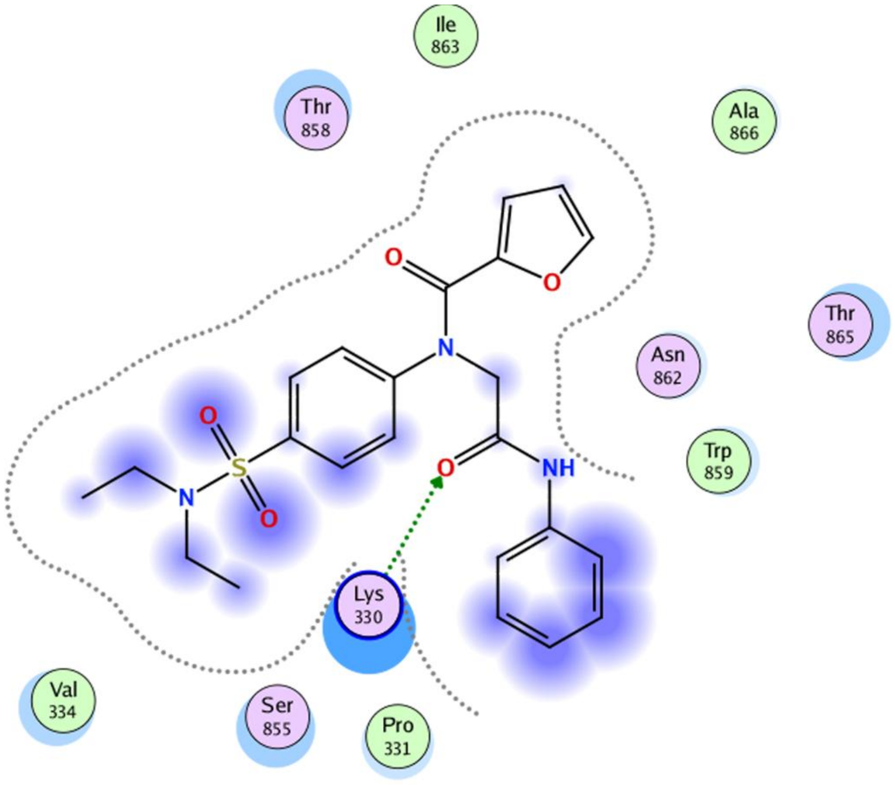

Figure S5. Molecular docking of **2f** to the binding pocket of DENV NS5.

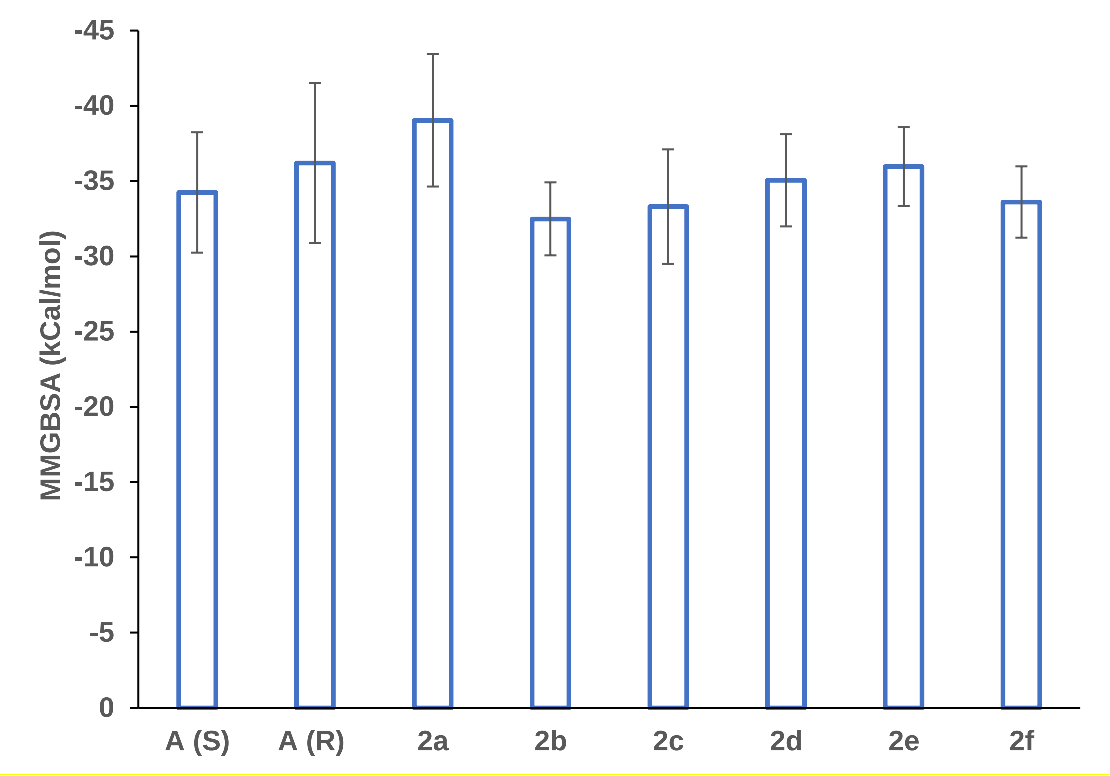

Figure S6. Average MMGBSA (kCal/mol) with standard deviations for compounds **A** and **2a-f**.

3. Results of biological testing

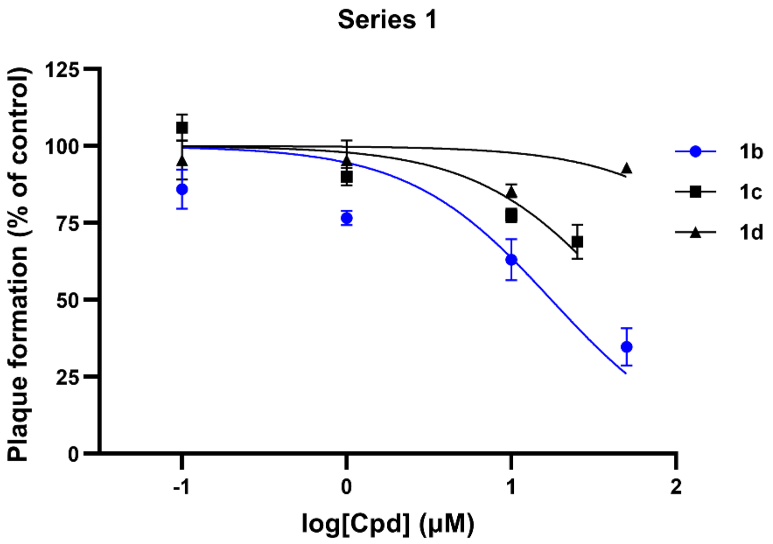

Figure S7. Concentration-dependent inhibition of DENV-2 replication in Vero cells by compounds belonging to Series **1** as determined by PRA. Data represent the mean of n ≥ 2 independent experiments in duplicate and were analyzed by nonlinear regression function of GraphPad Prism 10.0.

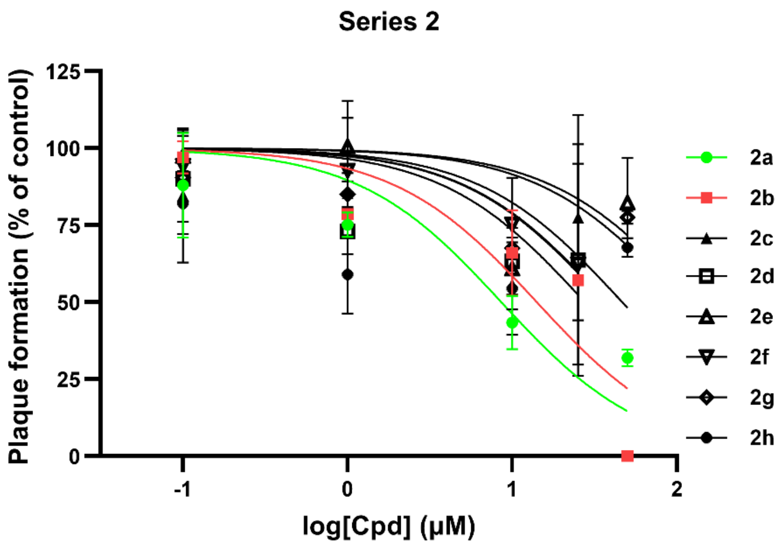

Figure S8. Concentration-dependent inhibition of DENV-2 replication in Vero cells by compounds belonging to Series **2** as determined by PRA. Data represent the mean of n ≥ 2 independent experiments in duplicate and were analyzed by nonlinear regression function of GraphPad Prism 10.0.
